# Correction: Plasma proteomic signatures of early retinal neurodegeneration in diabetes: a multi-cohort study

**DOI:** 10.1371/journal.pmed.1005180

**Published:** 2026-07-16

**Authors:** The PLOS Medicine Staff

## Notice of Republication

This article was republished on June 26, 2026, to correct errors in the title that were introduced during the typesetting process. The publisher apologizes for the errors.

The correct citation is: Li H, Zhu Z, Yang S, Cheng W, Tan S, Xin Z, et al. (2026) Plasma proteomic signatures of early retinal neurodegeneration in diabetes: a multi-cohort study. PLoS Med 23(6): e1004868. https://doi.org/10.1371/journal.pmed.1004868

Please download this article again to view the correct version. The originally published, uncorrected article and the republished, corrected articles are provided here for reference.

## Supporting information

S1 File Originally published, uncorrected article. (PDF)

S2 File Republished, corrected article. (PDF)
