# Supplementary material for: Plasma proteomic signatures of early retinal neurodegeneration in diabetes: a multi-cohort study
Source: PLoS Med. 2026 Jun 2;23(6):e1004868. doi: 10.1371/journal.pmed.1004868 (PMC13229346; doi:10.1371/journal.pmed.1004868)
Supplement: S1 Text — (DOCX) [file pmed.1004868.s003.docx]

# S1 Text

# Supplementary Methods

## Inclusion and Exclusion Criteria

Analysis populations were derived from two prospective cohorts: GDES-PPP and UKB-PPP. In GDES-PPP, sample selection proceeded in two stages. First, for the cross-sectional analysis, we included 1,492 baseline participants with both qualified plasma proteomics and OCT data. OCT eligibility required signal strength ≥60, absence of fixation loss or segmentation errors, and no ocular conditions that could confound measurements, including glaucoma, retinal vascular disease, or high refractive error (spherical equivalent > ±6.00 D or axial length ≥26 mm), and no baseline dementia or major neurodegenerative disorders. Participants also required complete key covariate data. This population served to preliminarily screen plasma proteins associated with RNFL thickness. Second, to rigorously assess neurodegeneration while minimizing confounding from vascular or exudative lesions, we selected participants who completed the full 6-year follow-up without developing diabetic retinopathy. This yielded 1,218 continuously vascular-lesion–free individuals for longitudinal analyses, enabling validation of protein associations with RNFL thinning rate, pathway enrichment analysis, and construction of machine-learning models. For external validation, 502 UKB-PPP participants with diabetes at baseline, no retinal disease, and available proteomics and OCT data were selected. Exclusion criteria mirrored those applied in GDES, allowing assessment of cross-ethnic robustness of the findings.

## OCT Imaging Quality Control

All OCT scans underwent standardized quality control (QC) using multiple metrics, as previously described[1-3]: Image Quality Score (IQS), internal limiting membrane (ILM) indicators, validity counts, and motion-artifact indices. IQS quantified signal strength, ILM indicators detected blink artifacts and segmentation errors, validity counts assessed Z-axis truncation, and motion-artifact indices were calculated from Pearson correlations and absolute differences of RNFL and full-retina thickness between consecutive B-scans to identify eye movement, artifacts, or segmentation errors. In GDES, automated segmentation results were reviewed by image experts, and all participant data were processed blinded. Only high-quality scans with clearly discernible retinal layer structures were retained. Scans were excluded if they exhibited any of the following: (1) signal strength ≤60, (2) defocus, (3) motion or blink artifacts, (4) off-center alignment, (5) poor contrast due to media opacities (e.g., local signal loss, blurring, masking), or (6) uncorrectable segmentation errors. In UKB, images were excluded if overall quality was <40 or ILM metrics fell within the worst 10%, following previously established pipelines.

## Normative reference cohort for RNFL decline

To anchor the “excessive RNFL loss” phenotype to physiological ageing, we used the Chinese Ocular Imaging Project (COIP), a community-based cohort that consecutively recruited individuals with diabetes mellitus and healthy participants from the Guangzhou community between November 2017 and December 2018. Participants underwent comprehensive ocular and systemic examinations, and the absence of retinopathy and optic neuropathy was confirmed using ETDRS 7-field fundus photography and OCT, with annual follow-up examinations.

In COIP, after pupil dilation, macular and optic-disc imaging was performed by trained examiners using a swept-source OCT device (DRI OCT Triton; Topcon, Japan), including a disc-centered peripapillary ring scan. Images with a quality score <60, segmentation errors, artifacts (including blink-related dark areas), eccentric scans, or missing scan lines were excluded.

For the present study, we restricted the reference sample to non-diabetic (healthy) participants in COIP and used the longitudinal OCT data available over up to 6 years to derive a normative distribution of annualized RNFL thinning slopes. Annualized RNFL thinning rates were estimated using the same modelling strategy as in the main analysis. We defined DRN-ExcessLoss as an annualized RNFL thinning rate below the lower reference limit of the normative distribution (mean − 1.96 SD), indicating RNFL loss faster than expected from physiological ageing.

## Olink Assay and NPX Transformation

Proteomic profiling in GDES was performed on a randomly selected 50% of baseline plasma samples (~3,000 participants) to form the proteomic sub-cohort (GDES-PPP) for Olink assays. Samples were aliquoted after standardized thawing and shipped to Olink laboratories for synchronous measurement using the Cardiometabolic Panel. For Olink Explore assays, the sample consumption is low (approximately 2.8 µL plasma per 384-plex panel, and ~6 µL per sample for the full Explore 3072 library), while the plated volume is higher to accommodate automated handling and dead volume. UKB proteomic data were derived from the UKB-PPP project (~50,000 participants). High-sensitivity quantification of plasma proteins was achieved via Olink proximity extension assay (PEA), in which paired antibodies linked to complementary oligonucleotides bind the target protein, allowing hybridization, extension, and relative quantification via next-generation sequencing.

Olink quality control includes multiple internal controls: incubation (non-human antigen with matched antibody), extension (IgG-bound oligonucleotide pair), amplification (synthetic dsDNA), negative controls, and plate controls. QC warnings are flagged if the incubation control deviates from the plate median by ±0.3, although values below the detection limit are retained for analysis. Protein expression is reported as NPX. Proteins with ≥30% missing values were excluded, and remaining missing data were imputed using the median. Both cohorts applied identical NPX normalization workflows and corrected for batch effects, following Olink’s standard PEA NGS pipeline and recommended NPX processing to ensure comparability with prior large-scale Olink studies (including UKB-PPP) and to facilitate cross-cohort replication, ensuring reproducibility with GDES-PPP as the discovery cohort and UKB-PPP as the validation cohort.

NPX values undergo multi-stage processing to achieve cross-sample and cross-plate comparability[4-6]. First, baseline correction is performed using each sample’s extension control:

$$Ext{NPX}_{i,j}={log}_{2}\left( \frac{counts\left( {sample}_{j}{Assay}_{i} \right)}{Counts\left( {ExtCtrl}_{j} \right)} \right)$$

where ​​$i$​ denotes the target protein and $j$ the sample. Log₂ transformation mitigates extreme values and approximates normality. Second, within-plate variation is corrected by subtracting the median ExtNPX of the plate control:

$${NPX}_{i,j}=Ext{NPX}_{i,j}-median\left( ExtNPX\left( {PlateCtrl}_{i} \right) \right)$$

Finally, global batch correction aligns each plate to a reference using the plate median NPX, yielding inter-plate normalized NPX:

$${NPX}_{{Intnorm}_{i,j}}={NPX}_{i,i}-plate median\left( {NPX}_{i} \right)$$

This workflow ensures comparability across plates while preserving biological variation.

## UKB Covariate Assessment

At baseline (2006–2010), UKB collected comprehensive data through physical measurements, face-to-face interviews, and detailed touchscreen questionnaires. Questionnaires captured (1) sociodemographic factors, including age, sex, ethnicity, education, and Townsend Deprivation Index (TDI); (2) lifestyle factors, including smoking, alcohol consumption, sleep, diet, and physical activity; and (3) health status, including family history, use of lipid-lowering or antihypertensive medications, and prior disease diagnoses. Baseline diseases were defined by integrating ICD-10 codes with questionnaire, interview, and hospitalization data.

Physical and laboratory measures included body mass index (BMI), waist circumference, blood pressure, HDL-C, triglycerides, HbA1c, fasting glucose, serum creatinine, serum urate, urinary albumin, alanine aminotransferase (ALT), aspartate aminotransferase (AST), gamma-glutamyl transferase (GGT), and hemoglobin. Serum biomarkers were assayed using 10 immunoassay analyzers (6 × DiaSorin Liaison XL, 4 × Beckman Coulter DXI-800) and 4 clinical chemistry analyzers (2 × Beckman Coulter AU5800, 2 × Siemens Advia 1800).

Covariate Grouping and Definitions

- **Age:** categorized as <50, 50–54, 55–59, 60–64, and >64 years.
- **Townsend Deprivation Index (TDI):** derived from participants’ postal code areas, reflecting unemployment, household overcrowding, car ownership, and homelessness; grouped into quartiles (< −3.6, −3.6 to −2.1, −2.1 to 0.6, >0.6) and missing.
- **BMI:** calculated from baseline height and weight using a Seca 240 cm stadiometer and Tanita BC418MA analyzer; categorized as <25, 25–29.9, ≥30.0, and missing.
- **Ethnicity:** self-reported as White, Mixed, Asian, Black, Chinese, or missing; the latter five categories were combined into a single non-White/minority group due to small counts.
- **Household income:** pre-tax average household income grouped as <£18,000, £18,000–30,999, £31,000–51,999, £52,000–100,000, >£100,000, and missing.
- **Education:** highest qualification grouped as O levels (or equivalent), A levels (or equivalent), college/university degree, and missing.
- **Smoking and alcohol:** categorized as never vs former/current smokers and never vs past/current drinkers, respectively.

HbA1c Measurement: HbA1c was quantified using five Bio-Rad Variant II Turbo analyzers, employing high-performance liquid chromatography (HPLC) to determine relative HbA1c concentration in processed packed red blood cells (PRBC). Prior to analysis, a rigorous validation program—including matrix validation for PRBC, multi-instrument comparisons, and inter-instrument consistency testing—ensured compliance with ISO 17025:2005 standards and agreement across all instruments.

## Decision curve analysis

Decision curve analysis (DCA) was used to assess clinical utility by estimating net benefit across a range of threshold probabilities and comparing model-based strategies with default strategies of intervening in all individuals (treat all) or in none (treat none). Net benefit was calculated using the standard formulation proposed by Vickers and Elkin:

$$Net benefit=(\mathrm{TP}/n)-(\mathrm{FP}/n)\times(\mathrm{pt}/(1-\mathrm{pt}))$$

where TP and FP denote the numbers of true and false positives at a given threshold probability pt, and n is the total sample size. The threshold probability pt represents the minimum predicted risk at which an intervention (for example, intensified monitoring or referral) would be considered, and therefore encodes the relative weighting of false positives versus true positives through pt/(1−pt).

## Modelling Approaches for DRN Proteomics

We trained eight supervised models on the GDES cohort to predict diabetic retinal neurodegeneration (DRN) from discovery-stage proteomic features. Data were split by stratified random sampling (80% training, 20% held-out test). All preprocessing and hyperparameter tuning were performed strictly within the training set using 10-fold stratified cross-validation (CV), with area under the curve (AUC) as the primary metric. Operating thresholds were selected in CV via Youden’s J statistic and fixed for the test set.

**Extreme Gradient Boosting (XGBoost):** We used the binary-logistic gbtree formulation, in which predicted probabilities are obtained via a sigmoid link and the objective combines logistic loss with structural and weight regularization[7]:

$$\begin{aligned} \mathcal{L}=\sum_{i=1}^{N} \left[ -y_{i}\mathrm{lo}g \hat{p}_{i}-\left( 1-y_{i} \right)\mathrm{lo}g \left( 1-\hat{p}_{i} \right) \right]+\sum_{t=1}^{T} \left( \gamma\left| \mathcal{L}_{t} \right|+\frac{\lambda}{2}\parallel w_{t}\parallel_{2}^{2}+\alpha\parallel w_{t}\parallel_{1} \right)\# \left( 1 \right) \end{aligned}$$

We adopted a moderate learning rate (0.05), tree depth capped at 7, minimum loss-reduction margin γ=2, and stochasticity through 90% row subsampling per boosting round and ~50% column subsampling per level. Mild L1/L2 penalties (α=0.5, λ=1) and a minimum child-weight constraint-controlled overfitting. Class weights were equal. Early stopping under 10-fold CV selected 19 boosting rounds, with 71 proteomic features entering the final model.

**Light Gradient Boosting Machine (LightGBM):** We employed leaf-wise histogram-based gradient boosting for binary classification, minimizing negative log-likelihood[8]:

$$\begin{aligned} \mathcal{l}_{\mathrm{logistic}}\left( y_{i},\hat{p}_{i} \right)=-y_{i}\mathrm{lo}g \hat{p}_{i}-\left( 1-y_{i} \right)\mathrm{lo}g \left( 1-\hat{p}_{i} \right)\#\left( 2 \right) \end{aligned}$$

We used a moderate learning rate (~0.045), leaf complexity limited to 31 leaves (max depth 7), minimum 30 samples per leaf, row and feature subsampling (~75%), early stopping, and equal class weights.

**Random Forest:** Standard bagging of decision trees with random feature subsetting per split was applied. Splits minimized Gini impurity[9]:

$$\begin{aligned} G\left( S \right)=1-\sum_{k} p_{k}^{2}\#\left( 3 \right) \end{aligned}$$

We used ~500 trees with bootstrap sampling and out-of-bag error estimation, candidate features per split followed the square-root rule, and minimum samples per leaf constrained depth; class weights were equal.

**Support Vector Machine (SVM):** We applied a soft-margin classifier with radial basis function (RBF) kernel[10]:

$$\begin{aligned} f\left( x \right)=\sum_{i=1}^{n} \alpha_{i}y_{i}K\left( x_{i},x \right)+b\#\left( 4 \right) \end{aligned}$$

Kernel width was automatically determined from feature spread, margin penalty set moderately (~1), probabilities obtained via Platt scaling, and class-balanced weights applied.

**Neural Network:** A single-hidden-layer multilayer perceptron with logistic hidden units and sigmoid output was trained to minimize binary cross-entropy[11]:

$$\begin{aligned} \mathcal{L}=-\sum_{i=1}^{N} \left[ \begin{aligned} y_{i}\log\hat{p}_{i}+\left( 1-y_{i} \right)\log\left( 1-\hat{p}_{i} \right) \end{aligned} \right]\#\left( 5 \right) \end{aligned}$$

We used a small hidden layer (8 units), L2 weight decay, PCA-based dimension reduction, and bagging across bootstrap resamples with random feature subsets to enhance stability. Class weights were equal.

**k-Nearest Neighbors (KNN):** A non-parametric classifier with distance-weighted voting was applied[12]:

$$\begin{aligned} \hat{p}\left( x \right)=\frac{\sum_{i\in\mathcal{N}_{k}\left( x \right)} w_{i}y_{i}}{\sum_{i\in\mathcal{N}_{k}\left( x \right)} w_{i}}\#\left( 6 \right) \end{aligned}$$

Weights decreased smoothly with distance using the Epanechnikov kernel, with neighborhood size K≈60, distance weighting, equal class weights, and parameter selection via stratified CV.

**Decision Tree:** A CART-style classifier used greedy splits based on information gain[8]:

$$\begin{aligned} \mathrm{IG}=H\left( \mathrm{parent} \right)-\sum_{c\in\left\{ L,R \right\}} \frac{n_{c}}{n}H\left( c \right)\#\left( 7 \right) \end{aligned}$$

where $H()$ denotes entropy. Depth, minimum samples to split, and leaf size were lightly constrained, with final tree pruned via cost–complexity CV; class weights were equal.

**Logistic Regression:** Standard generalized linear model with logit link[13]:

$$\begin{aligned} \log\frac{p_{i}}{1-p_{i}}=\beta_{0}+\beta^{\top}x_{i}\#\left( 8 \right) \end{aligned}$$

Predictors were centered and scaled, class weights equal, and convergence parameters left at default; no regularization or feature selection was applied.

**References**

1. Yang S, Zhu Z, Yuan Y, Chen S, Shang X, Bulloch G, et al. Analysis of Plasma Metabolic Profile on Ganglion Cell-Inner Plexiform Layer Thickness With Mortality and Common Diseases. JAMA Netw Open. 2023;6(5):e2313220. Epub 2023-5-1. doi: 10.1001/jamanetworkopen.2023.13220. PubMed 37191963.

2. Chen Y, Yuan Y, Zhang S, Yang S, Zhang J, Guo X, et al. Retinal nerve fiber layer thinning as a novel fingerprint for cardiovascular events: results from the prospective cohorts in UK and China. BMC Med. 2023;21(1):24. Epub 2023-1-18. doi: 10.1186/s12916-023-02728-7. PubMed 36653845.

3. Patel PJ, Foster PJ, Grossi CM, Keane PA, Ko F, Lotery A, et al. Spectral-Domain Optical Coherence Tomography Imaging in 67 321 Adults: Associations with Macular Thickness in the UK Biobank Study. Ophthalmology. 2016;123(4):829-40. doi: 10.1016/j.ophtha.2015.11.009. PubMed 26746598.

4. Sun BB, Chiou J, Traylor M, Benner C, Hsu Y, Richardson TG, et al. Plasma proteomic associations with genetics and health in the UK Biobank. Nature. 2023;622(7982):329-338. Epub 2023-10-12. doi: 10.1038/s41586-023-06592-6. PubMed 37794186.

5. Eldjarn GH, Ferkingstad E, Lund SH, Helgason H, Magnusson OT, Gunnarsdottir K, et al. Large-scale plasma proteomics comparisons through genetics and disease associations. Nature. 2023;622(7982):348-358. Epub 2023-10-1. doi: 10.1038/s41586-023-06563-x. PubMed 37794188.

6. Dhindsa RS, Burren OS, Sun BB, Prins BP, Matelska D, Wheeler E, et al. Rare variant associations with plasma protein levels in the UK Biobank. Nature. 2023;622(7982):339-347. Epub 2023-10-1. doi: 10.1038/s41586-023-06547-x. PubMed 37794183.

7. Rhodes JS, Cutler A, Moon KR. Geometry- and Accuracy-Preserving Random Forest Proximities. IEEE Trans Pattern Anal Mach Intell. 2023;45(9):10947-10959. Epub 2023-9-1. doi: 10.1109/TPAMI.2023.3263774. PubMed 37015125.

8. Dou B, Zhu Z, Merkurjev E, Ke L, Chen L, Jiang J, et al. Machine Learning Methods for Small Data Challenges in Molecular Science. Chem Rev. 2023;123(13):8736-8780. doi: 10.1021/acs.chemrev.3c00189. PubMed 37384816.

9. Hu J, Szymczak S. A review on longitudinal data analysis with random forest. Brief Bioinform. 2023;24(2). doi: 10.1093/bib/bbad002. PubMed 36653905.

10. Chang C, Lin C. LIBSVM: A library for support vector machines. ACM Trans Intell Syst Technol. 2011;2(3):Article 27. doi: 10.1145/1961189.1961199. PubMed.

11. LeCun Y, Bengio Y, Hinton G. Deep learning. Nature. 2015;521(7553):436-44. doi: 10.1038/nature14539. PubMed 26017442.

12. Binson VA, Thomas S, Subramoniam M, Arun J, Naveen S, Madhu S. A Review of Machine Learning Algorithms for Biomedical Applications. Ann Biomed Eng. 2024;52(5):1159-1183. doi: 10.1007/s10439-024-03459-3. PubMed 38383870.

13. Fan R, Chang K, Hsieh C, Wang X, Lin C. LIBLINEAR: a library for large linear classification. J Mach Learn Res. 2008;9:1871-1874. Epub 2008-8-1. doi: 10.1145/1390681.1442794. PubMed.
