# Supplementary material for: Plasma proteomic signatures of early retinal neurodegeneration in diabetes: a multi-cohort study
Source: PLoS Med. 2026 Jun 2;23(6):e1004868. doi: 10.1371/journal.pmed.1004868 (PMC13229346; doi:10.1371/journal.pmed.1004868)
Supplement: S1 Table — (DOCX) [file pmed.1004868.s004.docx]

## S1 Table. Baseline characteristics of the population 1 (GDES-PPP cross-sectional cohort) and population 3 (UKB-PPP validation cohort)

| **Characteristics** | **GDES cohort** | **UKB cohort** |
| --- | --- | --- |
| **No. of subjects** | 1492 | 502 |
| **Age, year** | 64.37±7.53 | 58.25±7.64 |
| **Sex** |  |  |
| Female | 854 (57.3%) | 255 (50.8%) |
| Male | 638 (42.7%) | 247 (49.2%) |
| **Educational attainment** |  |  |
| Junior High School and below | 506 (33.9%) | 164 (32.7%) |
| High School | 551 (36.9%) | 22 (4.4%) |
| University and above | 426 (28.6%) | 309 (61.6%) |
| Missing | 9 (0.6%) | 7 (1.4%) |
| **Income** |  |  |
| Low | 68 (4.6%) | 124 (24.7%) |
| Lower-middle | 989 (66.3%) | 222 (44.3%) |
| Upper-middle | 207 (13.9%) | 63 (12.5%) |
| High | 23 (1.5%) | 10 (2.0%) |
| Missing | 205 (13.7%) | 83 (16.5%) |
| **Smoking status** |  |  |
| Ever/Current | 1317 (88.3%) | 244 (48.6%) |
| Never | 175 (11.7%) | 192 (38.2%) |
| Missing | - | 66 (13.1%) |
| **Alcohol** |  |  |
| Ever/Current | 1366 (91.6%) | 39 (7.8%) |
| Never | 126 (8.4%) | 19 (3.8%) |
| Missing | - | 444 (88.4%) |
| **Duration of diabetes, year** | 8.59±6.86 | 3.24±2.31 |
| **Body mass index, kg/m^2^** | 24.54±3.19 | 29.58±5.45 |
| **Lipid-lowering medication** |  |  |
| No | 1458 (97.7%) | 316 (62.9%) |
| Yes | 34 (2.3%) | 186 (37.1%) |
| **Blood pressure-lowering medication** |  |  |
| No | 1421 (95.2%) | 321 (63.9%) |
| Yes | 71 (4.8%) | 181 (36.1%) |
