# Supplementary material for: Plasma proteomic signatures of early retinal neurodegeneration in diabetes: a multi-cohort study
Source: PLoS Med. 2026 Jun 2;23(6):e1004868. doi: 10.1371/journal.pmed.1004868 (PMC13229346; doi:10.1371/journal.pmed.1004868)
Supplement: S2 Table — (DOCX) [file pmed.1004868.s005.docx]

## S2 Table. Proteins associated with average retinal nerve fiber layer thickness

| **Proteins *** | **Panels** | **β †** | **95% CI** | | **P value** | **P_FDR_ value ‡** |
| --- | --- | --- | --- | --- | --- | --- |
| TFF3 | Cardiometabolic | -2.014 | -2.800 | -1.229 | 5.67×10^-07^ | 2.05×10^-04^ |
| MCFD2 | Cardiometabolic | -1.722 | -2.443 | -1.001 | 3.14×10^-06^ | 4.54×10^-04^ |
| CST3 | Cardiometabolic | -1.884 | -2.680 | -1.088 | 3.77×10^-06^ | 4.54×10^-04^ |
| FAM3C | Cardiometabolic | -1.779 | -2.555 | -1.003 | 7.53×10^-06^ | 0.001 |
| CGREF1 | Cardiometabolic | -1.636 | -2.363 | -0.908 | 1.14×10^-05^ | 0.001 |
| NECTIN2 | Cardiometabolic | -1.781 | -2.575 | -0.987 | 1.18×10^-05^ | 0.001 |
| NPDC1 | Cardiometabolic | -1.812 | -2.623 | -1.001 | 1.27×10^-05^ | 0.001 |
| CLC | Cardiometabolic | -1.571 | -2.288 | -0.854 | 1.88×10^-05^ | 0.001 |
| HSPG2 | Cardiometabolic | -1.752 | -2.563 | -0.941 | 2.45×10^-05^ | 0.001 |
| DEFA1 | Cardiometabolic | -1.592 | -2.335 | -0.849 | 2.85×10^-05^ | 0.001 |
| CCL14 | Cardiometabolic | -1.561 | -2.296 | -0.825 | 3.39×10^-05^ | 0.001 |
| PI3 | Cardiometabolic | -1.655 | -2.460 | -0.850 | 5.89×10^-05^ | 0.002 |
| IGFBP2 | Cardiometabolic | -1.569 | -2.341 | -0.798 | 7.02×10^-05^ | 0.002 |
| EPHB4 | Cardiometabolic | -1.573 | -2.351 | -0.795 | 7.78×10^-05^ | 0.002 |
| COL6A3 | Cardiometabolic | -1.618 | -2.420 | -0.815 | 8.14×10^-05^ | 0.002 |
| IGFBP6 | Cardiometabolic | -1.680 | -2.521 | -0.840 | 9.28×10^-05^ | 0.002 |
| CD46 | Cardiometabolic | -1.505 | -2.274 | -0.736 | 1.31×10^-04^ | 0.003 |
| CD59 | Cardiometabolic | -1.586 | -2.400 | -0.772 | 1.40×10^-04^ | 0.003 |
| ACTA2 | Cardiometabolic | -1.595 | -2.417 | -0.773 | 1.48×10^-04^ | 0.003 |
| CLEC1A | Cardiometabolic | -1.492 | -2.263 | -0.721 | 1.55×10^-04^ | 0.003 |
| RETN | Cardiometabolic | -1.349 | -2.049 | -0.649 | 1.56×10^-04^ | 0.003 |
| RARRES2 | Cardiometabolic | -1.393 | -2.117 | -0.669 | 1.69×10^-04^ | 0.003 |
| LCN2 | Cardiometabolic | -1.438 | -2.190 | -0.687 | 1.82×10^-04^ | 0.003 |
| PTGDS | Cardiometabolic | -1.541 | -2.351 | -0.731 | 2.00×10^-04^ | 0.003 |
| CD14 | Cardiometabolic | -1.364 | -2.107 | -0.620 | 3.34×10^-04^ | 0.005 |
| TINAGL1 | Cardiometabolic | -1.313 | -2.041 | -0.585 | 4.22×10^-04^ | 0.006 |
| RNASET2 | Cardiometabolic | -1.380 | -2.147 | -0.613 | 4.37×10^-04^ | 0.006 |
| REG1A | Cardiometabolic | -1.312 | -2.051 | -0.573 | 0.001 | 0.007 |
| PLIN3 | Cardiometabolic | -1.234 | -1.941 | -0.528 | 0.001 | 0.008 |
| REG3A | Cardiometabolic | -1.286 | -2.024 | -0.548 | 0.001 | 0.008 |
| PAM | Cardiometabolic | -1.269 | -2.001 | -0.537 | 0.001 | 0.008 |
| EFEMP1 | Cardiometabolic | -1.400 | -2.212 | -0.588 | 0.001 | 0.008 |
| NOTCH3 | Cardiometabolic | -1.324 | -2.104 | -0.544 | 0.001 | 0.010 |
| CTSL | Cardiometabolic | -1.227 | -1.961 | -0.494 | 0.001 | 0.011 |
| GRK5 | Cardiometabolic | -1.139 | -1.829 | -0.449 | 0.001 | 0.013 |
| CDH1 | Cardiometabolic | -1.224 | -1.969 | -0.479 | 0.001 | 0.013 |
| GPR37 | Cardiometabolic | -1.231 | -1.984 | -0.479 | 0.001 | 0.013 |
| CTSZ | Cardiometabolic | -1.210 | -1.953 | -0.466 | 0.001 | 0.014 |
| PRSS2 | Cardiometabolic | -1.137 | -1.849 | -0.425 | 0.002 | 0.016 |
| SPON2 | Cardiometabolic | -1.206 | -1.968 | -0.444 | 0.002 | 0.017 |
| ANGPTL1 | Cardiometabolic | -1.135 | -1.853 | -0.417 | 0.002 | 0.017 |
| CCL15 | Cardiometabolic | -1.135 | -1.855 | -0.415 | 0.002 | 0.017 |
| PDGFRA | Cardiometabolic | -1.130 | -1.850 | -0.410 | 0.002 | 0.018 |
| DKK3 | Cardiometabolic | -1.163 | -1.906 | -0.421 | 0.002 | 0.018 |
| ESAM | Cardiometabolic | -1.203 | -1.983 | -0.423 | 0.003 | 0.020 |
| NT-proBNP | Cardiometabolic | -1.222 | -2.016 | -0.428 | 0.003 | 0.020 |
| CCN3 | Cardiometabolic | -1.238 | -2.044 | -0.431 | 0.003 | 0.020 |
| REG1B | Cardiometabolic | -1.106 | -1.828 | -0.384 | 0.003 | 0.020 |
| LGALS1 | Cardiometabolic | -1.155 | -1.910 | -0.401 | 0.003 | 0.020 |
| TNF | Cardiometabolic | -1.095 | -1.820 | -0.371 | 0.003 | 0.022 |
| MFAP5 | Cardiometabolic | -1.159 | -1.930 | -0.388 | 0.003 | 0.023 |
| ART3 | Cardiometabolic | -1.202 | -2.001 | -0.402 | 0.003 | 0.023 |
| CXCL8 | Cardiometabolic | -1.042 | -1.746 | -0.339 | 0.004 | 0.025 |
| ROR1 | Cardiometabolic | -1.132 | -1.898 | -0.366 | 0.004 | 0.025 |
| CA4 | Cardiometabolic | -1.071 | -1.797 | -0.344 | 0.004 | 0.026 |
| COL18A1 | Cardiometabolic | -1.108 | -1.865 | -0.350 | 0.004 | 0.027 |
| XG | Cardiometabolic | -1.272 | -2.148 | -0.397 | 0.004 | 0.028 |
| CCL16 | Cardiometabolic | -1.048 | -1.776 | -0.321 | 0.005 | 0.030 |
| PPP1R2 | Cardiometabolic | -1.002 | -1.701 | -0.302 | 0.005 | 0.030 |
| IL19 | Cardiometabolic | -1.066 | -1.810 | -0.322 | 0.005 | 0.030 |
| CD93 | Cardiometabolic | -1.096 | -1.868 | -0.325 | 0.005 | 0.031 |
| GDF15 | Cardiometabolic | -1.076 | -1.833 | -0.318 | 0.005 | 0.031 |
| SCARF1 | Cardiometabolic | -1.039 | -1.770 | -0.307 | 0.005 | 0.031 |
| PRTN3 | Cardiometabolic | -0.990 | -1.687 | -0.292 | 0.005 | 0.031 |
| VCAM1 | Cardiometabolic | -1.026 | -1.762 | -0.29 | 0.006 | 0.035 |
| CLEC5A | Cardiometabolic | -1.038 | -1.787 | -0.288 | 0.007 | 0.037 |
| CCL27 | Cardiometabolic | -1.071 | -1.848 | -0.294 | 0.007 | 0.037 |
| SEMA3F | Cardiometabolic | -0.983 | -1.702 | -0.265 | 0.007 | 0.039 |
| UMOD | Cardiometabolic | 1.019 | 0.273 | 1.764 | 0.007 | 0.039 |
| TIMP1 | Cardiometabolic | -1.031 | -1.790 | -0.271 | 0.008 | 0.041 |
| IL2RA | Cardiometabolic | -1.001 | -1.745 | -0.257 | 0.008 | 0.043 |
| THBD | Cardiometabolic | -1.037 | -1.808 | -0.266 | 0.008 | 0.043 |

* Adjusted for age, sex, smoking, systolic blood pressure, HbA1c and duration of diabetes.

† Per-SD change of retinal nerve fiber layer thickness.

‡ Adjusted for multiple testing (Benjamini-Hochberg procedure).

CI = confidence interval.
