# Supplementary material for: Plasma proteomic signatures of early retinal neurodegeneration in diabetes: a multi-cohort study
Source: PLoS Med. 2026 Jun 2;23(6):e1004868. doi: 10.1371/journal.pmed.1004868 (PMC13229346; doi:10.1371/journal.pmed.1004868)
Supplement: S3 Table — (DOCX) [file pmed.1004868.s006.docx]

## S3 Table. Proteins associated with the thinning rate of retinal nerve fiber layer thickness

| **Proteins *** | **Panels** | **β †** | **95% CI** | | **P value** | **P_FDR_ value ‡** |
| --- | --- | --- | --- | --- | --- | --- |
| CST3 | Cardiometabolic | -0.432 | -0.502 | -0.362 | 9.86×10^-32^ | 7.10×10^-30^ |
| HSPG2 | Cardiometabolic | -0.445 | -0.518 | -0.372 | 3.82×10^-31^ | 1.38×10^-29^ |
| NECTIN2 | Cardiometabolic | -0.398 | -0.466 | -0.330 | 4.17×10^-29^ | 1.00×10^-27^ |
| COL6A3 | Cardiometabolic | -0.410 | -0.480 | -0.339 | 1.46×10^-28^ | 2.63×10^-27^ |
| ACTA2 | Cardiometabolic | -0.391 | -0.459 | -0.322 | 9.09×10^-28^ | 1.31×10^-26^ |
| NPDC1 | Cardiometabolic | -0.426 | -0.501 | -0.351 | 3.30×10^-27^ | 3.96×10^-26^ |
| CD59 | Cardiometabolic | -0.419 | -0.494 | -0.345 | 4.81×10^-27^ | 4.94×10^-26^ |
| PTGDS | Cardiometabolic | -0.397 | -0.468 | -0.325 | 2.79×10^-26^ | 2.51×10^-25^ |
| RNASET2 | Cardiometabolic | -0.349 | -0.413 | -0.286 | 5.05×10^-26^ | 4.04×10^-25^ |
| FAM3C | Cardiometabolic | -0.366 | -0.433 | -0.300 | 7.79×10^-26^ | 5.61×10^-25^ |
| CD46 | Cardiometabolic | -0.351 | -0.416 | -0.287 | 1.19×10^-25^ | 7.77×10^-25^ |
| COL18A1 | Cardiometabolic | -0.332 | -0.396 | -0.268 | 4.64×10^-23^ | 2.78×10^-22^ |
| EFEMP1 | Cardiometabolic | -0.343 | -0.411 | -0.275 | 2.26×10^-22^ | 1.25×10^-21^ |
| IGFBP6 | Cardiometabolic | -0.372 | -0.446 | -0.298 | 3.31×10^-22^ | 1.70×10^-21^ |
| CTSZ | Cardiometabolic | -0.290 | -0.352 | -0.229 | 6.85×10^-20^ | 3.29×10^-19^ |
| CLEC1A | Cardiometabolic | -0.313 | -0.380 | -0.247 | 1.24×10^-19^ | 5.57×10^-19^ |
| SPON2 | Cardiometabolic | -0.302 | -0.366 | -0.238 | 1.37×10^-19^ | 5.78×10^-19^ |
| TIMP1 | Cardiometabolic | -0.291 | -0.353 | -0.229 | 2.20×10^-19^ | 8.80×10^-19^ |
| GDF15 | Cardiometabolic | -0.295 | -0.358 | -0.232 | 2.55×10^-19^ | 9.66×10^-19^ |
| ESAM | Cardiometabolic | -0.309 | -0.375 | -0.243 | 2.89×10^-19^ | 1.04×10^-18^ |
| RARRES2 | Cardiometabolic | -0.279 | -0.340 | -0.218 | 1.49×10^-18^ | 5.10×10^-18^ |
| THBD | Cardiometabolic | -0.289 | -0.355 | -0.223 | 2.83×10^-17^ | 9.25×10^-17^ |
| ROR1 | Cardiometabolic | -0.280 | -0.345 | -0.215 | 7.73×10^-17^ | 2.42×10^-16^ |
| EPHB4 | Cardiometabolic | -0.282 | -0.349 | -0.216 | 1.42×10^-16^ | 4.25×10^-16^ |
| MCFD2 | Cardiometabolic | -0.254 | -0.314 | -0.195 | 1.87×10^-16^ | 5.39×10^-16^ |
| CCL14 | Cardiometabolic | -0.263 | -0.325 | -0.201 | 3.17×10^-16^ | 8.79×10^-16^ |
| TFF3 | Cardiometabolic | -0.284 | -0.352 | -0.216 | 9.71×10^-16^ | 2.59×10^-15^ |
| CD93 | Cardiometabolic | -0.268 | -0.333 | -0.204 | 1.03×10^-15^ | 2.64×10^-15^ |
| PAM | Cardiometabolic | -0.253 | -0.314 | -0.192 | 1.57×10^-15^ | 3.89×10^-15^ |
| LGALS1 | Cardiometabolic | -0.268 | -0.333 | -0.203 | 1.72×10^-15^ | 4.12×10^-15^ |
| SCARF1 | Cardiometabolic | -0.254 | -0.316 | -0.192 | 3.30×10^-15^ | 7.67×10^-15^ |
| DEFA1 | Cardiometabolic | -0.261 | -0.326 | -0.197 | 4.08×10^-15^ | 9.17×10^-15^ |
| UMOD | Cardiometabolic | 0.256 | 0.192 | 0.320 | 6.84×10^-15^ | 1.49×10^-14^ |
| CLEC5A | Cardiometabolic | -0.252 | -0.317 | -0.188 | 3.15×10^-14^ | 6.67×10^-14^ |
| XG | Cardiometabolic | -0.296 | -0.374 | -0.219 | 1.04×10^-13^ | 2.14×10^-13^ |
| MFAP5 | Cardiometabolic | -0.248 | -0.314 | -0.182 | 2.62×10^-13^ | 5.23×10^-13^ |
| CDH1 | Cardiometabolic | -0.231 | -0.293 | -0.169 | 4.46×10^-13^ | 8.68×10^-13^ |
| CCN3 | Cardiometabolic | -0.265 | -0.336 | -0.193 | 6.21×10^-13^ | 1.18×10^-12^ |
| CD14 | Cardiometabolic | -0.228 | -0.290 | -0.167 | 7.13×10^-13^ | 1.32×10^-12^ |
| REG1A | Cardiometabolic | -0.232 | -0.296 | -0.169 | 1.18×10^-12^ | 2.13×10^-12^ |
| CA4 | Cardiometabolic | -0.218 | -0.277 | -0.158 | 1.37×10^-12^ | 2.41×10^-12^ |
| CCL15 | Cardiometabolic | -0.214 | -0.274 | -0.154 | 3.85×10^-12^ | 6.60×10^-12^ |
| CGREF1 | Cardiometabolic | -0.220 | -0.282 | -0.158 | 4.60×10^-12^ | 7.70×10^-12^ |
| IL2RA | Cardiometabolic | -0.217 | -0.279 | -0.155 | 1.03×10^-11^ | 1.68×10^-11^ |
| SEMA3F | Cardiometabolic | -0.206 | -0.266 | -0.145 | 3.29×10^-11^ | 5.26×10^-11^ |
| PRSS2 | Cardiometabolic | -0.205 | -0.266 | -0.145 | 4.24×10^-11^ | 6.64×10^-11^ |
| LCN2 | Cardiometabolic | -0.220 | -0.285 | -0.155 | 4.77×10^-11^ | 7.30×10^-11^ |
| GPR37 | Cardiometabolic | -0.214 | -0.278 | -0.151 | 6.01×10^-11^ | 9.01×10^-11^ |
| ART3 | Cardiometabolic | -0.231 | -0.300 | -0.161 | 1.02×10^-10^ | 1.49×10^-10^ |
| PDGFRA | Cardiometabolic | -0.196 | -0.257 | -0.136 | 3.13×10^-10^ | 4.51×10^-10^ |
| VCAM1 | Cardiometabolic | -0.194 | -0.255 | -0.134 | 4.77×10^-10^ | 6.73×10^-10^ |
| REG1B | Cardiometabolic | -0.193 | -0.254 | -0.132 | 6.45×10^-10^ | 8.93×10^-10^ |
| TNF | Cardiometabolic | -0.191 | -0.252 | -0.130 | 9.43×10^-10^ | 1.28×10^-09^ |
| PLIN3 | Cardiometabolic | -0.181 | -0.239 | -0.122 | 2.45×10^-09^ | 3.26×10^-09^ |
| IGFBP2 | Cardiometabolic | -0.196 | -0.261 | -0.132 | 3.35×10^-09^ | 4.38×10^-09^ |
| ANGPTL1 | Cardiometabolic | -0.180 | -0.239 | -0.120 | 3.64×10^-09^ | 4.68×10^-09^ |
| CCL27 | Cardiometabolic | -0.189 | -0.253 | -0.124 | 1.48×10^-08^ | 1.87×10^-08^ |
| CTSL | Cardiometabolic | -0.169 | -0.229 | -0.109 | 4.28×10^-08^ | 5.32×10^-08^ |
| DKK3 | Cardiometabolic | -0.167 | -0.228 | -0.105 | 1.52×10^-07^ | 1.85×10^-07^ |
| REG3A | Cardiometabolic | -0.167 | -0.231 | -0.104 | 2.75×10^-07^ | 3.30×10^-07^ |
| CCL16 | Cardiometabolic | -0.157 | -0.217 | -0.097 | 3.68×10^-07^ | 4.34×10^-07^ |
| PI3 | Cardiometabolic | -0.178 | -0.246 | -0.109 | 3.81×10^-07^ | 4.42×10^-07^ |
| NT-proBNP | Cardiometabolic | -0.158 | -0.224 | -0.092 | 3.12×10^-06^ | 3.57×10^-06^ |
| RETN | Cardiometabolic | -0.132 | -0.191 | -0.073 | 1.42×10^-05^ | 1.59×10^-05^ |
| CXCL8 | Cardiometabolic | -0.120 | -0.177 | -0.063 | 3.85×10^-05^ | 4.26×10^-05^ |
| PPP1R2 | Cardiometabolic | -0.123 | -0.182 | -0.064 | 4.79×10^-05^ | 5.23×10^-05^ |
| CLC | Cardiometabolic | -0.124 | -0.184 | -0.064 | 5.45×10^-05^ | 5.85×10^-05^ |
| PRTN3 | Cardiometabolic | -0.12 | -0.178 | -0.061 | 6.55×10^-05^ | 6.93×10^-05^ |
| TINAGL1 | Cardiometabolic | -0.124 | -0.186 | -0.063 | 7.21×10^-05^ | 7.52×10^-05^ |
| NOTCH3 | Cardiometabolic | -0.121 | -0.186 | -0.057 | 2.37×10^-04^ | 2.44×10^-04^ |
| IL19 | Cardiometabolic | -0.112 | -0.173 | -0.050 | 3.69×10^-04^ | 3.74×10^-04^ |

* Adjusted for age, sex, smoking, systolic blood pressure, HbA1c and duration of diabetes.

† Per-SD change of retinal nerve fiber layer thickness.

‡ Adjusted for multiple testing (Benjamini-Hochberg procedure).

CI = confidence interval.
