# Supplementary material for: Plasma proteomic signatures of early retinal neurodegeneration in diabetes: a multi-cohort study
Source: PLoS Med. 2026 Jun 2;23(6):e1004868. doi: 10.1371/journal.pmed.1004868 (PMC13229346; doi:10.1371/journal.pmed.1004868)
Supplement: S4 Table — (DOCX) [file pmed.1004868.s007.docx]

## S4 Table. Proteins associated with the thinning rate of retinal nerve fiber layer thickness in the sensitivity analysis with further adjustment for baseline retinal nerve fiber layer thickness

| **Proteins *** | **Panels** | **β †** | **95% CI** | | **P value** | **P_FDR_ value ‡** |
| --- | --- | --- | --- | --- | --- | --- |
| CST3 | Cardiometabolic | -0.420 | -0.487 | -0.353 | 1.38×10^-32^ | 9.92×10^-31^ |
| HSPG2 | Cardiometabolic | -0.429 | -0.499 | -0.359 | 1.33×10^-31^ | 4.77×10^-30^ |
| NECTIN2 | Cardiometabolic | -0.391 | -0.456 | -0.326 | 1.65×10^-30^ | 3.97×10^-29^ |
| COL6A3 | Cardiometabolic | -0.399 | -0.466 | -0.331 | 1.52×10^-29^ | 2.73×10^-28^ |
| ACTA2 | Cardiometabolic | -0.381 | -0.447 | -0.316 | 7.68×10^-29^ | 1.11×10^-27^ |
| NPDC1 | Cardiometabolic | -0.417 | -0.489 | -0.345 | 1.89×10^-28^ | 2.26×10^-27^ |
| CD59 | Cardiometabolic | -0.406 | -0.477 | -0.335 | 1.10×10^-27^ | 1.13×10^-26^ |
| PTGDS | Cardiometabolic | -0.390 | -0.458 | -0.321 | 1.39×10^-27^ | 1.25×10^-26^ |
| CD46 | Cardiometabolic | -0.348 | -0.410 | -0.287 | 2.10×10^-27^ | 1.68×10^-26^ |
| RNASET2 | Cardiometabolic | -0.341 | -0.402 | -0.281 | 4.23×10^-27^ | 3.04×10^-26^ |
| FAM3C | Cardiometabolic | -0.358 | -0.422 | -0.294 | 7.52×10^-24^ | 4.92×10^-26^ |
| COL18A1 | Cardiometabolic | -0.323 | -0.384 | -0.261 | 7.78×10^-24^ | 4.67×10^-23^ |
| EFEMP1 | Cardiometabolic | -0.338 | -0.402 | -0.273 | 1.03×10^-23^ | 5.71×10^-23^ |
| IGFBP6 | Cardiometabolic | -0.358 | -0.429 | -0.287 | 2.08×10^-22^ | 1.07×10^-21^ |
| ESAM | Cardiometabolic | -0.315 | -0.378 | -0.251 | 1.02×10^-21^ | 4.88×10^-21^ |
| SPON2 | Cardiometabolic | -0.297 | -0.358 | -0.235 | 1.40×10^-20^ | 6.30×10^-20^ |
| CLEC1A | Cardiometabolic | -0.306 | -0.370 | -0.243 | 1.74×10^-20^ | 7.08×10^-20^ |
| RARRES2 | Cardiometabolic | -0.281 | -0.340 | -0.223 | 1.77×10^-20^ | 7.08×10^-20^ |
| CTSZ | Cardiometabolic | -0.279 | -0.337 | -0.220 | 5.12×10^-20^ | 1.94×10^-19^ |
| TIMP1 | Cardiometabolic | -0.283 | -0.342 | -0.223 | 5.80×10^-20^ | 2.09×10^-19^ |
| GDF15 | Cardiometabolic | -0.284 | -0.344 | -0.223 | 1.68×10^-19^ | 5.76×10^-19^ |
| EPHB4 | Cardiometabolic | -0.280 | -0.343 | -0.216 | 1.24×10^-17^ | 4.05×10^-17^ |
| SCARF1 | Cardiometabolic | -0.262 | -0.321 | -0.203 | 1.67×10^-17^ | 5.21×10^-17^ |
| MCFD2 | Cardiometabolic | -0.251 | -0.308 | -0.194 | 2.10×10^-17^ | 6.30×10^-17^ |
| THBD | Cardiometabolic | -0.277 | -0.340 | -0.214 | 2.38×10^-17^ | 6.86×10^-17^ |
| ROR1 | Cardiometabolic | -0.271 | -0.334 | -0.209 | 3.12×10^-17^ | 8.63×10^-17^ |
| PAM | Cardiometabolic | -0.252 | -0.311 | -0.194 | 9.15×10^-17^ | 2.44×10^-16^ |
| CCL14 | Cardiometabolic | -0.256 | -0.316 | -0.197 | 9.63×10^-17^ | 2.48×10^-16^ |
| CD93 | Cardiometabolic | -0.265 | -0.327 | -0.204 | 1.11×10^-16^ | 2.76×10^-16^ |
| LGALS1 | Cardiometabolic | -0.263 | -0.325 | -0.200 | 3.47×10^-16^ | 8.32×10^-16^ |
| TFF3 | Cardiometabolic | -0.276 | -0.341 | -0.210 | 4.28×10^-16^ | 9.95×10^-16^ |
| DEFA1 | Cardiometabolic | -0.255 | -0.316 | -0.193 | 1.30×10^-15^ | 2.93×10^-15^ |
| CLEC5A | Cardiometabolic | -0.249 | -0.310 | -0.187 | 4.98×10^-14^ | 1.09×10^-14^ |
| UMOD | Cardiometabolic | 0.241 | 0.181 | 0.302 | 1.61×10^-14^ | 3.40×10^-14^ |
| XG | Cardiometabolic | -0.292 | -0.366 | -0.218 | 1.84×10^-14^ | 3.78×10^-14^ |
| MFAP5 | Cardiometabolic | -0.245 | -0.308 | -0.182 | 4.10×10^-14^ | 8.20×10^-14^ |
| CDH1 | Cardiometabolic | -0.227 | -0.287 | -0.168 | 9.67×10^-14^ | 1.88×10^-13^ |
| CGREF1 | Cardiometabolic | -0.225 | -0.284 | -0.166 | 1.53×10^-13^ | 2.90×10^-13^ |
| CCN3 | Cardiometabolic | -0.259 | -0.327 | -0.190 | 1.92×10^-13^ | 3.55×10^-13^ |
| REG1A | Cardiometabolic | -0.227 | -0.288 | -0.166 | 3.98×10^-13^ | 7.16×10^-13^ |
| CD14 | Cardiometabolic | -0.220 | -0.279 | -0.161 | 4.40×10^-13^ | 7.72×10^-13^ |
| CA4 | Cardiometabolic | -0.207 | -0.264 | -0.150 | 2.10×10^-12^ | 3.60×10^-12^ |
| CCL15 | Cardiometabolic | -0.206 | -0.264 | -0.149 | 2.60×10^-12^ | 4.36×10^-12^ |
| IL2RA | Cardiometabolic | -0.212 | -0.271 | -0.152 | 4.22×10^-12^ | 6.91×10^-12^ |
| PRSS2 | Cardiometabolic | -0.203 | -0.260 | -0.145 | 9.26×10^-12^ | 1.48×10^-11^ |
| LCN2 | Cardiometabolic | -0.212 | -0.274 | -0.150 | 3.00×10^-11^ | 4.70×10^-11^ |
| ART3 | Cardiometabolic | -0.227 | -0.294 | -0.160 | 3.51×10^-11^ | 5.38×10^-11^ |
| GPR37 | Cardiometabolic | -0.207 | -0.268 | -0.146 | 3.99×10^-11^ | 5.98×10^-11^ |
| SEMA3F | Cardiometabolic | -0.195 | -0.253 | -0.138 | 4.36×10^-11^ | 6.41×10^-11^ |
| PLIN3 | Cardiometabolic | -0.188 | -0.245 | -0.132 | 7.52×10^-11^ | 1.08×10^-10^ |
| TNF | Cardiometabolic | -0.192 | -0.250 | -0.134 | 1.13×10^-10^ | 1.60×10^-10^ |
| REG1B | Cardiometabolic | -0.192 | -0.250 | -0.134 | 1.32×10^-10^ | 1.83×10^-10^ |
| PDGFRA | Cardiometabolic | -0.192 | -0.250 | -0.134 | 1.41×10^-10^ | 1.91×10^-10^ |
| VCAM1 | Cardiometabolic | -0.188 | -0.246 | -0.130 | 3.06×10^-10^ | 4.08×10^-10^ |
| IGFBP2 | Cardiometabolic | -0.198 | -0.260 | -0.136 | 4.89×10^-10^ | 6.40×10^-10^ |
| ANGPTL1 | Cardiometabolic | -0.168 | -0.224 | -0.111 | 8.92×10^-09^ | 1.15×10^-08^ |
| CCL27 | Cardiometabolic | -0.182 | -0.244 | -0.120 | 1.06×10^-08^ | 1.34×10^-08^ |
| REG3A | Cardiometabolic | -0.170 | -0.231 | -0.109 | 4.95×10^-08^ | 6.14×10^-08^ |
| CTSL | Cardiometabolic | -0.159 | -0.217 | -0.102 | 7.15×10^-08^ | 8.73×10^-08^ |
| DKK3 | Cardiometabolic | -0.162 | -0.221 | -0.103 | 1.07×10^-07^ | 1.26×10^-07^ |
| PI3 | Cardiometabolic | -0.177 | -0.242 | -0.112 | 1.32×10^-07^ | 1.55×10^-07^ |
| CCL16 | Cardiometabolic | -0.154 | -0.212 | -0.097 | 1.80×10^-07^ | 2.09×10^-07^ |
| NT-proBNP | Cardiometabolic | -0.163 | -0.226 | -0.099 | 5.54×10^-07^ | 6.34×10^-07^ |
| PPP1R2 | Cardiometabolic | -0.135 | -0.191 | -0.078 | 3.15×10^-06^ | 3.54×10^-06^ |
| CXCL8 | Cardiometabolic | -0.129 | -0.183 | -0.074 | 4.15×10^-06^ | 4.60×10^-06^ |
| RETN | Cardiometabolic | -0.131 | -0.187 | -0.074 | 7.08×10^-06^ | 7.73×10^-06^ |
| CLC | Cardiometabolic | -0.121 | -0.179 | -0.063 | 4.15×10^-05^ | 4.46×10^-05^ |
| PRTN3 | Cardiometabolic | -0.113 | -0.169 | -0.057 | 8.61×10^-05^ | 9.12×10^-05^ |
| TINAGL1 | Cardiometabolic | -0.116 | -0.174 | -0.057 | 1.17×10^-04^ | 1.22×10^-04^ |
| NOTCH3 | Cardiometabolic | -0.121 | -0.183 | -0.059 | 1.34×10^-04^ | 1.38×10^-04^ |
| IL19 | Cardiometabolic | -0.110 | -0.168 | -0.051 | 2.65×10^-04^ | 2.69×10^-04^ |

* Adjusted for age, sex, smoking, systolic blood pressure, HbA1c, duration of diabetes and baseline retinal nerve fiber layer thickness.

† Per-SD change of retinal nerve fiber layer thickness.

‡ Adjusted for multiple testing (Benjamini-Hochberg procedure).

CI = confidence interval.
