# Supplementary material for: Plasma proteomic signatures of early retinal neurodegeneration in diabetes: a multi-cohort study
Source: PLoS Med. 2026 Jun 2;23(6):e1004868. doi: 10.1371/journal.pmed.1004868 (PMC13229346; doi:10.1371/journal.pmed.1004868)
Supplement: S5 Table — (DOCX) [file pmed.1004868.s008.docx]

## S5 Table. Performance of Pro-DRN for predicting DRN with various ML algorithms in the GDES cohort

| **ML algorithms** | **Train dataset** | | | **Test dataset** | | |
| --- | --- | --- | --- | --- | --- | --- |
|  | **C-index** | **95% CI** | | **C-index** | **95% CI** | |
| XGboost | 0.979 | 0.972 | 0.986 | 0.860 | 0.810 | 0.911 |
| LightGBM | 0.919 | 0.902 | 0.936 | 0.832 | 0.777 | 0.886 |
| Random forest | 0.984 | 0.977 | 0.990 | 0.816 | 0.756 | 0.876 |
| Neural network​​ | 0.932 | 0.916 | 0.948 | 0.792 | 0.728 | 0.857 |
| Logistic regression | 0.760 | 0.726 | 0.795 | 0.787 | 0.718 | 0.857 |
| ​K-Nearest Neighbors​ | 0.917 | 0.899 | 0.934 | 0.773 | 0.708 | 0.838 |
| Support vector machine | 0.947 | 0.933 | 0.961 | 0.782 | 0.719 | 0.845 |
| Decision tree | 0.841 | 0.814 | 0.869 | 0.736 | 0.667 | 0.805 |

Pro-DRN = Proteome-deciphering diabetic retinal neurodegeneration; CI = confidence interval​.
