# Supplementary material for: Plasma proteomic signatures of early retinal neurodegeneration in diabetes: a multi-cohort study
Source: PLoS Med. 2026 Jun 2;23(6):e1004868. doi: 10.1371/journal.pmed.1004868 (PMC13229346; doi:10.1371/journal.pmed.1004868)
Supplement: S6 Table — (DOCX) [file pmed.1004868.s009.docx]

**S6 Table.** Comparison of the performance between conventional predictors and Pro-DRN for predicting DRN

|  | **C-index (95% CI)** | | **P value^*^** |
| --- | --- | --- | --- |
|  | **Conventional** | **Pro-DRN** |  |
| Age | 0.688 (0.615, 0.760) | 0.860 (0.810, 0.911) | <0.001 |
| Sex | 0.557 (0.485, 0.630) | 0.860 (0.810, 0.911) | <0.001 |
| Income | 0.531 (0.463, 0.599) | 0.860 (0.810, 0.911) | <0.001 |
| Smoking | 0.517 (0.465, 0.570) | 0.860 (0.810, 0.911) | <0.001 |
| Drinking | 0.528 (0.477, 0.580) | 0.860 (0.810, 0.911) | <0.001 |
| Educational attainment | 0.552 (0.475, 0.629) | 0.860 (0.810, 0.911) | <0.001 |
| Body mass index | 0.568 (0.495, 0.640) | 0.860 (0.810, 0.911) | <0.001 |
| Lipid-lowering medication | 0.505 (0.481, 0.530) | 0.860 (0.810, 0.911) | <0.001 |
| Antihypertensive medication | 0.500 (0.475, 0.526) | 0.860 (0.810, 0.911) | <0.001 |

Pro-DRN = Proteome-deciphering diabetic retinal neurodegeneration; CI = confidence interval.

* P values were calculated using DeLong’s test for paired comparisons of C-indices between each conventional predictor and Pro-DRN.
