# Supplementary material for: Plasma proteomic signatures of early retinal neurodegeneration in diabetes: a multi-cohort study
Source: PLoS Med. 2026 Jun 2;23(6):e1004868. doi: 10.1371/journal.pmed.1004868 (PMC13229346; doi:10.1371/journal.pmed.1004868)
Supplement: S7 Table — (DOCX) [file pmed.1004868.s010.docx]

**S7 Table.** Comparison of the performance of incorporating Pro-DRN with the full proteins in the GDES

| **Outcomes** | **C-index (95% CI)** | | **Performance**  **gaps** | **Percentages** |
| --- | --- | --- | --- | --- |
|  | **Incorporating full** **proteins** | **Incorporating Pro-DRN** |  |  |
| **XGboost model** |  |  |  |  |
| Age&Sex | 0.855 (0.808, 0.903) | 0.877 (0.834, 0.921) | 0.022 | 2.55% |
| Aspelund model | 0.871 (0.828, 0.915) | 0.896 (0.852, 0.941) | 0.017 | 1.95% |
| Hippisley model | 0.880 (0.838, 0.923) | 0.905 (0.863, 0.946) | 0.017 | 1.98% |
| Dagliati model | 0.882 (0.840, 0.924) | 0.902 (0.859, 0.945) | 0.016 | 1.83% |
| ISDR model | 0.876 (0.833, 0.918) | 0.897 (0.853, 0.941) | 0.013 | 1.47% |
| JDC model | 0.872 (0.828, 0.916) | 0.896 (0.851, 0.940) | 0.017 | 1.94% |
| Tarasewicz model | 0.881 (0.838, 0.924) | 0.903 (0.862, 0.944) | 0.017 | 1.89% |
| All model | 0.903 (0.865, 0.941) | 0.919 (0.882, 0.957) | 0.005 | 0.58% |
| **LightGBM model** |  |  |  |  |
| Age&Sex | 0.833 (0.778, 0.888) | 0.848 (0.799, 0.897) | 0.015 | 1.84% |
| Aspelund model | 0.849 (0.800, 0.899) | 0.876 (0.829, 0.924) | 0.011 | 1.26% |
| Hippisley model | 0.856 (0.805, 0.907) | 0.885 (0.838, 0.932) | 0.019 | 2.16% |
| Dagliati model | 0.856 (0.808, 0.904) | 0.887 (0.840, 0.933) | 0.017 | 1.99% |
| ISDR model | 0.850 (0.801, 0.899) | 0.875 (0.827, 0.923) | 0.011 | 1.31% |
| JDC model | 0.851 (0.801, 0.901) | 0.876 (0.829, 0.924) | 0.011 | 1.24% |
| Tarasewicz model | 0.861 (0.811, 0.910) | 0.888 (0.842, 0.934) | 0.012 | 1.42% |
| All model | 0.876 (0.832, 0.921) | 0.904 (0.863, 0.945) | 0.015 | 1.69% |
| **RF model** |  |  |  |  |
| Age&Sex | 0.833 (0.775, 0.891) | 0.844 (0.791, 0.897) | 0.011 | 1.28% |
| Aspelund model | 0.855 (0.805, 0.906) | 0.886 (0.839, 0.934) | 0.013 | 1.51% |
| Hippisley model | 0.860 (0.807, 0.912) | 0.885 (0.835, 0.935) | 0.012 | 1.43% |
| Dagliati model | 0.864 (0.815, 0.913) | 0.894 (0.849, 0.940) | 0.017 | 1.92% |
| ISDR model | 0.854 (0.803, 0.906) | 0.886 (0.840, 0.933) | 0.014 | 1.63% |
| JDC model | 0.859 (0.808, 0.909) | 0.885 (0.837, 0.933) | 0.009 | 1.07% |
| Tarasewicz model | 0.863 (0.812, 0.914) | 0.888 (0.840, 0.937) | 0.012 | 1.38% |
| All model | 0.888 (0.846, 0.931) | 0.917 (0.878, 0.956) | 0.008 | 0.94% |
| **NN model** |  |  |  |  |
| Age&Sex | 0.794 (0.735, 0.854) | 0.814 (0.757, 0.871) | 0.019 | 2.43% |
| Aspelund model | 0.808 (0.751, 0.864) | 0.857 (0.806, 0.907) | 0.027 | 3.40% |
| Hippisley model | 0.815 (0.758, 0.871) | 0.860 (0.806, 0.913) | 0.027 | 3.37% |
| Dagliati model | 0.816 (0.760, 0.871) | 0.873 (0.825, 0.922) | 0.033 | 4.03% |
| ISDR model | 0.811 (0.756, 0.866) | 0.859 (0.808, 0.909) | 0.027 | 3.39% |
| JDC model | 0.817 (0.759, 0.874) | 0.862 (0.811, 0.912) | 0.025 | 3.10% |
| Tarasewicz model | 0.825 (0.770, 0.880) | 0.874 (0.825, 0.924) | 0.029 | 3.50% |
| All model | 0.850 (0.799, 0.901) | 0.888 (0.842, 0.935) | 0.020 | 2.37% |
| **Logistic model** |  |  |  |  |
| Age&Sex | 0.722 (0.652, 0.791) | 0.824 (0.767, 0.881) | 0.102 | 14.18% |
| Aspelund model | 0.744 (0.678, 0.810) | 0.858 (0.804, 0.912) | 0.097 | 13.04% |
| Hippisley model | 0.755 (0.688, 0.822) | 0.862 (0.808, 0.916) | 0.094 | 12.39% |
| Dagliati model | 0.755 (0.689, 0.821) | 0.871 (0.819, 0.923) | 0.099 | 13.06% |
| ISDR model | 0.748 (0.682, 0.814) | 0.860 (0.807, 0.913) | 0.097 | 12.91% |
| JDC model | 0.762 (0.696, 0.828) | 0.863 (0.809, 0.917) | 0.084 | 11.04% |
| Tarasewicz model | 0.779 (0.715, 0.843) | 0.876 (0.825, 0.927) | 0.080 | 10.32% |
| All model | 0.823 (0.764, 0.882) | 0.893 (0.846, 0.940) | 0.052 | 6.32% |
| **KNN model** |  |  |  |  |
| Age&Sex | 0.785 (0.722, 0.849) | 0.816 (0.760, 0.873) | 0.031 | 3.93% |
| Aspelund model | 0.805 (0.748, 0.862) | 0.860 (0.811, 0.909) | 0.036 | 4.46% |
| Hippisley model | 0.817 (0.759, 0.875) | 0.869 (0.820, 0.918) | 0.035 | 4.24% |
| Dagliati model | 0.815 (0.760, 0.870) | 0.873 (0.826, 0.921) | 0.036 | 4.40% |
| ISDR model | 0.805 (0.748, 0.862) | 0.860 (0.811, 0.909) | 0.035 | 4.29% |
| JDC model | 0.816 (0.759, 0.872) | 0.861 (0.812, 0.911) | 0.026 | 3.25% |
| Tarasewicz model | 0.819 (0.762, 0.876) | 0.874 (0.826, 0.922) | 0.033 | 4.04% |
| All model | 0.849 (0.800, 0.899) | 0.897 (0.855, 0.939) | 0.025 | 2.98% |
| **SVM model** |  |  |  |  |
| Age&Sex | 0.814 (0.756, 0.871) | 0.810 (0.755, 0.866) | -0.004 | -0.44% |
| Aspelund model | 0.829 (0.776, 0.882) | 0.848 (0.796, 0.900) | -0.003 | -0.40% |
| Hippisley model | 0.840 (0.787, 0.894) | 0.855 (0.803, 0.907) | -0.002 | -0.24% |
| Dagliati model | 0.839 (0.787, 0.891) | 0.860 (0.810, 0.911) | -0.005 | -0.55% |
| ISDR model | 0.832 (0.780, 0.884) | 0.850 (0.798, 0.902) | -0.004 | -0.48% |
| JDC model | 0.834 (0.781, 0.888) | 0.850 (0.798, 0.903) | -0.008 | -0.98% |
| Tarasewicz model | 0.842 (0.789, 0.896) | 0.862 (0.811, 0.913) | -0.001 | -0.10% |
| All model | 0.869 (0.822, 0.916) | 0.884 (0.835, 0.933) | -0.004 | -0.44% |
| **DT model** |  |  |  |  |
| Age&Sex | 0.744 (0.679, 0.810) | 0.760 (0.697, 0.824) | 0.016 | 2.12% |
| Aspelund model | 0.778 (0.717, 0.838) | 0.821 (0.765, 0.877) | 0.017 | 2.23% |
| Hippisley model | 0.783 (0.721, 0.845) | 0.814 (0.753, 0.875) | 0.010 | 1.26% |
| Dagliati model | 0.779 (0.717, 0.840) | 0.836 (0.783, 0.890) | 0.024 | 3.13% |
| ISDR model | 0.775 (0.714, 0.836) | 0.822 (0.766, 0.879) | 0.021 | 2.65% |
| JDC model | 0.786 (0.724, 0.848) | 0.834 (0.777, 0.890) | 0.019 | 2.42% |
| Tarasewicz model | 0.791 (0.730, 0.851) | 0.843 (0.788, 0.898) | 0.030 | 3.76% |
| All model | 0.827 (0.770, 0.884) | 0.872 (0.822, 0.922) | 0.018 | 2.21% |

Pro-DRN = Proteome-based diabetic retinal neurodegeneration; CI = confidence interval; RF = Random forest; NN = Neural network; KNN = ​K-Nearest Neighbors​; SVM = ​​Support vector machine​; DT = Decision tree.
