# Supplementary material for: Plasma proteomic signatures of early retinal neurodegeneration in diabetes: a multi-cohort study
Source: PLoS Med. 2026 Jun 2;23(6):e1004868. doi: 10.1371/journal.pmed.1004868 (PMC13229346; doi:10.1371/journal.pmed.1004868)
Supplement: S8 Table — (DOCX) [file pmed.1004868.s011.docx]

**S8 Table.** Incremental predictive value of Pro-DRN beyond metabolically augmented conventional clinical models

| **Outcomes** | **C-index (95% CI)** | | **Improvement** | **P value^*^** |
| --- | --- | --- | --- | --- |
|  | **Benchmark** | **Incorporating Pro-DRN** |  |  |
| **XGboost model** |  |  |  |  |
| Age&Sex | 0.697 (0.626, 0.768) | 0.877 (0.834, 0.921) | 25.87% | **4.20×10^-07^** |
| Aspelund model | 0.779 (0.714, 0.843) | 0.896 (0.852, 0.941) | 15.14% | **2.46×10^-05^** |
| Hippisley model | 0.774 (0.704, 0.843) | 0.905 (0.863, 0.946) | 16.89% | **2.22×10^-05^** |
| Dagliati model | 0.795 (0.733, 0.858) | 0.902 (0.859, 0.945) | 13.39% | **8.17×10^-05^** |
| ISDR model | 0.779 (0.715, 0.844) | 0.897 (0.853, 0.941) | 15.08% | **2.95×10^-05^** |
| JDC model | 0.802 (0.739, 0.865) | 0.896 (0.851, 0.940) | 11.69% | **3.12×10^-04^** |
| Tarasewicz model | 0.809 (0.746, 0.873) | 0.903 (0.862, 0.944) | 11.58% | **4.53×10^-04^** |
| All model | 0.843 (0.783, 0.903) | 0.919 (0.882, 0.957) | 9.01% | **0.002** |
| **LightGBM model** |  |  |  |  |
| Age&Sex | 0.697 (0.626, 0.768) | 0.848 (0.799, 0.897) | 21.67% | **1.32×10^-05^** |
| Aspelund model | 0.779 (0.714, 0.843) | 0.876 (0.829, 0.924) | 12.54% | **2.02×10^-04^** |
| Hippisley model | 0.774 (0.704, 0.843) | 0.885 (0.838, 0.932) | 14.37% | **2.69×10^-05^** |
| Dagliati model | 0.795 (0.733, 0.858) | 0.887 (0.840, 0.933) | 11.48% | **5.04×10^-05^** |
| ISDR model | 0.779 (0.715, 0.844) | 0.875 (0.827, 0.923) | 12.31% | **2.97×10^-04^** |
| JDC model | 0.802 (0.739, 0.865) | 0.876 (0.829, 0.924) | 9.3% | **0.003** |
| Tarasewicz model | 0.809 (0.746, 0.873) | 0.888 (0.842, 0.934) | 9.72% | **0.002** |
| All model | 0.843 (0.783, 0.903) | 0.904 (0.863, 0.945) | 7.27% | **0.003** |
| **RF model** |  |  |  |  |
| Age&Sex | 0.697 (0.626, 0.768) | 0.844 (0.791, 0.897) | 21.06% | **4.06×10^-05^** |
| Aspelund model | 0.779 (0.714, 0.843) | 0.886 (0.839, 0.934) | 13.83% | **1.60×10^-04^** |
| Hippisley model | 0.774 (0.704, 0.843) | 0.885 (0.835, 0.935) | 14.36% | **3.54×10^-04^** |
| Dagliati model | 0.795 (0.733, 0.858) | 0.894 (0.849, 0.940) | 12.44% | **3.84×10^-04^** |
| ISDR model | 0.779 (0.715, 0.844) | 0.886 (0.840, 0.933) | 13.77% | **1.96×10^-04^** |
| JDC model | 0.802 (0.739, 0.865) | 0.885 (0.837, 0.933) | 10.35% | **0.002** |
| Tarasewicz model | 0.809 (0.746, 0.873) | 0.888 (0.840, 0.937) | 9.75% | **0.004** |
| All model | 0.843 (0.783, 0.903) | 0.917 (0.878, 0.956) | 8.76% | **0.002** |
| **NN model** |  |  |  |  |
| Age&Sex | 0.697 (0.626, 0.768) | 0.814 (0.757, 0.871) | 16.75% | **6.99×10^-04^** |
| Aspelund model | 0.779 (0.714, 0.843) | 0.857 (0.806, 0.907) | 10% | **0.002** |
| Hippisley model | 0.774 (0.704, 0.843) | 0.860 (0.806, 0.913) | 11.09% | **0.001** |
| Dagliati model | 0.795 (0.733, 0.858) | 0.873 (0.825, 0.922) | 9.75% | **0.002** |
| ISDR model | 0.779 (0.715, 0.844) | 0.859 (0.808, 0.909) | 10.24% | **0.002** |
| JDC model | 0.802 (0.739, 0.865) | 0.862 (0.811, 0.912) | 7.48% | **0.007** |
| Tarasewicz model | 0.809 (0.746, 0.873) | 0.874 (0.825, 0.924) | 8.05% | **0.004** |
| All model | 0.843 (0.783, 0.903) | 0.888 (0.842, 0.935) | 5.38% | **0.016** |
| **Logistic model** |  |  |  |  |
| Age&Sex | 0.697 (0.626, 0.768) | 0.824 (0.767, 0.881) | 18.21% | **3.51×10^-04^** |
| Aspelund model | 0.779 (0.714, 0.843) | 0.858 (0.804, 0.912) | 10.19% | **7.62×10^-04^** |
| Hippisley model | 0.774 (0.704, 0.843) | 0.862 (0.808, 0.916) | 11.44% | **8.54×10^-04^** |
| Dagliati model | 0.795 (0.733, 0.858) | 0.871 (0.819, 0.923) | 9.49% | **0.001** |
| ISDR model | 0.779 (0.715, 0.844) | 0.860 (0.807, 0.913) | 10.4% | **8.34×10^-04^** |
| JDC model | 0.802 (0.739, 0.865) | 0.863 (0.809, 0.917) | 7.65% | **0.004** |
| Tarasewicz model | 0.809 (0.746, 0.873) | 0.876 (0.825, 0.927) | 8.23% | **0.004** |
| All model | 0.843 (0.783, 0.903) | 0.893 (0.846, 0.940) | 5.92% | **0.007** |
| **KNN model** |  |  |  |  |
| Age&Sex | 0.697 (0.626, 0.768) | 0.816 (0.760, 0.873) | 17.12% | **3.74×10^-04^** |
| Aspelund model | 0.779 (0.714, 0.843) | 0.860 (0.811, 0.909) | 10.48% | **0.001** |
| Hippisley model | 0.774 (0.704, 0.843) | 0.869 (0.820, 0.918) | 12.24% | **7.33×10^-04^** |
| Dagliati model | 0.795 (0.733, 0.858) | 0.873 (0.826, 0.921) | 9.76% | **0.002** |
| ISDR model | 0.779 (0.715, 0.844) | 0.860 (0.811, 0.909) | 10.42% | **0.001** |
| JDC model | 0.802 (0.739, 0.865) | 0.861 (0.812, 0.911) | 7.45% | **0.010** |
| Tarasewicz model | 0.809 (0.746, 0.873) | 0.874 (0.826, 0.922) | 7.98% | **0.006** |
| All model | 0.843 (0.783, 0.903) | 0.897 (0.855, 0.939) | 6.41% | **0.007** |
| **SVM model** |  |  |  |  |
| Age&Sex | 0.697 (0.626, 0.768) | 0.810 (0.755, 0.866) | 16.26% | **6.73×10^-04^** |
| Aspelund model | 0.779 (0.714, 0.843) | 0.848 (0.796, 0.900) | 8.96% | **0.001** |
| Hippisley model | 0.774 (0.704, 0.843) | 0.855 (0.803, 0.907) | 10.52% | **0.001** |
| Dagliati model | 0.795 (0.733, 0.858) | 0.860 (0.810, 0.911) | 8.16% | **0.004** |
| ISDR model | 0.779 (0.715, 0.844) | 0.850 (0.798, 0.902) | 9.08% | **0.001** |
| JDC model | 0.802 (0.739, 0.865) | 0.850 (0.798, 0.903) | 6.05% | **0.011** |
| Tarasewicz model | 0.809 (0.746, 0.873) | 0.862 (0.811, 0.913) | 6.47% | **0.011** |
| All model | 0.843 (0.783, 0.903) | 0.884 (0.835, 0.933) | 4.82% | **0.011** |
| **DT model** |  |  |  |  |
| Age&Sex | 0.697 (0.626, 0.768) | 0.760 (0.697, 0.824) | 9.09% | **0.012** |
| Aspelund model | 0.779 (0.714, 0.843) | 0.821 (0.765, 0.877) | 5.45% | **0.016** |
| Hippisley model | 0.774 (0.704, 0.843) | 0.814 (0.753, 0.875) | 5.21% | **0.033** |
| Dagliati model | 0.795 (0.733, 0.858) | 0.836 (0.783, 0.890) | 5.16% | **0.018** |
| ISDR model | 0.779 (0.715, 0.844) | 0.822 (0.766, 0.879) | 5.55% | **0.015** |
| JDC model | 0.802 (0.739, 0.865) | 0.834 (0.777, 0.890) | 3.99% | **0.040** |
| Tarasewicz model | 0.809 (0.746, 0.873) | 0.843 (0.788, 0.898) | 4.16% | **0.026** |
| All model | 0.843 (0.783, 0.903) | 0.872 (0.822, 0.922) | 3.46% | **0.057** |

**Note:** Metabolically augmented baseline models were created by adding additional pathophysiologic measures to each conventional clinical framework (predictor sets in S13 Table): fasting insulin, HOMA2-IR and TyG index (insulin resistance); fasting C-peptide and HOMA2-B (β-cell function); and fasting glucose plus HbA1c (glycemic indices; HbA1c was retained when already included in the original model).

Pro-DRN = Proteome-based diabetic retinal neurodegeneration; CI = confidence interval; RF = Random forest; NN = Neural network; KNN = ​K-Nearest Neighbors​; SVM = ​​Support vector machine​; DT = Decision tree.

***** P values were calculated using DeLong’s test for paired comparisons of C-indices between the benchmark model and the corresponding model incorporating Pro-DRN.
