# Supplementary material for: Plasma proteomic signatures of early retinal neurodegeneration in diabetes: a multi-cohort study
Source: PLoS Med. 2026 Jun 2;23(6):e1004868. doi: 10.1371/journal.pmed.1004868 (PMC13229346; doi:10.1371/journal.pmed.1004868)
Supplement: S9 Table — (DOCX) [file pmed.1004868.s012.docx]

## S9 Table. Discrimination of Pro-DRN under alternative DRN definitions

| **DRN outcome definition** | **Train dataset** | | | **Test dataset** | | |
| --- | --- | --- | --- | --- | --- | --- |
|  | **C-index** | **95% CI** | | **C-index** | **95% CI** | |
| DRN-Top10Slope | 0.995 | 0.991 | 0.999 | 0.880 | 0.819 | 0.941 |
| DRN-ThinLast | 0.876 | 0.850 | 0.903 | 0.753 | 0.672 | 0.834 |
| DRN-ExcessLoss | 0.959 | 0.948 | 0.970 | 0.848 | 0.794 | 0.903 |

DRN-Top10Slope indicates the fastest 10% of annualized RNFL thinners. DRN-ThinLast indicates thin/abnormal pRNFL on the OCT report at the final available visit according to the device’s age-matched normative database. DRN-ExcessLoss indicates an annualized RNFL thinning rate below the lower reference limit (mean − 1.96 SD) derived from the distribution of longitudinal RNFL slopes in healthy COIP participants (see Supplementary Methods). Pro-DRN = Proteome-deciphering diabetic retinal neurodegeneration; CI = confidence interval​.
