# Supplementary material for: Plasma proteomic signatures of early retinal neurodegeneration in diabetes: a multi-cohort study
Source: PLoS Med. 2026 Jun 2;23(6):e1004868. doi: 10.1371/journal.pmed.1004868 (PMC13229346; doi:10.1371/journal.pmed.1004868)
Supplement: S10 Table — (DOCX) [file pmed.1004868.s013.docx]

## S10 Table. Incremental discrimination of Pro-DRN beyond conventional clinical frameworks under alternative DRN definitions

| **Outcomes** | **C-index (95% CI)** | | **Improvement** | **P value^*^** |
| --- | --- | --- | --- | --- |
|  | **Benchmark** | **Incorporating Pro-DRN** |  |  |
| **DRN-Top10Slope** |  |  |  |  |
| Age&Sex | 0.712 (0.618, 0.807) | 0.895 (0.849, 0.941) | 25.64% | **2.31×10^-04^** |
| Aspelund model | 0.718 (0.624, 0.811) | 0.898 (0.849, 0.947) | 25.09% | **3.65×10^-04^** |
| Hippisley model | 0.744 (0.655, 0.833) | 0.922 (0.886, 0.957) | 23.86% | **1.18×10^-04^** |
| Dagliati model | 0.767 (0.684, 0.851) | 0.923 (0.888, 0.958) | 20.30% | **2.06×10^-04^** |
| ISDR model | 0.732 (0.641, 0.824) | 0.900 (0.849, 0.951) | 22.98% | **0.001** |
| JDC model | 0.807 (0.722, 0.892) | 0.919 (0.878, 0.961) | 13.91% | **0.006** |
| Tarasewicz model | 0.814 (0.732, 0.896) | 0.932 (0.895, 0.968) | 14.41% | **0.003** |
| All model | 0.856 (0.774, 0.938) | 0.935 (0.898, 0.971) | 9.17% | **0.023** |
| **DRN-ThinLast** |  |  |  |  |
| Age&Sex | 0.558 (0.445, 0.672) | 0.762 (0.685, 0.804) | 36.47% | **4.04×10^-04^** |
| Aspelund model | 0.570 (0.461, 0.679) | 0.764 (0.687, 0.842) | 34.06% | **0.001** |
| Hippisley model | 0.668 (0.572, 0.764) | 0.795 (0.723, 0.868) | 19.02% | **0.004** |
| Dagliati model | 0.650 (0.555, 0.745) | 0.771 (0.692, 0.850) | 18.62% | **0.005** |
| ISDR model | 0.587 (0.474, 0.700) | 0.773 (0.697, 0.849) | 31.72% | **0.001** |
| JDC model | 0.614 (0.507, 0.721) | 0.769 (0.690, 0.847) | 25.18% | **0.005** |
| Tarasewicz model | 0.672 (0.578, 0.766) | 0.782 (0.707, 0.857) | 16.42% | **0.006** |
| All model | 0.738 (0.654, 0.822) | 0.832 (0.766, 0.897) | 12.68% | **0.007** |
| **DRN-ExcessLoss** |  |  |  |  |
| Age&Sex | 0.703 (0.627, 0.779) | 0.857 (0.805, 0.909) | 21.90% | **5.30×10^-05^** |
| Aspelund model | 0.713 (0.638, 0.787) | 0.864 (0.813, 0.914) | 21.21% | **8.32×10^-05^** |
| Hippisley model | 0.754 (0.686, 0.823) | 0.892 (0.848, 0.936) | 18.21% | **6.26×10^-05^** |
| Dagliati model | 0.748 (0.678, 0.818) | 0.889 (0.843, 0.936) | 18.93% | **3.56×10^-05^** |
| ISDR model | 0.714 (0.641, 0.788) | 0.863 (0.809, 0.916) | 20.79% | **9.18×10^-05^** |
| JDC model | 0.747 (0.670, 0.824) | 0.860 (0.807, 0.914) | 15.19% | **5.84×10^-04^** |
| Tarasewicz model | 0.785 (0.716, 0.854) | 0.891 (0.846, 0.936) | 13.45% | **6.88×10^-04^** |
| All model | 0.823 (0.754, 0.891) | 0.902 (0.859, 0.945) | 9.61% | **0.005** |

DRN-Top10Slope indicates the fastest 10% of annualized RNFL thinners. DRN-ThinLast indicates thin/abnormal pRNFL on the OCT report at the final available visit according to the device’s age-matched normative database. DRN-ExcessLoss indicates an annualized RNFL thinning rate below the lower reference limit (mean − 1.96 SD) derived from the distribution of longitudinal RNFL slopes in healthy COIP participants (see Supplementary Methods). Pro-DRN = Proteome-deciphering diabetic retinal neurodegeneration; CI = confidence interval​.

***** P values were calculated using DeLong’s test for paired comparisons of C-indices between the benchmark model and the corresponding model incorporating Pro-DRN.
