# Supplementary material for: Plasma proteomic signatures of early retinal neurodegeneration in diabetes: a multi-cohort study
Source: PLoS Med. 2026 Jun 2;23(6):e1004868. doi: 10.1371/journal.pmed.1004868 (PMC13229346; doi:10.1371/journal.pmed.1004868)
Supplement: S11 Table — (DOCX) [file pmed.1004868.s014.docx]

## S11 Table. Model-level external validation of Pro-DRN in UKB-PPP using a cross-sectional thin-RNFL outcome

|  | **C-index (95% CI)** | | **Improvement** | **P value^*^** |
| --- | --- | --- | --- | --- |
|  | **Benchmark** | **Incorporating Pro-DRN** |  |  |
| Age&Sex | 0.635 (0.579, 0.692) | 0.714 (0.661, 0.766) | 12.29% | **0.002** |
| Aspelund model | 0.642 (0.586, 0.698) | 0.724 (0.672, 0.777) | 12.88% | **0.001** |
| Hippisley model | 0.657 (0.603, 0.712) | 0.736 (0.684, 0.787) | 11.91% | **0.001** |
| Dagliati model | 0.664 (0.608, 0.719) | 0.735 (0.685, 0.786) | 10.76% | **0.001** |
| ISDR model | 0.644 (0.593, 0.698) | 0.721 (0.669, 0.773) | 11.86% | **0.001** |
| JDC model | 0.615 (0.560, 0.669) | 0.722 (0.670, 0.774) | 17.43% | **8.26×10^-05^** |
| Tarasewicz model | 0.649 (0.595, 0.704) | 0.739 (0.689, 0.790) | 13.90% | **1.95×10^-04^** |
| All model | 0.676 (0.622, 0.730) | 0.756 (0.706, 0.806) | 11.84% | **3.23×10^-04^** |

Pro-DRN = Proteome-deciphering diabetic retinal neurodegeneration; CI = confidence interval​.

***** P values were calculated using DeLong’s test for paired comparisons of C-indices between the benchmark model and the corresponding model incorporating Pro-DRN.
