# Supplementary material for: Plasma proteomic signatures of early retinal neurodegeneration in diabetes: a multi-cohort study
Source: PLoS Med. 2026 Jun 2;23(6):e1004868. doi: 10.1371/journal.pmed.1004868 (PMC13229346; doi:10.1371/journal.pmed.1004868)
Supplement: S12 Table — (DOCX) [file pmed.1004868.s015.docx]

## S12 Table. Assessment of model calibration for Pro-DRN in UKB-PPP using a cross-sectional thin-RNFL outcome

|  | **Benchmark** | | **Incorporating Pro-DRN** | |
| --- | --- | --- | --- | --- |
|  | **Brier Score** | **Hosmer-Lemeshow**  **Test** | **Brier Score** | **Hosmer-Lemeshow**  **Test** |
| Age&Sex | 0.181 | P = 0.821 | 0.166 | P = 0.411 |
| Aspelund model | 0.180 | P = 0.272 | 0.164 | P = 0.730 |
| Hippisley model | 0.178 | P = 0.638 | 0.162 | P = 0.275 |
| Dagliati model | 0.177 | P = 0.450 | 0.163 | P = 0.343 |
| ISDR model | 0.148 | P = 0.639 | 0.132 | P = 0.966 |
| JDC model | 0.183 | P = 0.631 | 0.165 | P = 0.194 |
| Tarasewicz model | 0.179 | P = 0.782 | 0.161 | P = 0.181 |
| All model | 0.175 | P = 0.357 | 0.157 | P = 0.665 |

Pro-DRN = Proteome-deciphering diabetic retinal neurodegeneration; CI = confidence interval​.
