# Supplementary material for: Plasma proteomic signatures of early retinal neurodegeneration in diabetes: a multi-cohort study
Source: PLoS Med. 2026 Jun 2;23(6):e1004868. doi: 10.1371/journal.pmed.1004868 (PMC13229346; doi:10.1371/journal.pmed.1004868)
Supplement: S13 Table — (DOCX) [file pmed.1004868.s016.docx]

## S13 Table. Predictors used in conventional prediction models

| **Models** | **Variates** |
| --- | --- |
| Age & Sex | Age, sex |
| Aspelund model [1] | Sex, systolic blood pressure, duration of diabetes and HbA1c |
| Hippisley model [2] | Sex, body mass index, systolic blood pressure, TC/HDL and HbA1c |
| Dagliati model [3] | Age, sex, duration of diabetes, body mass index, HbA1c, smoking status and hypertension |
| ISDR model [4] | Age, sex, systolic blood pressure, HbA1c, total cholesterol, and duration of diabetes |
| JDC model [5] | Age, systolic blood pressure, HbA1c, eGFR, and duration of diabetes |
| Tarasewicz model [6] | Age, body mass index, systolic blood pressure, HbA1c, LDL-c, creatinine, MAU and insulin use |

eGFR = estimated glomerular filtration rate.

**References**

1. Aspelund T, Thornorisdottir O, Olafsdottir E, Gudmundsdottir A, Einarsdottir AB, Mehlsen J, et al. Individual risk assessment and information technology to optimise screening frequency for diabetic retinopathy. Diabetologia. 2011;54(10):2525-32. Epub 2011-10-1. doi: 10.1007/s00125-011-2257-7. PubMed 21792613.

2. Hippisley-Cox J, Coupland C. Development and validation of risk prediction equations to estimate future risk of blindness and lower limb amputation in patients with diabetes: cohort study. BMJ. 2015;351:h5441. Epub 2015-11-11. doi: 10.1136/bmj.h5441. PubMed 26560308.

3. Dagliati A, Marini S, Sacchi L, Cogni G, Teliti M, Tibollo V, et al. Machine Learning Methods to Predict Diabetes Complications. J Diabetes Sci Technol. 2018;12(2):295-302. Epub 2018-3-1. doi: 10.1177/1932296817706375. PubMed 28494618.

4. Eleuteri A, Fisher AC, Broadbent DM, Garcia-Finana M, Cheyne CP, Wang A, et al. Individualised variable-interval risk-based screening for sight-threatening diabetic retinopathy: the Liverpool Risk Calculation Engine. Diabetologia. 2017;60(11):2174-2182. Epub 2017-11-1. doi: 10.1007/s00125-017-4386-0. PubMed 28840258.

5. Tanaka S, Tanaka S, Iimuro S, Yamashita H, Katayama S, Akanuma Y, et al. Predicting macro- and microvascular complications in type 2 diabetes: the Japan Diabetes Complications Study/the Japanese Elderly Diabetes Intervention Trial risk engine. Diabetes Care. 2013;36(5):1193-9. Epub 2013-5-1. doi: 10.2337/dc12-0958. PubMed 23404305.

6. Tarasewicz D, Karter AJ, Pimentel N, Moffet HH, Thai KK, Schlessinger D, et al. Development and Validation of a Diabetic Retinopathy Risk Stratification Algorithm. Diabetes Care. 2023;Epub 2023-3-17. doi: 10.2337/dc22-1168. PubMed 36930723.
