# Supplementary material for: Plasma proteomic signatures of early retinal neurodegeneration in diabetes: a multi-cohort study
Source: PLoS Med. 2026 Jun 2;23(6):e1004868. doi: 10.1371/journal.pmed.1004868 (PMC13229346; doi:10.1371/journal.pmed.1004868)
Supplement: S14 Table — (DOCX) [file pmed.1004868.s017.docx]

## S14 Table. Protein names and gene names for proteins identified in this study.

| **Proteins** | **Gene name** |
| --- | --- |
| TFF3 | Trefoil factor 3 |
| MCFD2 | Multiple coagulation factor deficiency protein 2 |
| CST3 | Cystatin-C |
| FAM3C | Protein FAM3C |
| CGREF1 | Cell growth regulator with EF hand domain protein 1 |
| NECTIN2 | Nectin-2 |
| NPDC1 | Neural proliferation differentiation and control protein 1 |
| CLC | Galectin-10 |
| HSPG2 | Basement membrane heparan sulfate proteoglycan core protein |
| DEFA1 | Neutrophil defensin 1 |
| CCL14 | C-C motif chemokine 14 |
| PI3 | Elafin |
| IGFBP2 | Insulin-like growth factor-binding protein 2 |
| EPHB4 | Ephrin type-B receptor 4 |
| COL6A3 | Collagen alpha-3(VI) chain |
| IGFBP6 | Insulin-like growth factor-binding protein 6 |
| CD46 | Membrane cofactor protein |
| CD59 | CD59 glycoprotein |
| ACTA2 | Actin, aortic smooth muscle |
| CLEC1A | C-type lectin domain family 1 member A |
| RETN | Resistin |
| RARRES2 | Retinoic acid receptor responder protein 2 |
| LCN2 | Neutrophil gelatinase-associated lipocalin |
| PTGDS | Prostaglandin-H2 D-isomerase |
| CD14 | Monocyte differentiation antigen CD14 |
| TINAGL1 | Tubulointerstitial nephritis antigen-like |
| RNASET2 | Ribonuclease T2 |
| REG1A | Lithostathine-1-alpha |
| PLIN3 | Perilipin-3 |
| REG3A | Regenerating islet-derived protein 3-alpha |
| PAM | Peptidyl-glycine alpha-amidating monooxygenase |
| EFEMP1 | EGF-containing fibulin-like extracellular matrix protein 1 |
| NOTCH3 | Neurogenic locus notch homolog protein 3 |
| CTSL | Cathepsin L1 |
| GRK5 | G protein-coupled receptor kinase 5 |
| CDH1 | Cadherin-1 |
| GPR37 | Prosaposin receptor GPR37 |
| CTSZ | Cathepsin Z |
| PRSS2 | Trypsin-2 |
| SPON2 | Spondin-2 |
| ANGPTL1 | Angiopoietin-related protein 1 |
| CCL15 | C-C motif chemokine 15 |
| PDGFRA | Platelet-derived growth factor receptor alpha |
| DKK3 | Dickkopf-related protein 3 |
| ESAM | Endothelial cell-selective adhesion molecule |
| NT-proBNP | N-terminal prohormone of brain natriuretic peptide |
| CCN3 | CCN family member 3 |
| REG1B | Lithostathine-1-beta |
| LGALS1 | Galectin-1 |
| TNF | Tumor necrosis factor |
| MFAP5 | Microfibrillar-associated protein 5 |
| ART3 | Ecto-ADP-ribosyltransferase 3 |
| CXCL8 | Interleukin-8 |
| ROR1 | Inactive tyrosine-protein kinase transmembrane receptor ROR1 |
| CA4 | Carbonic anhydrase 4 |
| COL18A1 | Collagen alpha-1(XVIII) chain |
| XG | Glycoprotein Xg |
| CCL16 | C-C motif chemokine 16 |
| PPP1R2 | Protein phosphatase inhibitor 2 |
| IL19 | Interleukin-19 |
| CD93 | Complement component C1q receptor |
| GDF15 | Growth/differentiation factor 15 |
| SCARF1 | Scavenger receptor class F member 1 |
| PRTN3 | Myeloblastin |
| VCAM1 | Vascular cell adhesion protein 1 |
| CLEC5A | C-type lectin domain family 5 member A |
| CCL27 | C-C motif chemokine 27 |
| SEMA3F | Semaphorin-3F |
| UMOD | Uromodulin |
| TIMP1 | Metalloproteinase inhibitor 1 |
| IL2RA | Interleukin-2 receptor subunit alpha |
| THBD | Thrombomodulin |
