# Supplementary material for: Correction: Plasma proteomic signatures of early retinal neurodegeneration in diabetes: a multi-cohort study
Source: PLoS Med. 2026 Jul 16;23(7):e1005180. doi: 10.1371/journal.pmed.1005180 (PMC13374916; doi:10.1371/journal.pmed.1005180)
Supplement: S1 File — (PDF) [file pmed.1005180.s001.pdf]

PLOS Medicine

RESEARCH ARTICLE

Proteomic signatures of early retinal neurodegeneration in type 2 diabetes mellitus

Huangdong Li[1,2‡], **Ziyu Zhu**[1,2‡], **Shaopeng Yang**[1,2‡], **Weijing Cheng**[1,2], **Shaoying Tan**[3,4,5], **Zhuoyao Xin**[6], **Lei Zhang**[7,8,9], **Zhuoting Zhu**[10], **Shida Chen**[1,2], **Wenyong Huang**[1,2*], **Wei Wang**[1,2,11*]

1 State Key Laboratory of Ophthalmology, Zhongshan Ophthalmic Center, Sun Yat-Sen University, Guangdong Provincial Key Laboratory of Ophthalmology and Visual Science, Guangdong Provincial Clinical Research Center for Ocular Diseases, Guangzhou, China, **2** Guangdong Basic Research Center of Excellence for Major Blinding Eye Diseases Prevention and Treatment, Guangzhou, China, **3** School of Optometry, The Hong Kong Polytechnic University, Kowloon, Hong Kong SAR, China, **4** Research Centre for SHARP Vision (RCSV), The Hong Kong Polytechnic University, Hong Kong, Hong Kong SAR, China, **5** Centre for Eye and Vision Research (CEVR), Hong Kong, Hong Kong SAR, China, **6** Department of Biomedical Engineering, Columbia University, New York, New York, United States of America, **7** Clinical Medical Research Center, Children's Hospital of Nanjing Medical University, Nanjing, Jiangsu Province, China, **8** Artificial Intelligence and Modelling in Epidemiology Program, Melbourne Sexual Health Centre, Alfred Health, Melbourne, Australia, **9** Central Clinical School, Faculty of Medicine, Nursing and Health Sciences, Monash University, Melbourne, Australia, **10** Centre for Eye Research Australia, Royal Victorian Eye and Ear Hospital, Melbourne, Australia, **11** Hainan Eye Hospital and Key Laboratory of Ophthalmology, Zhongshan Ophthalmic Center, Sun Yat-sen University, Haikou, China

‡ These authors are co-first authors on this work.
* huangwenyong@gzzoc.com (WH); wangwei@gzzoc.com (WW)

OPEN ACCESS

Citation: Li H, Zhu Z, Yang S, Cheng W, Tan S, Xin Z, et al. (2026) Proteomic signatures of early retinal neurodegeneration in type 2 diabetes mellitus. PLoS Med 23(6): e1004868. https://doi.org/10.1371/journal.pmed.1004868

Academic Editor: Weiping Jia, Shanghai Jiao Tong University Affiliated Sixth People's Hospital, CHINA

Received: November 30, 2025

Accepted: April 20, 2026

Published: June 2, 2026

Copyright: © 2026 Li et al. This is an open access article distributed under the terms of the Creative Commons Attribution License, which permits unrestricted use, distribution, and reproduction in any medium, provided the original author and source are credited.

Data availability statement: Data from the Guangzhou Diabetic Eye Study (GDES) are subject to Chinese data privacy regulations and are not publicly available. The data are managed by the Preventive Ophthalmology Data Management Unit, Zhongshan Ophthalmic Center, Sun Yat-sen University. Requests for

Abstract

Background

Retinal neurodegeneration is an early and independent feature of diabetic retinal disease and has been proposed as a window into the systemic neural consequences of diabetes, yet accessible molecular biomarkers and individualized prediction tools remain scarce. We aimed to identify circulating plasma protein signatures of diabetic retinal neurodegeneration (DRN) and to translate them into a clinically usable risk prediction system.

Methods and findings

In this multi-cohort prospective observational study, we integrated high-throughput plasma proteomics with longitudinal optical coherence tomography (OCT) in two independent populations. The discovery cohort comprised 1,492 participants had baseline plasma proteomics and OCT, and 1,218 were followed with repeated OCT over 6 years in Guangzhou Diabetic Eye Study (GDES). DRN was quantified by the annualized OCT-derived retinal nerve fiber layer thinning rate. In multivariable analyses adjusted for age, sex, smoking, systolic blood pressure, HbA1c, and diabetes duration, we identified 71 plasma proteins associated with development and

progression of DRN. These proteins mapped onto pathways governing inflammatory immune recruitment, extracellular matrix remodeling, and microvascular homeostasis, providing a plausible biological basis for DRN. We developed a proteomics-based DRN model (Pro-DRN) using eight machine learning (ML) algorithms, including XGBoost and LightGBM. In the independent test set, Pro-DRN achieved a C-index of 0.860, rising to 0.908 when integrated with clinical variables. Compared with six conventional models, Pro-DRN improved discrimination (ΔC-index 0.137 to 0.159; all $P < 0.001$), reclassification (IDI 0.212 to 0.245; NRI 0.226 to 0.452; all $P < 0.05$). In the Hippisley model, the C-index increased from 0.739 (95% CI [0.670, 0.808]) to 0.898 (95% CI [0.858, 0.937]), with IDI 0.245 (95% CI [0.177, 0.318]), NRI 0.452 (95% CI [0.222, 0.673]) (both $P < 0.001$), and higher net benefit. The proteins most consistently driving model performance included ACTA2, COL6A3, and HSPG2. For clinical translation, we deployed the locked model as an interactive, web-based risk-assessment tool to support early DRN screening and longitudinal monitoring. Cross-ethnic external validation in UK Biobank ($n = 502$; recruited 2006–2010) reproduced core protein signals and consistent effect directions, confirming robustness across populations. Principal methodological limitation lies in single time point proteomic assessment.

Conclusion

In this multi-cohort study, we present a proteomics- and ML–based precision prediction system for DRN. Pro-DRN substantially enhanced early risk stratification beyond conventional clinical factors and may support targeted screening and timely neuroprotective interventions, advancing molecularly guided strategies for diabetic eye disease prevention.

Author summary

Why was this study done?

- Diabetic retinal neurodegeneration is an early form of diabetes-related damage to the light-sensing layer of the eye, and growing evidence suggests that it also mirrors wider nerve injury elsewhere in the body; despite this, no practical tool exists to identify which people with diabetes are at highest risk before irreversible damage has occurred.
- Current screening relies on standard clinical risk factors and retinal photographs, both of which tend to detect damage only after it has already developed, leaving a narrow window for prevention.
- High-throughput plasma proteomics, combined with explainable AI, may offer a scalable way to identify blood-based signals of DRN and translate them into clinically useful prediction tools.

access should be directed to the data manager via email at liyuting@gzzoc.com. Requests will be reviewed within 90 days according to Center policy, and, if approved, an inter-institutional data use agreement specifying use for non-commercial academic research will be required. UK Biobank data are available through application via the official platform (http://www.ukbiobank.ac.uk) under a material transfer agreement (application number: 105658). All software used in this study is publicly available. The code used in this study can be accessed at https://github.com/ZOC-skl/Pro-DRN.

Funding: This work was supported by the GBRCE for Major Blinding Eye Diseases Prevention and Treatment, the Hainan Province Clinical Medical Center, the Science and Technology Projects in Guangzhou (2025A04J7150, W.W.), the National Natural Science Foundation of China (82371086 [W.W.], 82301253 [W.C.], 82571271 [S.C.]), the Natural Science Foundation of Guangdong Province (2026A1515010675, S.C.), the Guangdong Basic and Applied Basic Research Foundation (2022A151511, W.C.), the Projects of Research Center for Sharp Vision at The Hong Kong Polytechnic University (P0057931, S.T.), the Health and Medical Research Fund-Research Fellowship Scheme, Health Bureau, Hong Kong (07210207, S.T.), and the Lumitin Vision to Brightness Research Funding for the Young and middle-aged Ophthalmologists (BCF-KH-YK-20230803-03, S.C.). The funders had no role in study design, data collection and analysis, decision to publish, or preparation of the manuscript.

Competing interests: The authors have declared that no competing interests exist.

Abbreviations: BCVA, best corrected visual acuity; BRB, blood–retina barrier; COIP, Chinese Ocular Imaging Project; DCA, decision curve analysis; DR, diabetic retinopathy; DRD, diabetic retinal disease; DRN, diabetic retinal neurodegeneration; GDES, Guangzhou Diabetic Eye Study; GO, Gene Ontology; IOP, intraocular pressure; KEGG, Kyoto Encyclopedia of Genes and Genomes; KNN, k-nearest neighbors; mfERG, multifocal electroretinography; ML, machine learning; MNSI, Michigan Neuropathy Screening Instrument; NPX, Normalized Protein eXpression; OCT, optical coherence tomography; PEA, proximity extension assay; Pro-DRN, proteomics-based DRN model; RNFL, retinal nerve fiber layer; SHAP, SHapley

additive explanations; SVM, support vector machine; TABS, Topcon Advanced Boundary Segmentation; UKB, UK Biobank.

What did the researchers do and find?

- We analyzed two large prospective cohorts containing both high-resolution retinal imaging and measurements of thousands of blood proteins, using the Guangzhou Diabetic Eye Study for discovery ($n = 1{,}492$) and model development, and UK Biobank ($n = 502$) as an external validation cohort.
- We identified a group of blood proteins linked to DRN and developed a prediction model (Pro-DRN) based on these protein signals.
- To support clinical translation, we implemented the final model as an interactive online tool for real-time DRN risk assessment.

What do these findings mean?

- These findings suggest that a simple blood test analyzed with artificial intelligence may help identify people with diabetes who are at highest risk of early retinal nerve damage, well before visible damage appears on the retina.
- The results also reinforce the emerging view that retinal neurodegeneration reflects broader nerve and blood vessel injury in diabetes, so that a single eye-based test may carry information about the wider health of the nervous system.
- Important limitations should be kept in mind: the protein signals were measured in blood rather than directly in the retina, samples were obtained at a single time point, and the findings describe statistical associations rather than proven causes.

Introduction

Diabetes now affects more than half a billion adults worldwide, and the burden of visual impairment and blindness attributable to diabetic eye disease has remained stubbornly high across three decades of clinical progress [1–3]. Traditionally viewed as a microvascular disorder, diabetic retinal disease (DRD) is now recognized as a highly tissue-specific neurovascular complication [3–5]. Diabetic retinal neurodegeneration (DRN) often precedes microvascular pathology, independently driving visual dysfunction and disease progression [3,6,7]. Furthermore, DRN is regarded as a “window” into central nervous system complications of diabetes, such as cognitive impairment, dementia, and peripheral neuropathy [8–12].

Current screening for DRD relies on demographics, metabolic markers, and fundus photography, none of which can reliably flag individuals at highest risk of early neurodegeneration [3]. Advances in retinal imaging, including optical coherence tomography (OCT) and multifocal electroretinography (mfERG), enable in vivo assessment of neurodegeneration, yet they predominantly capture late structural or functional changes and fail to reflect underlying molecular processes [8–13]. By the time these structural or functional deficits become measurable, neurodegeneration is

often irreversible, creating an urgent need for accessible, interpretable, blood-based biomarkers that provide early warning while neural function remains plastic and amenable to intervention.

Circulating plasma proteins, as dynamic executors of organ crosstalk, offer a unique opportunity to bridge this diagnostic gap [14–16]. Their stability and measurability confer high translational potential. Previous proteomic studies of DRD have focused overwhelmingly on vascular leakage and intraocular fluids, leaving neurodegeneration-relevant signatures in the systemic circulation largely unexplored [17]. Advances in high-throughput plasma proteomics, such as Olink proximity extension assay, now enable precise quantification of thousands of proteins, providing the technical foundation for large-scale biomarker discovery. Integrating proteomics with longitudinal retinal imaging offers a path to systematically identify molecular drivers of DRN, detect high-risk individuals early, and reveal intervention targets [18,19].

The high dimensionality, nonlinearity, and complex interaction structure of modern proteomic data exceed the capacity of conventional statistical approaches. Multi-algorithm machine learning (ML), combined with explainable artificial intelligence, now enables robust signal extraction alongside interpretable biological insight [14]. Explainable AI methods, such as SHapley additive explanations (SHAP), map protein contributions, linking predictive performance to clinical and mechanistic insight. Integrated ML strategies combining proteomics, imaging, and clinical variables have shown remarkable promise for early diagnosis and risk stratification across diabetes, dementia, cardiovascular disease, and mortality [20–28]. To date, DRN biomarker research faces critical gaps [17,29–31]: (1) most studies are cross-sectional, limiting causal inference and predictive validation; (2) previous studies focuses on late structural or functional changes, with little longitudinal data capturing early neurodegeneration; (3) high-throughput proteomics is rarely integrated with imaging and clinical data in a unified framework; (4) biomarkers often lack cross-cohort validation and generalizability; and (5) interpretable, blood-based panels suitable for clinical workflows are largely absent, constraining early risk identification and targeted intervention.

To address these challenges, we leveraged two large prospective cohorts, the Guangzhou Diabetic Eye Study (GDES) and the UK Biobank (UKB), to test the hypothesis that circulating plasma proteins carry early, quantifiable signatures of DRN that can be translated into a clinically usable risk prediction tool. We quantified DRN using longitudinal OCT, integrated high-throughput plasma proteomics with multi-algorithm ML frameworks, and pursued four prespecified objectives: (i) to identify plasma protein signatures associated with baseline and longitudinal retinal nerve fiber layer (RNFL) thinning; (ii) to replicate core findings across ethnicities in UKB; (iii) to elucidate biological pathways linking these proteins to neurovascular instability; and (iv) to construct, interpret, and externally evaluate a proteomics-based DRN prediction system suitable for early risk stratification and targeted prevention.

Methods

Study design and participants

This international multi-cohort prospective observational study was designed to identify plasma protein biomarkers of DRN and to develop, interpret, and externally validate a machine learning based prediction model suitable for clinical translation. Two large-scale prospective cohorts were used: the Guangzhou Diabetic Eye Study (GDES) as the discovery and training cohort, and the UKB as the external validation cohort [32–35]. GDES is a large prospective cohort of adults with type 2 diabetes integrating plasma proteomics and multimodal retinal imaging. Between 2017 and 2019, over 3,000 individuals with type 2 diabetes aged 35–85 years were recruited in Guangzhou, China. Its proteomics sub-cohort (GDES-PPP) served as the development dataset. UKB is a population-based multicenter cohort that enrolled over 500,000 participants aged 40–69 years across the United Kingdom between 2006 and 2010. Its proteomics subproject (UKB-PPP) was used for external validation. Both studies adhered to the Declaration of Helsinki. Ethical approvals were obtained from the Zhongshan Ophthalmic Center Ethics Committee (2017KYPJ094) and the North West Multi-Centre Research Ethics Committee (11/NW/0382). All participants provided written informed consent. The study was reported following STROBE guidelines (S1 Checklist) and TRIPOD statement (S2 Checklist).

Participants from GDES were included in two steps. First, Population 1 comprised 1,492 participants with type 2 diabetes but no diabetic retinopathy (DR) at baseline, all with baseline proteomics and qualified OCT data, and was used for cross-sectional analysis of plasma protein associations with retinal nerve fiber layer thickness. Second, to focus on neurodegeneration independent of vascular or exudative lesions, we identified within Population 1 a subgroup of 1,218 participants who remained free of DR during 6 years of follow-up (Population 2). All individuals in Population 2 had baseline proteomic data and OCT scans, with OCT repeated at years 2, 4, and 6 after enrollment. This population was used for longitudinal RNFL thinning analyses, pathway enrichment, and ML model development. For external validation, Population 3 comprised 502 UKB participants with type 2 diabetes but no baseline DR (S1 Table), all with available proteomics and a single time point OCT measure obtained at the UKB baseline ocular imaging assessment (2009–2010). Across both cohorts, we excluded participants lacking plasma proteomics or analyzable OCT-derived RNFL measures, applied prespecified OCT quality control, and excluded glaucoma, dementia, and other ocular conditions that could confound RNFL measurements (S1 Text).This cohort was used for cross-sectional analyses of associations between baseline protein levels and RNFL thickness to evaluate the robustness and generalizability of protein associations across diverse ethnicities and healthcare systems. The prespecified Phase I–IV objectives are summarized in the Results.

Retinal OCT imaging protocols

High-resolution OCT was used to quantify RNFL thickness, a key biomarker of DRN [36–39]. Standardized acquisition and analysis procedures were applied in both cohorts: (1) GDES cohort: Disc-centered three-dimensional OCT scans were acquired under dark-room conditions using the Topcon DRI OCT Triton swept-source system (Topcon Corporation, Japan), featuring a 1,050 nm light source, 100,000 A-scans/s, and 8 μm axial resolution. Peripapillary RNFL thickness was measured along a 360° circular path, 3.4 mm in diameter centered on the optic disc. (2) UKB cohort: Three-dimensional volume scans were obtained using the Topcon 3D OCT-1000 Mk II (Topcon Corporation, Japan) under similar dark-room conditions, with 18,000 A-scans/s and 6 μm axial resolution.

In GDES cohorts, scans were performed after adequate mydriasis by trained technicians, with internal eye-tracking to ensure accuracy. In UK Biobank, OCT imaging followed the standard UKB protocol and was performed without pharmacologic mydriasis. RNFL segmentation was performed automatically using the Topcon Advanced Boundary Segmentation (TABS) algorithm, defining RNFL thickness as the perpendicular distance from the internal limiting membrane to the inner border of the ganglion cell layer. The peripapillary region was divided into four quadrants (superior, inferior, temporal, and nasal), and both average and quadrant-specific RNFL thicknesses were calculated. All images were visually inspected by two experienced OCT technicians, with manual correction applied as needed. Only high-quality scans were included; images with poor quality, motion artifacts, or signal strength index <60 were excluded. For participants with valid data in both eyes, right-eye measurements were preferentially used (S1 Text).

DRN definition and phenotype quantification

DRN was defined as a pathological state marked by accelerated RNFL loss. Using repeated OCT measurements at baseline, year 2, year 4, and year 6, the annualized RNFL thinning rate was estimated using a linear mixed-effects model (median follow-up, 5.49 years). In the GDES cohort, participants were ranked by this rate: those in the fastest quartile (Q1) were classified as DRN, while the remaining participants (Q2–Q4) served as non-DRN controls. This objective, biologically grounded definition identifies individuals with pronounced neurodegenerative phenotypes, providing a precise foundation for biomarker discovery and predictive modeling. In sensitivity analyses, we additionally defined DRN as (i) the fastest 10% of RNFL thinners (DRN-Top10Slope), (ii) thin/abnormal pRNFL on the OCT report at the final available visit (DRN-ThinLast), and (iii) an annualized RNFL thinning rate below the lower normative reference limit derived from the 6-year longitudinal OCT data of healthy participants in the Chinese Ocular Imaging Project (COIP) cohort (DRN-ExcessLoss; S1 Text).

Plasma proteomics protocols

High-throughput plasma proteomics was performed using Olink Proximity Extension Assay (PEA) technology in both GDES and UKB cohorts, following standardized protocols for sample collection, preprocessing, measurement, and quality control. Laboratory personnel were blinded to clinical data. In GDES, a targeted approach employed the Olink Cardiometabolic Panel, measuring 361 proteins relevant to metabolic regulation, cardiovascular function, and inflammatory pathways, all mechanistically plausible for diabetes and its neuro-complications. This focused panel enabled deep, pathway-specific interrogation of DRN biology. The UKB cohort applied a broader proteomic panel, providing an independent platform for validation and assessment of biomarker robustness and generalizability. All proteins assayed in GDES were included in the UKB-PPP protein set; therefore, all cross-cohort replication and validation were conducted using this shared set, which comprised the full GDES panel. Rigorous quality control was implemented in both cohorts: within-plate CV < 10% and between-plate CV < 20%, with inter-plate normalization using control samples. Raw counts were normalized by extension controls and $\log_2$-transformed to generate Normalized Protein eXpression (NPX) values, minimizing technical variation [26,27]. All NPX data were standardized and batch-corrected for downstream statistical analyses (S1 Text).

Covariate measurements

Covariates were collected using rigorous, standardized procedures within the prospective GDES cohort. Data encompassed demographics, lifestyle factors, clinical indices, and ophthalmic parameters, ensuring accuracy and comparability. Demographic (age, sex), socioeconomic (education, household income), and lifestyle data (smoking, alcohol, physical activity) were obtained through validated electronic questionnaires combined with face-to-face interviews. Diabetes-related information, including duration, medication use, and complications, was ascertained via medical record review and patient interview, with diagnoses based on international criteria and independently confirmed by two internists.

Anthropometric and biochemical measurements were performed by trained staff using standardized protocols. Height and weight were measured with calibrated instruments; blood pressure was recorded on the non-dominant arm after 5 min of rest, with three consecutive measures averaged. Fasting venous blood was collected for HbA1c (HPLC), lipid panel (total cholesterol, triglycerides, high-density lipoprotein cholesterol, low-density lipoprotein cholesterol via enzymatic colorimetry), renal function markers (serum creatinine, estimated glomerular filtration rate), serum urate, and urinary albumin. All assays incorporated internal quality-control samples and participated in external quality assessment programs.

Ophthalmic evaluations followed standardized protocols. Slit-lamp biomicroscopy (Haag-Streit BQ-900, Switzerland) assessed the anterior segment. Best corrected visual acuity (BCVA) was measured using Early Treatment Diabetic Retinopathy Study (ETDRS) LogMAR charts (Precision Vision, USA) at 4 m. Refractive status was measured with an autorefractor (Topcon KR-8800, Japan); intraocular pressure (IOP) was measured with a non-contact tonometer (Topcon CT-1, Japan); axial length was measured by optical biometry (Lenstar LS900, Haag-Streit, Switzerland). All assessments were conducted by trained personnel following uniform procedures. Baseline covariate protocols for the UKB cohort are detailed in S1 Text.

Functional enrichment analysis

To elucidate the biological mechanisms underlying plasma proteins associated with DRN, we conducted comprehensive functional enrichment analyses. Using the set of plasma protein markers significantly linked to DRN, we applied the clusterProfiler R package (v4.0) to perform enrichment against Gene Ontology (GO), Kyoto Encyclopedia of Genes and Genomes (KEGG), and Reactome databases. Analyses encompassed biological process (BP), cellular component (CC), and molecular function (MF) terms, KEGG pathways, and Reactome reaction networks, systematically profiling the biological activities, subcellular localization, molecular functions, and pathway interactions of the target proteins. Enrichment

significance was assessed using the hypergeometric (Fisher's) test, with *P* values adjusted for multiple comparisons by the Benjamini–Hochberg method (FDR) < 0.05 was considered statistically significant.

Pro-DRN model construction

To develop a proteomics-driven risk prediction model, we selected plasma proteins significantly associated with longitudinal RNFL thinning in the discovery stage as features. The GDES cohort was randomly split into training and test sets at an 8:2 ratio, stratified by DRN status to preserve the proportion of positive cases. All data preprocessing, feature selection, and hyperparameter tuning were confined to the training set; the test set was used solely for final evaluation to prevent information leakage.

To ensure robustness and cross-validate the proteomic signal, we trained eight supervised learning algorithms in parallel: XGBoost, LightGBM, Random Forest, support vector machine (SVM), Neural Network, Logistic Regression, k-nearest neighbors (KNN), and Decision Tree. Stratified 10-fold cross-validation was applied within the training set, using fold-averaged C-index as the primary metric. Simpler models were favored when discrimination was comparable, and in-fold calibration was evaluated. Gradient-boosting models incorporated early stopping to mitigate overfitting, and hyperparameters were optimized via grid search or Bayesian methods based on cross-validation performance (S1 Text).

The final model was chosen based on consistent superiority across cross-validation and the independent test set. Model interpretability was assessed using Shapley values, quantifying each protein's contribution to predictions. Two metrics were computed: (1) global feature importance based on mean absolute SHAP values, reflecting overall contribution, and (2) individual-level feature attributions, indicating the direction and magnitude of each protein's effect on specific predictions.

Clinically meaningful outcomes for external validity

To assess clinical relevance beyond the OCT-based DRN phenotype, we examined associations between the baseline proteomic risk score and subsequent vision impairment and diabetic peripheral neuropathy. Vision impairment was defined as a loss of ≥15 ETDRS letters in best-corrected visual acuity during follow-up. Diabetic peripheral neuropathy was defined according to a previously published approach as a dichotomized Michigan Neuropathy Screening Instrument (MNSI) patient questionnaire score ≥4.

Statistical analysis

Statistical analysis protocol is provided in the Supplementary Materials. Analyses were conducted using R (v4.5.1) and Stata/MP (v18.0). Continuous variables are presented as mean ± SD or median (IQR), and categorical variables as counts (percentages). Between-group comparisons used *t*-tests or χ^2 tests as appropriate. All tests were two-sided, with $P < 0.05$ considered statistically significant. Multiple comparisons were controlled using the Benjamini–Hochberg method, with FDR < 0.05 considered significant.

In Population 1, cross-sectional associations between plasma proteins and RNFL thickness were assessed using multivariable linear regression, adjusting for age, sex, smoking, systolic blood pressure, HbA1c, and diabetes duration. Proteins with FDR < 0.05 were deemed significantly associated. In Population 2, the same covariates were used to test longitudinal associations with RNFL thinning rate.

For DRN prediction, participants in the top 25% of RNFL decline were classified as DRN cases, with the remainder as controls. Samples were randomly split 8:2 into training and testing sets. Using selected protein features, Pro-DRN were built with eight ML algorithms. The optimal model was selected based on C-index and generalization performance. Model evaluation included comparisons with nine individual traditional predictors and a baseline age–sex model using C-index and ΔC as discrimination metrics. Pro-DRN was further integrated into multiple existing clinical prediction models (Aspelund, Hippisley, Dagliati, ISDR, JDC, Tarasewicz, and a combined "All" model; variable composition was summarized in

S13 Table) to assess incremental predictive value [40–48]. Risk reclassification was quantified using NRI and IDI, calibration assessed with calibration plots. Decision curve analysis (DCA) was used to evaluate clinical utility, and the net-benefit definition and underlying assumptions are described in S1 Text. The final model was implemented as an interactive online tool using the R Shiny [49].

For external validation, UKB-PPP participants with diabetes and available proteomics and OCT data were analyzed as an independent cohort. Cross-sectional analyses were fully replicated using the same covariates and modeling strategy to assess consistency in effect direction and significance of key protein associations. We additionally validated the locked Pro-DRN model using a cross-sectional thin-RNFL outcome, reporting discrimination (C-index) and calibration (Brier score and Hosmer–Lemeshow test).

Results

Study design and framework

This study leveraged two large-scale prospective cohorts: the Guangzhou Diabetic Eye Study (GDES) and the UK Biobank (UKB) [32–35]. The GDES Proteomic Program (GDES-PPP) served as the discovery and model development dataset, and the UKB Proteomic Program (UKB-PPP) provided external validation. Participants with both high-quality Olink proteomic data and RNFL measurements by OCT were included.

The analytical framework comprised four modules (Fig 1). Module I involved cross-sectional protein screening using GDES-PPP baseline data to identify plasma proteins associated with DRN. Module II performed longitudinal analyses among participants who remained DRD-free over six years, evaluating associations between candidate proteins and RNFL thinning rates, and revealing underlying biological pathways via pathways enrichment analyses. Module III built and evaluated ML–based DRN prediction models, assessing performance through interpretability metrics, predictive accuracy, and comparison with conventional models [49]. Module IV validated DRN-associated protein signatures in UKB participants with type 2 diabetes to confirm generalizability across populations.

Baseline characteristics

The baseline characteristics of the three analytical populations are summarized in Tables 1 (Population 2) and S1 (Populations 1 and 3). In GDES-PPP, 1,492 participants with type 2 diabetes, with baseline plasma proteomics and qualified macular OCT scans (Population 1) were included in cross-sectional analyses (mean age 64.4 ± 7.5 years; 57.3% women). A total of 1,218 DRD-free over six years participants were included in longitudinal analyses (Population 2). Based on quartiles of RNFL thinning rate, the fastest-declining quartile (Q1, $n = 305$, 25.0%) defined the DRN group, and Q2–Q4 ($n = 913$) the non-DRN group. Participants in the DRN group were older, had a longer duration of diabetes, and higher systolic blood pressure at baseline (Table 1). Baseline pRNFL thickness was comparable between DRN progressors and non-DRN participants (110.07 ± 13.67 μm versus 109.81 ± 12.29 μm; $P = 0.756$). For external validation, 502 UKB-PPP participants with type 2 diabetes, no DR or other retinal disease at the time of OCT imaging, and available proteomic and OCT data (mean age 58.3 ± 7.6 years; 50.8% women) provided an independent cross-ethnic dataset (S1 Table).

Plasma proteins associated with DRN

In the GDES-PPP discovery cohort, cross-sectional analyses identified 72 plasma proteins significantly associated with average RNFL thickness (FDR < 0.05; Fig 2A and 2B). Among these, the 10 proteins most strongly negatively correlated with RNFL thickness were TFF3, MCFD2, CST3, FAM3C, CGREF1, NECTIN2, NPDC1, CLC, HSPG2, and DEFA1, with higher protein levels associated with thinner baseline RNFL (S2 Table). The adjusted effect sizes (β) ranging from −2.014 μm (95% CI [−2.800, −1.229]) to −0.983 μm (95% CI [−1.702, −0.265]) per 1–SD increase. In longitudinal analyses, 71 of these proteins were confirmed being significantly associated with RNFL thinning rate (FDR < 0.05; S3 Table). The top 10

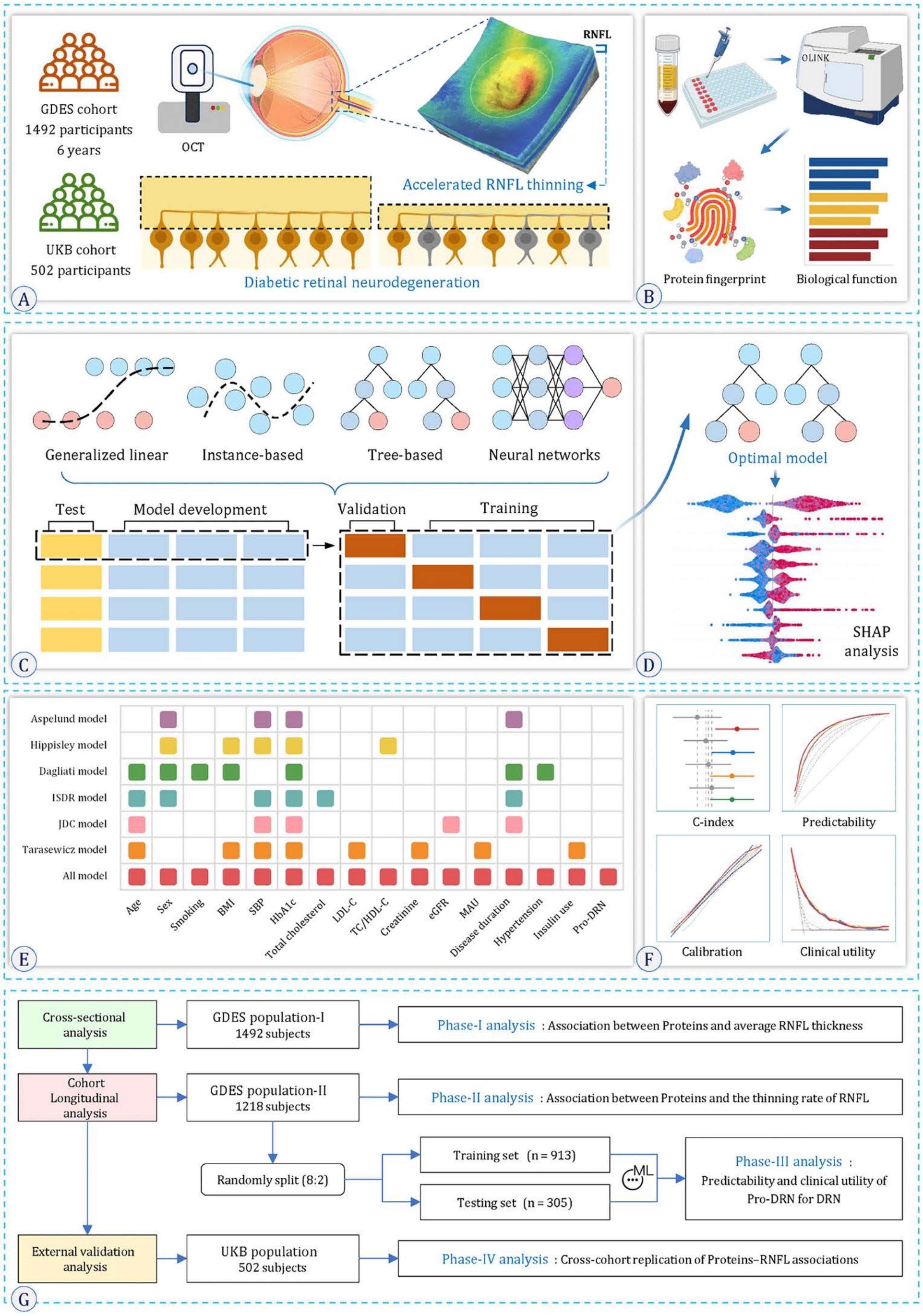

Fig 1. Study design and workflow. (A) Cohorts and phenotype. Participants from the Guangzhou Diabetic Eye Study (GDES; discovery cohort with 6-year follow-up; $n = 1{,}492$ baseline) and the UK Biobank (UKB; external replication; $n = 502$) underwent optical coherence tomography (OCT) to quantify retinal nerve fiber layer (RNFL) thickness; accelerated RNFL thinning operationalizes DRN. **(B)** Plasma proteomics and functional annotation. EDTA plasma was profiled using a proximity-extension assay (Olink) to derive protein fingerprints and functional enrichment. **(C)** Model development. GDES was randomly split (8:2) into training and an untouched test set. Four families of learners were trained (generalized-linear, instance-based, tree-based, and neural-network models), with internal validation for hyperparameter tuning. **(D)** Model selection and interpretability. The optimal model was chosen based on validation performance and interpreted with SHAP (Shapley additive explanations) to quantify feature contributions. **(E)** Clinical frameworks. Candidate clinical variables were organized according to established risk models (Aspelund, Hippisley, Dagliati, ISDR, JDC, Tarasewicz) and a comprehensive "All model." **(F)** Performance assessment. Discrimination (C-index/ROC), calibration, and clinical utility (decision-curve analysis) were evaluated

in the hold-out test set. **(G)** Analysis phases. Phase I: cross-sectional protein–RNFL thickness associations; Phase II: longitudinal associations with RNFL thinning rate; Phase III: development and clinical evaluation of Pro-DRN in GDES; Phase IV: cross-cohort replication in UKB. **Abbreviations**: RNFL, retinal nerve fiber layer; OCT, optical coherence tomography; SHAP, Shapley additive explanations.

https://doi.org/10.1371/journal.pmed.1004868.g001

Table 1. Baseline characteristics of Guangzhou Diabetic Eye Study (GDES) participants with Non-DR during follow-up.

Characteristics	Overall	Non-DRN	DRN	*P* value*
No. of subjects	1,218	913	305	
Age, year	64.06 ± 7.34	63.36 ± 7.43	66.17 ± 6.65	**<0.001**
Sex				0.184
Female	700 (57.5%)	535 (58.6%)	165 (54.1%)	
Male	518 (42.5%)	378 (41.4%)	140 (45.9%)	
Educational attainment				0.749
<High School	396 (32.5%)	295 (32.3%)	101 (33.1%)	
High School	467 (38.3%)	356 (39.0%)	111 (36.4%)	
University and above	350 (28.7%)	259 (28.4%)	91 (29.8%)	
Missing	5 (0.4%)	3 (0.3%)	2 (0.7%)	
Income, CNY				0.519
≤24k	51 (4.2%)	39 (4.3%)	12 (3.9%)	
24k–60k	823 (67.6%)	621 (68.0%)	202 (66.2%)	
60k–120k	169 (13.9%)	129 (14.1%)	40 (13.1%)	
≥120k	17 (1.4%)	14 (1.5%)	3 (1.0%)	
Missing	158 (13.0%)	110 (12.0%)	48 (15.7%)	
Smoking status				0.481
Ever/Current	1,074 (88.2%)	809 (88.6%)	265 (86.9%)	
Never	144 (11.8%)	104 (11.4%)	40 (13.1%)	
Alcohol				0.534
Ever/Current	1,107 (90.9%)	833 (91.2%)	274 (89.8%)	
Never	111 (9.1%)	80 (8.8%)	31 (10.2%)	
Body mass index, kg/m^2	24.53 ± 3.16	24.45 ± 3.14	24.76 ± 3.19	0.133
SBP, mmHg	133.44 ± 17.88	132.59 ± 17.47	135.97 ± 18.86	**0.004**
DBP, mmHg	70.77 ± 9.98	70.73 ± 9.92	70.88 ± 10.18	0.821
Duration of diabetes, year	8.39 ± 6.68	8.15 ± 6.56	9.12 ± 6.99	**0.027**
HbA1c, %	6.92 ± 1.29	6.94 ± 1.28	6.86 ± 1.29	0.324
Use of insulin	235 (19.3%)	177 (19.4%)	58 (19.0%)	0.950
Total cholesterol, mmol/L	4.80 ± 1.05	4.81 ± 1.06	4.78 ± 1.02	0.671
LDL-C, mmol/L	3.02 ± 0.93	3.02 ± 0.95	3.02 ± 0.89	0.982
HDL-C, mmol/L	1.30 ± 0.40	1.31 ± 0.41	1.28 ± 0.40	0.336
Serum creatine, mmol/L	70.39 ± 19.18	68.70 ± 17.61	75.45 ± 22.55	**<0.001**
Microalbuminuria, mg/mL	3.61 ± 10.73	2.68 ± 7.49	6.43 ± 16.85	**<0.001**
BCVA, LogMAR	0.18 ± 0.15	0.17 ± 0.14	0.23 ± 0.17	**<0.001**
Axial length, mm	23.52 ± 1.09	23.48 ± 1.07	23.64 ± 1.16	**0.024**
Baseline pRNFL thickness, μm	109.88 ± 12.65	109.81 ± 12.29	110.07 ± 13.67	0.756

**P* values were calculated using Student *t* test for continuous variables and the χ^2 test for categorical variables. Bold values indicate statistical significance.

DR, diabetic retinopathy; SBP, systolic blood pressure; DBP, diastolic blood pressure; HbA1c, glycosylated hemoglobin; LDL-C, low-density lipoprotein cholesterol; HDL-C, high-density lipoprotein cholesterol; BCVA, best-corrected visual acuity.

https://doi.org/10.1371/journal.pmed.1004868.t001

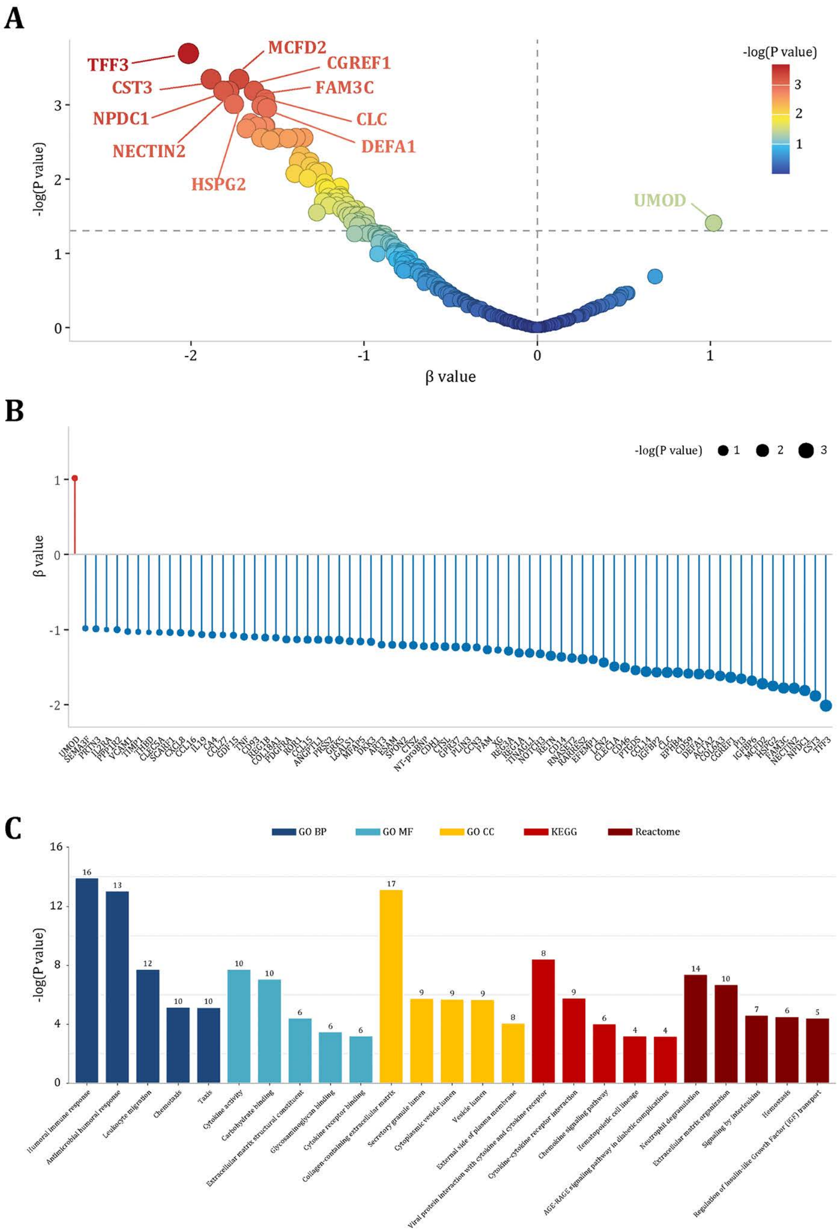

Fig 2. DRN-associated proteins and functional enrichment. (A) Volcano plot summarizing DRN-associated proteins. The x-axis represents standardized β coefficients; the y-axis shows statistical significance as $-\log_{10}(P)$ values. Highlighted proteins (e.g., TFF3, CST3, MCFD2, FAM3C, NPDC1, UMOD) exceed predefined thresholds. **(B)** Ranked effect sizes (lollipop plot) for all proteins; point size reflects $-\log_{10}(P)$. Most associations are negative (protective), with UMOD showing a positive effect. **(C)** Over-representation analysis of FDR-significant proteins across Gene Ontology (GO), Kyoto Encyclopedia of Genes and Genomes (KEGG), and Reactome databases. Numbers above bars denote the count of proteins contributing to each term. Enriched pathways including inflammatory response, extracellular matrix remodeling, and neurotrophic signaling.

https://doi.org/10.1371/journal.pmed.1004868.g002

proteins most strongly linked to accelerated thinning included CST3, HSPG2, NECTIN2, COL6A3, ACTA2, NPDC1, CD59, PTGDS, RNASET2, and FAM3C, with β values ranging from −0.445 μm/year (95% CI [−0.518, −0.372]) to −0.112 μm/year (95% CI [−0.173, −0.050]). Notably, UMOD consistently exhibited a protective effect, with higher levels associated with thicker baseline RNFL ($\beta = 1.019$ μm; 95% CI [0.273, 1.764]) and slower thinning over time ($\beta = 0.256$ μm/year; 95% CI

[0.192, 0.320]). These associations between the RNFL thinning rate and proteins remained robust in sensitivity analyses (S4 Table).

Functional annotation of DRN-associated proteins

Functional enrichment analyses using the GO, Kyoto Encyclopaedia of Genes and Genomes (KEGG), and Reactome revealed four interrelated biological processes (Fig 2C): (1) inflammation and immune recruitment (Cytokine–cytokine receptor interaction, Chemokine signaling, Leukocyte migration, Neutrophil degranulation); (2) basement membrane and ECM remodeling (Collagen-containing ECM, ECM organization, Glycosaminoglycan binding, Basement membrane); (3) membrane receptor function and blood–retina barrier integrity (Cell adhesion molecules, Cytokine receptor binding); and (4) microvascular homeostasis and metabolic stress (Complement and coagulation cascades, Hemostasis, AGE–RAGE signaling). These enrichment results implicate chronic inflammation, extracellular matrix dysregulation, receptor-mediated vascular dysfunction, and microvascular-metabolic stress as interlinked molecular correlates of early DRN.

Proteomics-based DRN (Pro-DRN) prediction model

We constructed Pro-DRN using plasma protein markers identified in GDES-PPP, employing eight widely used ML algorithms: XGBoost, LightGBM, Random Forest, Neural Network, Logistic Regression, K-Nearest Neighbors, Support Vector Machine (SVM), and Decision Tree. Models were trained using an 8:2 training/test split, with hyperparameters optimized via cross-validation, and performance evaluated in an independent test set (Fig 3; S5 Table).

In the training set, model C-index ranged from 0.760 to 0.984, with Random Forest 0.984 (95% CI [0.977, 0.990]), XGBoost 0.979 (95% CI [0.972, 0.986]), and SVM 0.947 (95% CI [0.933, 0.961]) performing best. In the test set, C-indices ranged from 0.736 to 0.860, with XGBoost 0.860 (95% CI [0.810, 0.911]), LightGBM 0.832 (95% CI [0.777–0.886]), and Random Forest 0.816 (95% CI [0.756–0.876]) leading. XGBoost was selected as the final model on the basis of its superior balance between discrimination, generalisability, and training stability, and was used for all subsequent Pro-DRN scoring, feature-importance interpretation, and clinical utility analyses.

Importance ranking of Pro-DRN plasma proteins

To interpret the model, we applied Shapley values, providing both global and individual-level insights while accounting for protein collinearity and complex interactions (Fig 4). Globally, the top 10 proteins contributing to DRN risk were ACTA2, COL6A3, CCL27, NT-proBNP, CD46, MCFD2, CTSZ, CCL15, RARRES2, and HSPG2 (Fig 4A). Beeswarm plots confirmed consistent effect directions across individual samples, with higher expression of most top proteins increasing predicted risk.

In high-risk individuals (predicted probability = 0.85), COL6A3, ACTA2, CCL16, REG3A, HSPG2, PAM, ART3, and TIMP1 jointly drove risk, demonstrating a multi-protein additive effect (Fig 4B). The concordance between global and individual explanations indicates that the discriminative power of Pro-DRN arises from the integrated contributions of multiple proteins rather than from any single dominant feature, a property that simultaneously enhances interpretability and predictive robustness.

Pro-DRN substantially improves DRN risk discrimination

We systematically evaluated the predictive performance of Pro-DRN in the test set. As a standalone predictor, Pro-DRN achieved excellent discrimination (C-index = 0.860; 95% CI [0.810, 0.911]), far exceeding any single conventional predictor (C-index: 0.500 to 0.688; S6 Table), highlighting the independent and superior value of plasma proteomic features. Integrating Pro-DRN with a baseline model of age and sex increased the C-index from 0.697 to 0.877 (95% CI [0.834, 0.921]), representing a 25.9% relative improvement (Table 2).

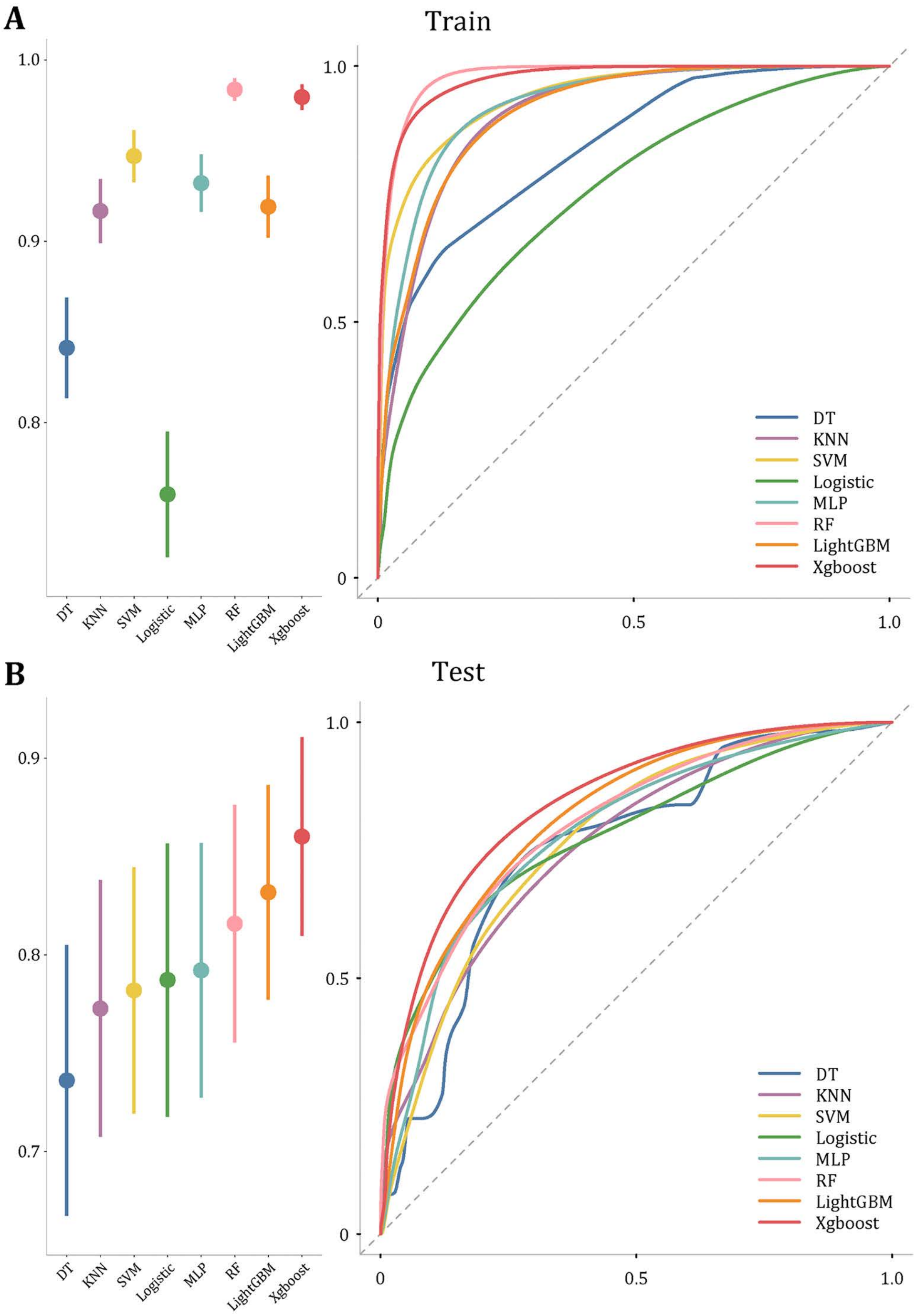

Fig 3. Performance of machine-learning models for DRN prediction. (A) Training set. Left, model discrimination summarized as AUC (C-index) with 95% confidence intervals; right, ROC curves for each model. **(B)** Held-out test set, displayed as in **(A)**. The dashed diagonal denotes chance performance (AUC = 0.5). Across both sets, gradient-boosting models (XGBoost and LightGBM) show the highest discrimination. **Abbreviations**: DT, decision tree; KNN, k-nearest neighbors; SVM, support vector machine; MLP, multilayer perceptron; RF, random forest.

https://doi.org/10.1371/journal.pmed.1004868.g003

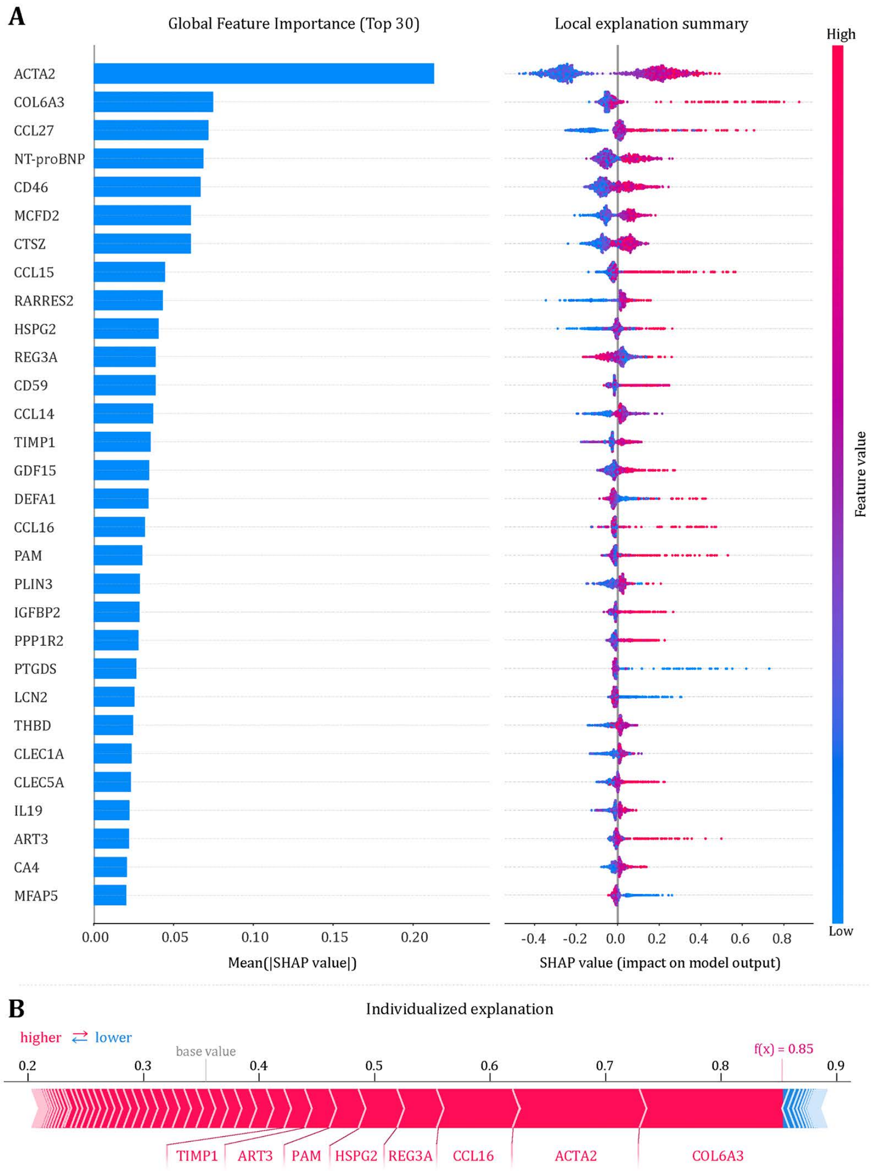

Fig 4. Global and individual explanations of the Pro-DRN model. (A) XGBoost interpretation. Left: global feature importance ranked by mean |SHAP| for the top 30 proteins (longer bars = greater overall contribution). Right: SHAP beeswarm plot showing the signed effect of each feature on the predicted probability of DRN (x-axis, SHAP value); red/blue indicate higher/lower feature values. High-impact proteins include ACTA2, COL6A3, CCL27, NT-proBNP, CD46, MCFD2, CTSZ, CCL15, RARRES2 and HSPG2. **(B)** Individual explanation (force plot) for a representative participant with predicted risk f(x)=0.85. Red features push the prediction above the model base value (increase risk), and blue features pull it down; arrow width reflects absolute SHAP contribution.

https://doi.org/10.1371/journal.pmed.1004868.g004

To assess incremental values, Pro-DRN was added to six established clinical models (Fig 5A and 5B). All models showed consistent gains in discrimination, with average ΔC-index ranging from 0.137 to 0.159, and the fully adjusted model (all clinical variables plus Pro-DRN) achieved a C-index of 0.908 (95% CI [0.869, 0.948]; Table 2). Importantly, the Pro-DRN model achieved discrimination that was comparable to, and modestly higher than, the model built using all measured proteins (S7 Table). Risk reclassification analyses further confirmed its value: average IDI increased by 23.3%

Table 2. Incremental predictive value of the Pro-DRN across established models.

	C-index (95% CI)		Improvement	*P* value*
	Benchmark			Incorporating Pro-DRN
**XGboost model**				
Age&Sex	0.697 (0.626, 0.768)	0.877 (0.834, 0.921)	25.87%	**4.20×10^{-07}**
Aspelund model	0.733 (0.665, 0.800)	0.888 (0.847, 0.930)	21.28%	**3.07×10^{-06}**
Hippisley model	0.739 (0.670, 0.808)	0.898 (0.858, 0.937)	21.52%	**2.31×10^{-06}**
Dagliati model	0.743 (0.676, 0.809)	0.898 (0.859, 0.938)	20.96%	**1.12×10^{-06}**
ISDR model	0.733 (0.665, 0.801)	0.889 (0.847, 0.930)	21.20%	**3.80×10^{-06}**
JDC model	0.748 (0.681, 0.815)	0.889 (0.848, 0.930)	18.85%	**8.91×10^{-06}**
Tarasewicz model	0.760 (0.694, 0.827)	0.897 (0.857, 0.937)	18.01%	**2.16×10^{-05}**
All model	0.812 (0.750, 0.874)	0.908 (0.869, 0.948)	11.89%	**5.19×10^{-04}**
**LightGBM model**				
Age&Sex	0.697 (0.626, 0.768)	0.848 (0.799, 0.897)	21.67%	**1.32×10^{-05}**
Aspelund model	0.733 (0.665, 0.800)	0.860 (0.814, 0.906)	17.38%	**1.03×10^{-04}**
Hippisley model	0.739 (0.670, 0.808)	0.875 (0.830, 0.919)	18.36%	**4.73×10^{-05}**
Dagliati model	0.743 (0.676, 0.809)	0.873 (0.829, 0.917)	17.57%	**2.85×10^{-05}**
ISDR model	0.733 (0.665, 0.801)	0.861 (0.815, 0.907)	17.49%	**1.05×10^{-04}**
JDC model	0.748 (0.681, 0.815)	0.862 (0.816, 0.908)	15.24%	**2.25×10^{-04}**
Tarasewicz model	0.760 (0.694, 0.827)	0.873 (0.828, 0.917)	14.79%	**2.61×10^{-04}**
All model	0.812 (0.750, 0.874)	0.891 (0.850, 0.932)	9.74%	**0.002**
**RF model**				
Age&Sex	0.697 (0.626, 0.768)	0.844 (0.791, 0.897)	21.06%	**4.06×10^{-05}**
Aspelund model	0.733 (0.665, 0.800)	0.868 (0.822, 0.915)	18.51%	**8.37×10^{-05}**
Hippisley model	0.739 (0.670, 0.808)	0.872 (0.824, 0.919)	18.02%	**8.21×10^{-05}**
Dagliati model	0.743 (0.676, 0.809)	0.880 (0.837, 0.924)	18.55%	**2.10×10^{-05}**
ISDR model	0.733 (0.665, 0.801)	0.868 (0.822, 0.915)	18.45%	**1.01×10^{-04}**
JDC model	0.748 (0.681, 0.815)	0.868 (0.822, 0.915)	16.08%	**2.40×10^{-04}**
Tarasewicz model	0.760 (0.694, 0.827)	0.875 (0.828, 0.921)	15.03%	**4.09×10^{-04}**
All model	0.812 (0.750, 0.874)	0.897 (0.856, 0.937)	10.47%	**0.002**
**NN model**				
Age&Sex	0.697 (0.626, 0.768)	0.814 (0.757, 0.871)	16.75%	**6.99×10^{-04}**
Aspelund model	0.733 (0.665, 0.800)	0.835 (0.784, 0.886)	14.00%	**0.001**
Hippisley model	0.739 (0.670, 0.808)	0.842 (0.790, 0.895)	14.00%	**6.52×10^{-04}**
Dagliati model	0.743 (0.676, 0.809)	0.848 (0.799, 0.898)	14.26%	**5.29×10^{-04}**
ISDR model	0.733 (0.665, 0.801)	0.839 (0.788, 0.889)	14.41%	**0.001**
JDC model	0.748 (0.681, 0.815)	0.842 (0.791, 0.893)	12.58%	**0.001**
Tarasewicz model	0.760 (0.694, 0.827)	0.854 (0.804, 0.903)	12.28%	**0.001**
All model	0.812 (0.750, 0.874)	0.871 (0.824, 0.917)	7.23%	**0.010**
**Logistic model**				
Age&Sex	0.697 (0.626, 0.768)	0.824 (0.767, 0.881)	18.21%	**3.51×10^{-04}**
Aspelund model	0.733 (0.665, 0.800)	0.841 (0.788, 0.894)	14.82%	**8.63×10^{-04}**
Hippisley model	0.739 (0.670, 0.808)	0.849 (0.795, 0.902)	14.86%	**2.74×10^{-04}**
Dagliati model	0.743 (0.676, 0.809)	0.854 (0.803, 0.905)	15.00%	**2.16×10^{-04}**
ISDR model	0.733 (0.665, 0.801)	0.844 (0.792, 0.897)	15.17%	**8.07×10^{-04}**
JDC model	0.748 (0.681, 0.815)	0.846 (0.793, 0.899)	13.12%	**0.001**
Tarasewicz model	0.760 (0.694, 0.827)	0.860 (0.810, 0.909)	13.05%	**7.99×10^{-04}**

(Continued)

Table 2. (Continued)

	C-index (95% CI)		Improvement	*P* value*
	Benchmark			Incorporating Pro-DRN
All model	0.812 (0.750, 0.874)	0.875 (0.825, 0.924)	7.76%	**0.007**
**KNN model**				
Age&Sex	0.697 (0.626, 0.768)	0.816 (0.760, 0.873)	17.12%	**3.74×10^{-04}**
Aspelund model	0.733 (0.665, 0.800)	0.841 (0.790, 0.891)	14.75%	**6.34×10^{-04}**
Hippisley model	0.739 (0.670, 0.808)	0.852 (0.802, 0.901)	15.27%	**2.53×10^{-04}**
Dagliati model	0.743 (0.676, 0.809)	0.851 (0.803, 0.899)	14.57%	**3.31×10^{-04}**
ISDR model	0.733 (0.665, 0.801)	0.839 (0.789, 0.890)	14.49%	**8.01×10^{-04}**
JDC model	0.748 (0.681, 0.815)	0.842 (0.792, 0.892)	12.60%	**0.002**
Tarasewicz model	0.760 (0.694, 0.827)	0.852 (0.804, 0.901)	12.10%	**0.001**
All model	0.812 (0.750, 0.874)	0.875 (0.830, 0.919)	7.74%	**0.008**
**SVM model**				
Age&Sex	0.697 (0.626, 0.768)	0.810 (0.755, 0.866)	16.26%	**6.73×10^{-04}**
Aspelund model	0.733 (0.665, 0.800)	0.826 (0.774, 0.878)	12.73%	**0.002**
Hippisley model	0.739 (0.670, 0.808)	0.838 (0.786, 0.890)	13.43%	**7.55×10^{-04}**
Dagliati model	0.743 (0.676, 0.809)	0.834 (0.783, 0.886)	12.36%	**0.001**
ISDR model	0.733 (0.665, 0.801)	0.828 (0.776, 0.879)	12.92%	**0.002**
JDC model	0.748 (0.681, 0.815)	0.826 (0.773, 0.879)	10.49%	**0.003**
Tarasewicz model	0.760 (0.694, 0.827)	0.841 (0.791, 0.892)	10.65%	**0.003**
All model	0.812 (0.750, 0.874)	0.865 (0.816, 0.914)	6.55%	**0.010**
**DT model**				
Age&Sex	0.697 (0.626, 0.768)	0.760 (0.697, 0.824)	9.09%	**0.012**
Aspelund model	0.733 (0.665, 0.800)	0.795 (0.737, 0.853)	8.52%	**0.010**
Hippisley model	0.739 (0.670, 0.808)	0.793 (0.734, 0.852)	7.33%	**0.011**
Dagliati model	0.743 (0.676, 0.809)	0.803 (0.747, 0.859)	8.13%	**0.008**
ISDR model	0.733 (0.665, 0.801)	0.796 (0.739, 0.853)	8.53%	**0.009**
JDC model	0.748 (0.681, 0.815)	0.805 (0.746, 0.864)	7.64%	**0.009**
Tarasewicz model	0.760 (0.694, 0.827)	0.820 (0.765, 0.876)	7.89%	**0.005**
All model	0.812 (0.750, 0.874)	0.845 (0.793, 0.898)	4.13%	**0.039**

**P* values were calculated using DeLong's test for paired comparisons of C-indices between the benchmark model and the corresponding model incorporating Pro-DRN.

Pro-DRN, proteome-based diabetic retinal neurodegeneration; CI, confidence interval; RF, random forest; NN, neural network; KNN, K-Nearest Neighbors; SVM, support vector machine; DT, decision tree.

https://doi.org/10.1371/journal.pmed.1004868.t002

(range 21.2% to 24.5%) and NRI by 38.7% (range 22.6% to 45.2%), with the All model showing IDI = 18.3% (95% CI [0.120, 0.248]; $P<0.001$) and NRI = 35.5% (95% CI [0.119, 0.593]; $P=0.008$; Table 3).

To evaluate robustness across algorithms, Pro-DRN was reconstructed using seven alternative ML methods. All versions consistently improved discrimination and reclassification. For instance, LightGBM achieved ΔC-index values ranging from 0.113 to 0.136 (best C-index = 0.891; 95% CI [0.850, 0.932]), with IDI = 17.9% and NRI = 38.7%. Random Forest yielded ΔC-index values ranging from 0.115 to 0.137 (best C-index = 0.897; 95% CI [0.856–0.937]), with IDI = 21.6% and NRI = 29.6% (Tables 2 and 3). These consistent improvements across diverse algorithms underscore the reproducibility, generalizability, and robustness of proteomic markers for enhancing DRN risk stratification.

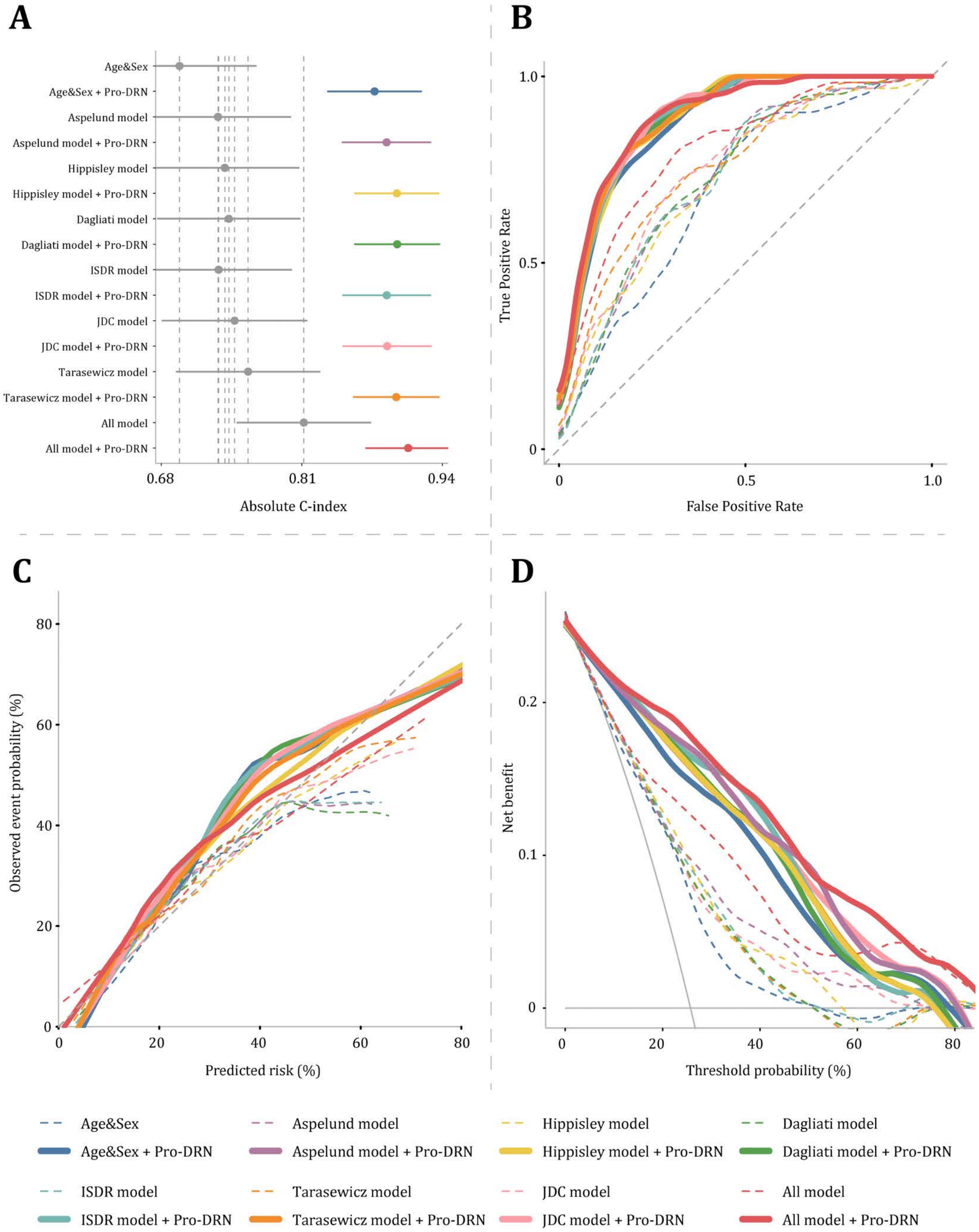

Fig 5. Pro-DRN enhances conventional DRN prediction models. (A) Discrimination for eight clinical frameworks with and without the proteomics score (Pro-DRN). Gray points and horizontal bars denote the original models with 95% CIs; colored points indicate models after adding Pro-DRN. Across frameworks, Pro-DRN consistently improves the C-index, with the "All model + Pro-DRN" performing best. **(B)** ROC curves. Solid lines show models with Pro-DRN and dashed lines the corresponding clinical models. Pro-DRN shifts the curves upward and left. **(C)** Calibration (observed event probability vs. predicted risk). Models augmented with Pro-DRN (solid) align more closely with the 45° reference line than their baselines (dashed). **(D)** Decision curve analysis. Net benefit across threshold probabilities is higher after adding Pro-DRN over a broad range of thresholds.

https://doi.org/10.1371/journal.pmed.1004868.g005

Table 3. Reclassification and discrimination gains with Pro-DRN across established models.

	IDI (95% CI)	*P* value*	NRI (95% CI)	*P* value*
**XGboost model**				
Age&Sex	0.242 (0.170, 0.316)	**<0.001**	0.419 (0.180, 0.643)	**<0.001**
Aspelund model	0.241 (0.170, 0.314)	**<0.001**	0.452 (0.222, 0.673)	**<0.001**
Hippisley model	0.245 (0.177, 0.318)	**<0.001**	0.452 (0.222, 0.673)	**<0.001**
Dagliati model	0.242 (0.175, 0.315)	**<0.001**	0.355 (0.123, 0.593)	**0.004**
ISDR model	0.240 (0.170, 0.312)	**<0.001**	0.452 (0.222, 0.673)	**<0.001**
JDC model	0.212 (0.145, 0.285)	**<0.001**	0.226 (0.030, 0.467)	**0.028**
Tarasewicz model	0.216 (0.147, 0.291)	**<0.001**	0.387 (0.147, 0.607)	**0.002**
All model	0.183 (0.120, 0.248)	**<0.001**	0.355 (0.119, 0.593)	**0.008**
**LightGBM model**				
Age&Sex	0.195 (0.130, 0.258)	**<0.001**	0.290 (0.040, 0.524)	**0.030**
Aspelund model	0.185 (0.119, 0.245)	**<0.001**	0.387 (0.129, 0.618)	**0.002**
Hippisley model	0.193 (0.123, 0.254)	**<0.001**	0.452 (0.200, 0.672)	**<0.001**
Dagliati model	0.193 (0.128, 0.253)	**<0.001**	0.484 (0.250, 0.683)	**<0.001**
ISDR model	0.186 (0.120, 0.246)	**<0.001**	0.387 (0.129, 0.618)	**0.002**
JDC model	0.156 (0.096, 0.211)	**<0.001**	0.323 (0.085, 0.541)	**0.008**
Tarasewicz model	0.165 (0.109, 0.230)	**<0.001**	0.290 (0.064, 0.539)	**0.018**
All model	0.135 (0.087, 0.190)	**<0.001**	0.419 (0.193, 0.652)	**0.002**
**RF model**				
Age&Sex	0.222 (0.150, 0.292)	**<0.001**	0.323 (0.079, 0.545)	**0.014**
Aspelund model	0.224 (0.151, 0.293)	**<0.001**	0.290 (0.059, 0.514)	**0.022**
Hippisley model	0.227 (0.154, 0.293)	**<0.001**	0.258 (0.015, 0.491)	**0.048**
Dagliati model	0.228 (0.157, 0.297)	**<0.001**	0.323 (0.079, 0.545)	**0.014**
ISDR model	0.224 (0.150, 0.293)	**<0.001**	0.290 (0.059, 0.514)	**0.022**
JDC model	0.195 (0.125, 0.260)	**<0.001**	0.323 (0.068, 0.548)	**0.016**
Tarasewicz model	0.198 (0.133, 0.273)	**<0.001**	0.290 (0.051, 0.539)	**0.018**
All model	0.166 (0.105, 0.232)	**<0.001**	0.258 (0.014, 0.500)	**0.046**
**NN model**				
Age&Sex	0.143 (0.086, 0.196)	**<0.001**	0.258 (0.000, 0.491)	0.052
Aspelund model	0.136 (0.080, 0.189)	**<0.001**	0.226 (−0.030, 0.469)	0.090
Hippisley model	0.142 (0.087, 0.196)	**<0.001**	0.226 (−0.030, 0.469)	0.090
Dagliati model	0.144 (0.089, 0.199)	**<0.001**	0.290 (0.033, 0.525)	**0.036**
ISDR model	0.143 (0.084, 0.197)	**<0.001**	0.290 (0.019, 0.527)	**0.034**
JDC model	0.115 (0.061, 0.166)	**<0.001**	0.323 (0.071, 0.556)	**0.016**
Tarasewicz model	0.121 (0.070, 0.177)	**<0.001**	0.258 (0.017, 0.524)	**0.032**
All model	0.092 (0.047, 0.144)	**<0.001**	0.226 (0.000, 0.491)	0.074
**Logistic model**				
Age&Sex	0.182 (0.120, 0.249)	**<0.001**	0.323 (0.094, 0.541)	**<0.001**
Aspelund model	0.178 (0.118, 0.244)	**<0.001**	0.355 (0.125, 0.568)	**<0.001**
Hippisley model	0.179 (0.119, 0.245)	**<0.001**	0.290 (0.053, 0.508)	**0.014**
Dagliati model	0.184 (0.123, 0.251)	**<0.001**	0.355 (0.129, 0.574)	**<0.001**
ISDR model	0.184 (0.122, 0.250)	**<0.001**	0.355 (0.123, 0.571)	**<0.001**
JDC model	0.154 (0.096, 0.212)	**<0.001**	0.258 (0.016, 0.483)	**0.040**
Tarasewicz model	0.154 (0.099, 0.215)	**<0.001**	0.258 (0.032, 0.510)	**0.036**
All model	0.129 (0.076, 0.189)	**<0.001**	0.290 (0.069, 0.531)	**0.012**

(Continued)

Table 3. (Continued)

	IDI (95% CI)	*P* value*	NRI (95% CI)	*P* value*
**KNN model**				
Age&Sex	0.140 (0.084, 0.200)	**<0.001**	0.161 (−0.100, 0.417)	0.234
Aspelund model	0.133 (0.078, 0.190)	**<0.001**	0.161 (−0.100, 0.412)	0.240
Hippisley model	0.144 (0.087, 0.200)	**<0.001**	0.194 (−0.063, 0.444)	0.152
Dagliati model	0.133 (0.079, 0.189)	**<0.001**	0.161 (−0.091, 0.404)	0.230
ISDR model	0.134 (0.078, 0.191)	**<0.001**	0.194 (−0.069, 0.440)	0.182
JDC model	0.107 (0.057, 0.158)	**<0.001**	0.161 (−0.085, 0.397)	0.200
Tarasewicz model	0.105 (0.057, 0.156)	**<0.001**	0.161 (−0.063, 0.433)	0.210
All model	0.087 (0.042, 0.137)	**<0.001**	0.226 (0.000, 0.490)	0.068
**SVM model**				
Age&Sex	0.124 (0.073, 0.174)	**<0.001**	0.161 (−0.075, 0.393)	0.242
Aspelund model	0.118 (0.068, 0.167)	**<0.001**	0.194 (−0.057, 0.429)	0.150
Hippisley model	0.125 (0.073, 0.176)	**<0.001**	0.258 (0.014, 0.482)	**0.046**
Dagliati model	0.123 (0.071, 0.176)	**<0.001**	0.226 (−0.029, 0.458)	0.084
ISDR model	0.119 (0.069, 0.169)	**<0.001**	0.129 (−0.108, 0.367)	0.378
JDC model	0.092 (0.049, 0.135)	**<0.001**	0.226 (−0.016, 0.465)	0.084
Tarasewicz model	0.092 (0.047, 0.137)	**<0.001**	0.161 (−0.071, 0.410)	0.196
All model	0.073 (0.036, 0.114)	**0.002**	0.258 (0.019, 0.500)	**0.038**
**DT model**				
Age&Sex	0.064 (0.026, 0.106)	**<0.001**	−0.097 (−0.334, 0.161)	0.480
Aspelund model	0.061 (0.024, 0.102)	**0.002**	−0.097 (−0.334, 0.161)	0.480
Hippisley model	0.062 (0.025, 0.104)	**0.004**	−0.065 (−0.300, 0.194)	0.656
Dagliati model	0.065 (0.028, 0.105)	**<0.001**	−0.097 (−0.334, 0.161)	0.480
ISDR model	0.061 (0.024, 0.102)	**0.004**	−0.097 (−0.334, 0.161)	0.480
JDC model	0.054 (0.020, 0.092)	**0.002**	−0.065 (−0.300, 0.208)	0.654
Tarasewicz model	0.056 (0.017, 0.095)	**0.002**	−0.065 (−0.310, 0.192)	0.646
All model	0.050 (0.013, 0.090)	**0.012**	0.000 (−0.236, 0.262)	1.046

**P* values for IDI and NRI were estimated by bootstrap resampling.

Pro-DRN, proteome-based diabetic retinal neurodegeneration; IDI, integrated discrimination improvement; NRI, net reclassification improvement; CI, Confidence interval; RF, random forest; NN, neural network; KNN, K-Nearest Neighbors; SVM, support vector machine; DT, decision tree.

https://doi.org/10.1371/journal.pmed.1004868.t003

Pro-DRN demonstrates excellent calibration and clinical utility

Beyond discrimination and reclassification, we systematically evaluated Pro-DRN's calibration and clinical utility (Fig 5C and 5D). Calibration plots showed that all conventional models incorporating Pro-DRN achieved markedly improved agreement between predicted and observed DRN risk, with curves closely approaching the ideal diagonal and substantially reducing systematic bias, particularly in mid-to-high risk ranges (Fig 5C). Decision curve analysis (DCA) further demonstrated that models with Pro-DRN consistently delivered higher net clinical benefit across relevant threshold probabilities, outperforming both the original clinical models and extreme strategies of "treat all" or "treat none." Notably, the fully adjusted model combined with Pro-DRN achieved the greatest net benefit across most thresholds (Fig 5D). These results indicate that Pro-DRN substantially enhances calibration and clinical utility, offering a robust, interpretable, and actionable tool for early identification and targeted intervention in individuals at high risk of DRN.

Robustness Analyses of Pro-DRN Integrated Models

To further stress-test the benchmarks, we additionally augmented each conventional model with measures of insulin resistance (fasting insulin, HOMA2-IR, TyG), beta cell function (fasting C-peptide, HOMA2-B), and glycemic indices (fasting glucose, HbA1c); Pro-DRN remained consistently additive across all augmented baselines (S8 Table).

We tested robustness using three alternative DRN definitions (DRN-Top10Slope, DRN-ThinLast and DRN-ExcessLoss). Pro-DRN preserved discrimination across all definitions (C-index 0.880, 0.753 and 0.848, respectively; S9 Table) and consistently improved the fully adjusted clinical framework (All model: 0.856 to 0.935; 0.738 to 0.832; and 0.823 to 0.902; S10 Table).

To establish the clinical relevance of Pro-DRN beyond its OCT-based definition, we examined whether the baseline Pro-DRN score was associated with subsequent patient-centered outcomes. Higher Pro-DRN was associated with subsequent clinically meaningful vision impairment defined by 15 or more ETDRS letters loss (OR = 27.98; 95% CI [1.07, 578.13]; $P = 0.036$), and was also associated with diabetic peripheral neuropathy (OR = 5.82; 95% CI [1.41, 22.97]; $P = 0.013$) as defined by the Michigan Neuropathy Screening Instrument patient questionnaire (Table 4). These findings support the broader clinical relevance of Pro-DRN, although they should be interpreted cautiously given the limited number of events and the wide confidence interval.

Shiny-based online tool for clinical translation

To accelerate clinical translation, we deployed the locked Pro-DRN model as an interactive, web-based decision-support tool (https://fmb-gdes2025.shinyapps.io/DRN_predict/). The tool comprises two core modules: (1) Pro-DRN score calculation: users can upload standardized measurements of DRN-associated plasma proteins, and the system computes the Pro-DRN score in real time using the XGBoost model developed in this study; (2) DRN risk prediction: based on the calculated score, the tool generates individualized risk estimates with dynamic visual feedback. The interface supports real-time parameter linkage, input validation, and continuous risk updating as data change. An intuitive risk-stratification display further aids clinical interpretation. Designed with a "left input–right output" layout, the platform enables rapid, reliable risk assessment and documentation, making it suitable for clinical consultations, screening programs, and longitudinal follow-up.

Cross-ethnic external validation in UKB-PPP

To assess generalizability and robustness, we performed cross-ethnic external validation in the independent cohort. Despite limited longitudinal proteome–imaging data and platform differences, we systematically examined cross-sectional associations between plasma proteins and macular RNFL thickness using standardized covariate adjustment and FDR correction (Table 5). In univariate analyses, 52 of 71 proteins (73.2%) were significantly negatively associated with RNFL thickness, with β values ranging from −0.841 μm (95% CI [−1.206, −0.476]) to −0.337 μm (95% CI [−0.618, −0.056]) per 1–SD increase. UMOD was the sole protein with a positive association ($\beta = 0.488$; 95% CI [0.171, 0.805]). In multivariable models adjusting for confounders and multiple testing, 34 proteins retained significant negative associations,

Table 4. Association of the Pro-DRN score with vision impairment and diabetic peripheral neuropathy.

Outcome	OR (95% CI)	*P* value*
Vision impairment	27.98 (1.07, 578.13)	**0.036**
Diabetic peripheral neuropathy	5.82 (1.41, 22.97)	**0.013**

**P* values were obtained from logistic regression models with Wald tests.

OR, odds ratios; CI, confidence intervals.

https://doi.org/10.1371/journal.pmed.1004868.t004

Table 5. Associations of proteins with retinal nerve fiber layer thickness in UK Biobank.

	Univariable Regression			Multivariable Regression		
**Proteins***	**β (95% CI) †**	***P* value**	**P_{FDR} value‡**	**β (95% CI)†**	***P* value**	**P_{FDR} value‡**
DEFA1	−0.723 (−1.062, −0.383)	**3.59×10^{-05}**	**2.35×10^{-04}**	−0.668 (−1.014, −0.322)	**1.73×10^{-04}**	**0.004**
CD14	−0.586 (−0.929, −0.244)	**8.67×10^{-04}**	**0.002**	−0.716 (−1.077, −0.355)	**1.16×10^{-04}**	**0.004**
ESAM	−0.823 (−1.141, −0.504)	**5.79×10^{-07}**	**4.17×10^{-05}**	−0.696 (−1.036, −0.356)	**6.89×10^{-05}**	**0.004**
EPHB4	−0.762 (−1.096, −0.428)	**9.54×10^{-06}**	**1.47×10^{-04}**	−0.639 (−0.983, −0.294)	**3.09×10^{-04}**	**0.006**
CD93	−0.729 (−1.088, −0.370)	**7.96×10^{-05}**	**3.62×10^{-04}**	−0.671 (−1.041, −0.301)	**4.22×10^{-04}**	**0.006**
PI3	−0.660 (−0.974, −0.345)	**4.63×10^{-05}**	**2.78×10^{-04}**	−0.556 (−0.883, −0.228)	**0.001**	**0.008**
CD46	−0.679 (−1.020, −0.337)	**1.11×10^{-04}**	**3.81×10^{-04}**	−0.590 (−0.937, −0.243)	**0.001**	**0.008**
RNASET2	−0.703 (−1.019, −0.387)	**1.63×10^{-05}**	**1.47×10^{-04}**	−0.558 (−0.889, −0.227)	**0.001**	**0.008**
EFEMP1	−0.687 (−1.017, −0.356)	**5.43×10^{-05}**	**2.79×10^{-04}**	−0.589 (−0.943, −0.236)	**0.001**	**0.008**
CCL15	−0.599 (−0.903, −0.294)	**1.32×10^{-04}**	**4.14×10^{-04}**	−0.525 (−0.838, −0.211)	**0.001**	**0.008**
CCN3	−0.750 (−1.085, −0.414)	**1.46×10^{-05}**	**1.47×10^{-04}**	−0.619 (−0.974, −0.265)	**0.001**	**0.008**
NPDC1	−0.682 (−1.021, −0.343)	**9.15×10^{-05}**	**3.81×10^{-04}**	−0.578 (−0.934, −0.222)	**0.002**	**0.009**
TIMP1	−0.714 (−1.032, −0.396)	**1.34×10^{-05}**	**1.47×10^{-04}**	−0.543 (−0.878, −0.208)	**0.002**	**0.009**
CCL16	−0.679 (−0.993, −0.364)	**2.83×10^{-05}**	**2.04×10^{-04}**	−0.524 (−0.855, −0.193)	**0.002**	**0.010**
GDF15	−0.724 (−1.023, −0.426)	**2.60×10^{-06}**	**9.35×10^{-05}**	−0.568 (−0.926, −0.209)	**0.002**	**0.010**
COL6A3	−0.679 (−1.013, −0.344)	**8.04×10^{-05}**	**3.62×10^{-04}**	−0.522 (−0.865, −0.178)	**0.003**	**0.013**
CD59	−0.795 (−1.145, −0.444)	**1.12×10^{-05}**	**1.47×10^{-04}**	−0.571 (−0.947, −0.196)	**0.003**	**0.013**
ACTA2	−0.841 (−1.206, −0.476)	**7.99×10^{-06}**	**1.47×10^{-04}**	−0.619 (−1.034, −0.204)	**0.004**	**0.013**
SPON2	−0.630 (−0.982, −0.277)	**5.07×10^{-04}**	**0.001**	−0.534 (−0.889, −0.179)	**0.003**	**0.013**
NTproBNP	−0.589 (−0.901, −0.278)	**2.31×10^{-04}**	**0.001**	−0.498 (−0.832, −0.165)	**0.004**	**0.013**
NECTIN2	−0.697 (−1.050, −0.345)	**1.20×10^{-04}**	**3.92×10^{-04}**	−0.527 (−0.893, −0.160)	**0.005**	**0.015**
HSPG2	−0.630 (−0.947, −0.314)	**1.10×10^{-04}**	**3.81×10^{-04}**	−0.479 (−0.808, −0.150)	**0.004**	**0.015**
PTGDS	−0.754 (−1.099, −0.410)	**2.18×10^{-05}**	**1.74×10^{-04}**	−0.536 (−0.908, −0.164)	**0.005**	**0.015**
COL18A1	−0.661 (−0.992, −0.329)	**1.07×10^{-04}**	**3.81×10^{-04}**	−0.514 (−0.870, −0.159)	**0.005**	**0.015**
GPR37	−0.636 (−0.962, −0.311)	**1.47×10^{-04}**	**4.40×10^{-04}**	−0.462 (−0.804, −0.121)	**0.008**	**0.023**
LGALS1	−0.605 (−0.959, −0.251)	**8.64×10^{-04}**	**0.002**	−0.486 (−0.843, −0.128)	**0.008**	**0.023**
PAM	−0.463 (−0.809, −0.116)	**0.009**	**0.016**	−0.466 (−0.814, −0.119)	**0.009**	**0.023**
TFF3	−0.808 (−1.275, −0.341)	**7.54×10^{-04}**	**0.002**	−0.658 (−1.150, −0.166)	**0.009**	**0.023**
CST3	−0.697 (−1.032, −0.362)	**5.37×10^{-05}**	**2.79×10^{-04}**	−0.482 (−0.844, −0.119)	**0.010**	**0.024**
LCN2	−0.506 (−0.829, −0.183)	**0.002**	**0.004**	−0.445 (−0.791, −0.099)	**0.012**	**0.029**
PLIN3	−0.579 (−0.947, −0.210)	**0.002**	**0.004**	−0.479 (−0.852, −0.105)	**0.012**	**0.029**
CGREF1	−0.527 (−0.840, −0.214)	**0.001**	**0.002**	−0.399 (−0.717, −0.082)	**0.014**	**0.031**
CDH1	−0.613 (−0.995, −0.232)	**0.002**	**0.004**	−0.485 (−0.872, −0.098)	**0.014**	**0.031**
VCAM1	−0.513 (−0.830, −0.196)	**0.002**	**0.004**	−0.417 (−0.750, −0.084)	**0.015**	**0.031**
SEMA3F	−0.515 (−0.842, −0.188)	**0.002**	**0.004**	−0.385 (−0.721, −0.048)	**0.026**	0.053
TINAGL1	−0.427 (−0.776, −0.078)	**0.017**	**0.025**	−0.405 (−0.765, −0.044)	**0.028**	0.055
TNF	−0.479 (−0.803, −0.154)	**0.004**	**0.007**	−0.371 (−0.700, −0.041)	**0.028**	0.055
MCFD2	−0.428 (−0.755, −0.101)	**0.011**	**0.017**	−0.356 (−0.685, −0.027)	**0.034**	0.063
NOTCH3	−0.268 (−0.586, 0.051)	0.100	0.120	−0.372 (−0.716, −0.027)	**0.035**	0.063
SCARF1	−0.407 (−0.741, −0.072)	**0.018**	**0.026**	−0.368 (−0.706, −0.030)	**0.034**	0.063
PPP1R2	−0.448 (−0.806, −0.090)	**0.014**	**0.022**	−0.376 (−0.736, −0.016)	**0.041**	0.072
IGFBP6	−0.665 (−0.998, −0.331)	**1.07×10^{-04}**	**3.81×10^{-04}**	−0.408 (−0.801, −0.015)	**0.042**	0.072
CCL14	−0.376 (−0.704, −0.048)	**0.025**	**0.035**	−0.338 (−0.668, −0.009)	**0.045**	0.075
ANGPTL1	−0.259 (−0.586, 0.068)	0.122	0.137	−0.331 (−0.665, 0.003)	0.053	0.087

(Continued)

Table 5. (Continued)

	Univariable Regression			Multivariable Regression		
**Proteins***	**β (95% CI) †**	***P* value**	**P_{FDR} value‡**	**β (95% CI)†**	***P* value**	**P_{FDR} value‡**
XG	−0.024 (−0.361, 0.314)	0.891	0.891	−0.461 (−0.931, 0.008)	0.055	0.088
CTSZ	−0.417 (−0.725, −0.109)	**0.008**	**0.015**	−0.303 (−0.613, 0.008)	0.056	0.088
UMOD	0.488 (0.171, 0.805)	**0.003**	**0.005**	0.322 (−0.009, 0.653)	0.057	0.088
FAM3C	−0.571 (−0.905, −0.236)	**0.001**	**0.002**	−0.337 (−0.694, 0.019)	0.065	0.092
CLEC1A	−0.421 (−0.754, −0.087)	**0.014**	**0.022**	−0.316 (−0.651, 0.019)	0.065	0.092
PRTN3	−0.319 (−0.648, 0.010)	0.058	0.075	−0.313 (−0.643, 0.016)	0.063	0.092
THBD	−0.563 (−0.915, −0.210)	**0.002**	**0.004**	−0.349 (−0.716, 0.017)	0.062	0.092
IL2RA	−0.445 (−0.781, −0.109)	**0.010**	**0.016**	−0.320 (−0.665, 0.024)	0.069	0.096
PRSS2	−0.337 (−0.618, −0.056)	**0.019**	**0.028**	−0.260 (−0.543, 0.024)	0.073	0.099
CLEC5A	−0.266 (−0.598, 0.067)	0.118	0.135	−0.315 (−0.671, 0.042)	0.085	0.113
ART3	−0.419 (−0.730, −0.109)	**0.008**	**0.015**	−0.293 (−0.630, 0.043)	0.088	0.115
RARRES2	−0.374 (−0.733, −0.015)	**0.041**	0.055	−0.284 (−0.653, 0.086)	0.133	0.169
CXCL8	−0.386 (−0.721, −0.051)	**0.024**	**0.034**	−0.266 (−0.612, 0.081)	0.133	0.169
IGFBP2	−0.288 (−0.587, 0.011)	0.059	0.075	−0.238 (−0.557, 0.080)	0.143	0.177
RETN	−0.256 (−0.573, 0.061)	0.114	0.132	−0.228 (−0.552, 0.096)	0.168	0.205
IL19	−0.300 (−0.638, 0.038)	0.083	0.101	−0.229 (−0.568, 0.110)	0.186	0.223
CTSL	−0.378 (−0.717, −0.040)	**0.029**	**0.039**	−0.223 (−0.570, 0.124)	0.208	0.246
MFAP5	−0.431 (−0.773, −0.090)	**0.014**	**0.022**	−0.226 (−0.582, 0.129)	0.213	0.248
PDGFRA	−0.279 (−0.617, 0.059)	0.107	0.126	−0.189 (−0.530, 0.151)	0.276	0.310
DKK3	−0.320 (−0.649, 0.010)	0.058	0.075	−0.192 (−0.533, 0.150)	0.272	0.310
REG1A	−0.300 (−0.634, 0.034)	0.079	0.098	−0.187 (−0.527, 0.153)	0.282	0.313
ROR1	−0.258 (−0.597, 0.081)	0.136	0.151	−0.184 (−0.534, 0.166)	0.304	0.331
REG3A	−0.193 (−0.524, 0.138)	0.253	0.272	−0.147 (−0.485, 0.192)	0.397	0.426
CA4	−0.128 (−0.422, 0.166)	0.394	0.411	−0.121 (−0.433, 0.191)	0.447	0.473
CCL27	−0.046 (−0.364, 0.272)	0.776	0.787	0.125 (−0.206, 0.455)	0.459	0.479
CLC	−0.218 (−0.521, 0.086)	0.161	0.175	−0.095 (−0.400, 0.210)	0.542	0.558
REG1B	−0.169 (−0.488, 0.150)	0.299	0.317	−0.035 (−0.360, 0.290)	0.832	0.844
GRK5	−0.073 (−0.394, 0.247)	0.654	0.673	−0.021 (−0.340, 0.298)	0.899	0.899

*Adjusted for age, sex, smoking, systolic blood pressure, HbA1c, and duration of diabetes.

†Per-SD change of retinal nerve fiber layer thickness.

‡Adjusted for multiple testing (Benjamini-Hochberg procedure).

CI, confidence interval.

https://doi.org/10.1371/journal.pmed.1004868.t005

fully concordant with GDES-PPP findings, including key markers such as ACTA2, COL6A3, CD46, RNASET2, CCL15, NT-proBNP, NECTIN2, and HSPG2, with *β* values ranging from −0.716 μm (95% CI [−1.077, −0.355]) to −0.399 μm (95% CI [−0.717, −0.082]). UMOD maintained a protective trend (*β* = 0.322), though not reaching significance (P = 0.088). Core GDES protein signatures are reproducible across populations, with consistent effects and biological relevance, highlighting their robust translational potential for DRN.

We further performed model-level external validation in UKB-PPP using a cross-sectional thin-RNFL phenotype (thinnest quartile of baseline pRNFL thickness). Adding Pro-DRN consistently improved discrimination across all conventional frameworks (e.g., Age and Sex: 0.635 to 0.714; All model: 0.676 to 0.756; S11 Table) and improved calibration as assessed by lower Brier scores and no evidence of lack of fit by the Hosmer–Lemeshow test (S12 Table).

Discussion

Over the past two decades, DRD has been reconceptualised from a purely microvascular disorder to a neurovascular unit (NVU) disease, in which DRN emerges as an early and independent driver of disease progression [3,50]. By integrating high-throughput plasma proteomics with longitudinal multimodal retinal imaging, we systematically identified 71 plasma proteins significantly associated with DRN, predominantly involved in inflammation and immune responses, extracellular matrix remodeling, and microcirculatory homeostasis. Leveraging these molecular features, we constructed an AI-based predictive model, Pro-DRN, which demonstrated excellent discrimination that further improved when combined with clinical variables, substantially outperforming conventional risk factors. External validation in the UK Biobank confirmed these associations, demonstrating robustness across populations. An online platform was developed to facilitate clinical translation, enabling precise early risk assessment and neuroprotective intervention before irreversible retinal damage occurs. To our knowledge, this is among the earliest multi-cohort efforts to demonstrate the predictive value of plasma proteomics for DRN.

Unlike earlier proteomic studies, which have focused largely on late-stage vascular lesions and have typically drawn on intraocular fluids or animal models, this study extends the field in three complementary ways [17,29–31,51–53]. First, it prospectively targets a longitudinal neurodegenerative phenotype (OCT-quantified RNFL thinning rate). Second, it applies large-scale plasma proteomics to detect systemic molecular signals amenable to clinical implementation. Third, it integrates a multi-algorithm ML framework to handle complex nonlinear relationships in high-dimensional data, uncovering subtle yet reproducible signals that traditional methods may miss. Taken together, these choices reposition DRN from a condition recognized late at the imaging level to one that can be stratified early and mechanism-guided at the molecular level.

The Pro-DRN model, trained exclusively on proteomic features, achieved a C-index of 0.860, outperforming conventional models based on clinical variables (C-index 0.50–0.69). Combining proteomic data with clinical parameters further enhanced predictive performance to a C-index of 0.908, highlighting the independent and additive value of proteomic biomarkers. These findings align with growing evidence that non-traditional biomarkers, particularly proteomic signatures, can improve risk stratification by detecting early pathophysiological perturbations, such as neurovascular unit dysfunction, before irreversible structural damage occurs. Comparable gains have been reported in proteomic prediction of diabetic nephropathy and retinopathy, where inflammation- and tissue-injury-related proteins consistently outperform routine clinical metrics. These results argue for incorporating plasma proteomic profiling into precision prognostics for diabetic neurodegenerative complications, enabling the early identification of high-risk individuals and the timely delivery of targeted interventions before overt disease progression.

ML and AI served a dual purpose in this work: it modeled the high-dimensional proteomic data and it stress-tested the robustness of the identified signals. We benchmarked eight algorithms spanning ensemble gradient boosting, bagging, neural networks, and classical linear and instance-based learners within a unified cross-validation framework. Despite their very different theoretical foundations, every algorithm yielded concordant gains in discrimination, indicating that the proteomic signal rather than any particular modeling choice drives the predictive performance of Pro-DRN. Tree-based models, particularly XGBoost, excelled at capturing complex relationships between proteomic and imaging features due to their capacity for high-order interactions and minimal distributional assumptions [54–57]. This algorithmic concordance establishes that the plasma proteomic signatures of DRN are stable and generalizable, providing a secure foundation for biological interpretation and clinical translation.

Cross-cohort external validation is indispensable for establishing the robustness of candidate biomarkers and the generalisability of risk prediction models [58]. Using the UKB-PPP cohort, which differs substantially in genetic background, lifestyle, and epidemiology, we successfully reproduced the associations of core plasma proteins with DRN. Both the univariate and the multivariable analyses recapitulated the direction and magnitude of association for key markers, including ACTA2, COL6A3, and HSPG2, indicating that these biomarkers reflect intrinsic biological features of DRN rather than population- or platform-specific phenomena.

To address the "black box" nature of AI-based predictions, we applied SHAP (Shapley Additive Explanations), which decomposes each prediction into quantitative, signed contributions from every protein. Proteins such as COL6A3, ACTA2, HSPG2, CCL16, REG3A, PAM, ART3, and TIMP1 jointly formed the primary positive risk contributions. These analyses revealed that the Pro-DRN score is governed by the cumulative and interactive contributions of multiple proteins rather than by any single dominant marker. Integrating these findings with functional enrichment analyses, the signals can be grouped into four core pathological axes: (1) inflammation and immune recruitment (e.g., CCL16), (2) basement membrane and extracellular matrix remodeling (e.g., COL6A3, HSPG2), (3) membrane-receptor function and blood–retina barrier (BRB) integrity (e.g., HSPG2), and (4) microcirculatory homeostasis (e.g., ACTA2). These pathways align with prior hypotheses regarding pericyte loss, BRB disruption, and glial activation, collectively highlighting NVU instability as a central molecular driver of early DRN [1,3,7,59,60].

Key proteins illuminate DRN pathology. Elevated ACTA2 signals phenotypic transformation of retinal pericytes or smooth muscle-like cells, with ischemic stress triggering sustained contraction, capillary spasm, hypoperfusion, axonal transport disruption, metabolic stress, and glial activation, accelerating neurodegeneration [61]. COL6A3, a major extracellular matrix and basement membrane protein, undergoes diabetic remodeling that alters matrix mechanics, permeability, bioactive factor release, and axonal support, destabilizing the neuronal microenvironment [62,63]. HSPG2 regulates vascular permeability and barrier integrity, linking circulating protein signals to RNFL thinning [64]. Collectively, microvascular dysfunction, matrix remodeling, and BRB disruption form an interconnected network whose feedback loops drive DRN through NVU instability.

Among the candidate proteins, uromodulin (UMOD) stood out for its protective association with retinal nerve fiber layer preservation. Classically regarded as a kidney-derived protein secreted by distal tubular epithelial cells, UMOD is known to regulate ion transport, innate immunity, and epithelial barrier function [65,66]. Our findings implicate UMOD in DRN and suggest a "kidney–vasculature–retina axis" that modulates retinal neuronal vulnerability [67,68]. In diabetes, renal impairment amplifies inflammation, metabolic dysregulation, and endothelial dysfunction, destabilizing the NVU and accelerating glial activation and microcirculatory injury [69]. The protective association observed here likely reflects the systemic homeostatic role of healthy renal tubules, which confers resistance to the inflammatory cascades and endothelial dysfunction that precipitate neurovascular injury. Clinically, diabetic nephropathy and retinopathy often coexist and correlate in severity, suggesting that preserving tubular health may also protect retinal neurons, offering a cross-organ strategy for early prevention of diabetic complications [68,70–72].

The retina, as an embryological outgrowth of the diencephalon, offers an accessible window onto central nervous system biology. Substantial evidence indicates that diabetic patients face elevated risks of cognitive impairment and Alzheimer's disease (AD), with early pathological changes often mirrored in the retina [73–76]. For example, inner retinal thinning correlates with mild cognitive impairment, and multifocal ERG abnormalities can predict future cognitive decline [9,10,77,78]. Notably, several DRN-risk proteins identified here (e.g., COL6A3, HSPG2, TIMP1) have also been implicated in AD and Parkinson's disease, suggesting shared molecular underpinnings between retinal and central neurodegenerative disorders [14,15,20,22,79,80]. DRN should therefore be considered not merely as a localized complication of diabetes but as a potential early biomarker of systemic neurodegeneration. Future studies should prospectively evaluate whether these protein markers jointly predict DRN and cognitive decline, enabling development of blood-based, cross-disease early-warning systems [18].

These findings carry several clinical translational implications. First, Pro-DRN provides a quantitative tool for early risk stratification, enabling efficient allocation of limited ophthalmic screening and surveillance resources to those who stand to benefit most. High-risk individuals could benefit from intensified surveillance, such as shorter OCT follow-up intervals, and early interventions that complement current guidelines focused on vascular lesions while often neglecting neurodegeneration [45,81–83]. Second, it offers an objective enrichment criterion for selecting participants in neuroprotective trials, including trials of GLP-1 receptor agonists, DPP-4 inhibitors, and pathway-specific investigational agents, which should substantially enhance trial efficiency and statistical power [84,85]. Third, the proteins and pathways implicated by our analyses constitute a set of human-omics-derived candidate targets for the development of novel therapies for DRN.

The strengths of this study include its large-scale prospective design, six-year longitudinal OCT phenotyping, high-throughput plasma proteomics, and rigorous cross-cohort validation. Several limitations should nonetheless be acknowledged. First, circulating plasma protein levels do not fully capture local retinal expression and may therefore incompletely reflect the pathogenic mechanisms operating in retinal tissue. Future studies should integrate spatial proteomics of ocular tissues and employ cellular or animal models to dissect the specific roles and therapeutic potential of candidate molecules in DRN. Second, the RNFL measurement region differed between cohorts, being peripapillary in GDES and macular in UK Biobank; although prior evidence indicates that both regions reliably reflect neurodegeneration, residual non-comparability cannot be excluded. Third, our proteomic measurements were obtained at a single time point, precluding any evaluation of temporal proteomic trajectories or their dynamic relationship with disease progression. Cost-efficient, targeted panels could enable large-scale longitudinal monitoring in future studies. Fourth, while Pro-DRN achieved strong discrimination, predictive performance might be further enhanced by integrating additional layers of data, including genomic risk scores, epigenomic features, wearable-sensor metrics, and environmental exposures. Finally, Olink targeted proteomics, while broad, samples only a subset of the human proteome, so that potentially informative proteins and mechanisms remain unexplored. Advances in ultra–high-throughput proteomics and AI promise more comprehensive molecular characterization of DRN [15,16,18].

In summary, by integrating high-throughput plasma proteomics with multi-algorithm ML and explainable AI, this multi-cohort study identified a robust, cross-population plasma proteomic signature of early DRN. The resulting Pro-DRN model achieved high predictive performance, supported excellent calibration and clinical utility, and translates seamlessly into a deployable online decision-support tool, together marking a shift from late imaging-based recognition to early molecular prediction of DRN. Beyond prediction, these findings illuminate the molecular architecture of neurovascular unit instability in early diabetes and establish a foundation for high-risk screening, neuroprotective trial enrichment, and the rational design of targeted therapies.

Ethics approval

The study was approved by the Northwest Multicenter Research Ethics Committee (11/NW/0382) and the Ethics Committee of Zhongshan Ophthalmic Center (2017KYPJ094).

Supporting information

S1 Checklist. STROBE statement. Based on the STROBE Statement (Strengthening the Reporting of Observational Studies in Epidemiology). Available from: https://www.strobe-statement.org/. The STROBE Statement is distributed under the Creative Commons Attribution 4.0 International License (CC BY 4.0).
(DOCX)

S2 Checklist. TRIPOD checklist. Based on the TRIPOD Statement (Transparent Reporting of a multivariable prediction model for Individual Prognosis Or Diagnosis). Available from: https://www.tripod-statement.org/. The TRIPOD Statement is distributed under the Creative Commons Attribution 4.0 International License (CC BY 4.0).
(DOCX)

S1 Text. Supplementary methods.
(DOCX)

S1 Table. Baseline characteristics of the population 1 (GDES-PPP cross-sectional cohort) and population 3 (UKB-PPP validation cohort).
(DOCX)

S2 Table. Proteins associated with average retinal nerve fiber layer thickness.
(DOCX)

S3 Table. Proteins associated with the thinning rate of retinal nerve fiber layer thickness.
(DOCX)

S4 Table. Proteins associated with the thinning rate of retinal nerve fiber layer thickness in the sensitivity analysis with further adjustment for baseline retinal nerve fiber layer thickness.
(DOCX)

S5 Table. Performance of Pro-DRN for predicting DRN with various ML algorithms in the GDES cohort.
(DOCX)

S6 Table. Comparison of the performance between conventional predictors and Pro-DRN for predicting DRN.
(DOCX)

S7 Table. Comparison of the performance of incorporating Pro-DRN with the full proteins in the GDES.
(DOCX)

S8 Table. Incremental predictive value of Pro-DRN beyond metabolically augmented conventional clinical models.
(DOCX)

S9 Table. Discrimination of Pro-DRN under alternative DRN definitions.
(DOCX)

S10 Table. Incremental discrimination of Pro-DRN beyond conventional clinical frameworks under alternative DRN definitions.
(DOCX)

S11 Table. Model-level external validation of Pro-DRN in UKB-PPP using a cross-sectional thin-RNFL outcome.
(DOCX)

S12 Table. Assessment of model calibration for Pro-DRN in UKB-PPP using a cross-sectional thin-RNFL outcome.
(DOCX)

S13 Table. Predictors used in conventional prediction models.
(DOCX)

S14 Table. Protein names and gene names for proteins identified in this study.
(DOCX)

S1 Data. Supplementary data for Figs 2, 3, and 5.
(XLSX)

Acknowledgments

We sincerely thank all participants of the Guangzhou Diabetic Eye Study (GDES) and the UK Biobank (UKB) for their generous and sustained contributions to this research. We are equally grateful to the ophthalmologists, nurses, optometrists, imaging technicians, laboratory staff, and data managers at Preventive Ophthalmology of Zhongshan Ophthalmic Center who carried out recruitment, clinical examinations, OCT acquisition, sample processing, and data curation across the six-year follow-up. We thank the UK Biobank study team and the participating assessment centers for collection and long-term stewardship of the imaging and proteomic data.

Author contributions

Conceptualization: Huangdong Li, Wenyong Huang, Wei Wang.

Data curation: Huangdong Li, Ziyu Zhu, Shaopeng Yang, Weijing Cheng, Shaoying Tan, Zhuoting Zhu, Shida Chen, Wenyong Huang, Wei Wang.

Formal analysis: Huangdong Li, Ziyu Zhu, Shaopeng Yang, Zhuoyao Xin.

Funding acquisition: Weijing Cheng, Shaoying Tan, Shida Chen, Wei Wang.

Investigation: Wei Wang.

Methodology: Huangdong Li, Ziyu Zhu, Shaopeng Yang, Zhuoyao Xin, Lei Zhang, Wenyong Huang, Wei Wang.

Project administration: Huangdong Li, Weijing Cheng, Wei Wang.

Software: Huangdong Li, Lei Zhang, Zhuoting Zhu.

Supervision: Wenyong Huang, Wei Wang.

Validation: Huangdong Li.

Visualization: Huangdong Li.

Writing – original draft: Huangdong Li, Wei Wang.

Writing – review & editing: Huangdong Li, Wei Wang.

References

1. Abel ED, Gloyn AL, Evans-Molina C, Joseph JJ, Misra S, Pajvani UB, et al. Diabetes mellitus-progress and opportunities in the evolving epidemic. Cell. 2024;187(15):3789–820. https://doi.org/10.1016/j.cell.2024.06.029 PMID: 39059357
2. Suzuki K, Hatzikotoulas K, Southam L, Taylor HJ, Yin X, Lorenz KM, et al. Genetic drivers of heterogeneity in type 2 diabetes pathophysiology. Nature. 2024;627(8003):347–57. https://doi.org/10.1038/s41586-024-07019-6 PMID: 38374256
3. Sivaprasad S, Wong TY, Gardner TW, Sun JK, Bressler NM. Diabetic retinal disease. Nat Rev Dis Primers. 2025;11(1):62. https://doi.org/10.1038/s41572-025-00646-x PMID: 40877334
4. Jampol LM, Glassman AR, Sun J. Evaluation and care of patients with diabetic retinopathy. N Engl J Med. 2020;382(17):1629–37. https://doi.org/10.1056/NEJMra1909637 PMID: 32320570
5. Lim JI, Kim SJ, Bailey ST, Kovach JL, Vemulakonda GA, Ying G, et al. Diabetic retinopathy preferred practice pattern(R). Ophthalmology. 2025;132(4):P75–162. https://doi.org/10.1016/j.ophtha.2024.12.020 PMID: 39918521
6. Yang Q, Yasvoina M, Olvera-Barrios A, Mendes J, Zhu M, Egan C, et al. Deciphering the connection between microvascular damage and neurodegeneration in early diabetic retinopathy. Diabetes. 2024;73(11):1883–94. https://doi.org/10.2337/db24-0107 PMID: 38968415
7. Sohn EH, van Dijk HW, Jiao C, Kok PHB, Jeong W, Demirkaya N, et al. Retinal neurodegeneration may precede microvascular changes characteristic of diabetic retinopathy in diabetes mellitus. Proc Natl Acad Sci U S A. 2016;113(19):E2655-64. https://doi.org/10.1073/pnas.1522014113 PMID: 27114552
8. Zhao B, Li Y, Fan Z, Wu Z, Shu J, Yang X, et al. Eye-brain connections revealed by multimodal retinal and brain imaging genetics. Nat Commun. 2024;15(1):6064. https://doi.org/10.1038/s41467-024-50309-w PMID: 39025851
9. Mutlu U, Colijn JM, Ikram MA, Bonnemaijer PWM, Licher S, Wolters FJ, et al. Association of retinal neurodegeneration on optical coherence tomography with dementia: a population-based study. JAMA Neurol. 2018;75(10):1256–63. https://doi.org/10.1001/jamaneurol.2018.1563 PMID: 29946702
10. Ko F, Muthy ZA, Gallacher J, Sudlow C, Rees G, Yang Q, et al. Association of retinal nerve fiber layer thinning with current and future cognitive decline: a study using optical coherence tomography. JAMA Neurol. 2018;75(10):1198–205. https://doi.org/10.1001/jamaneurol.2018.1578 PMID: 29946685
11. Zekavat SM, Jorshery SD, Rauscher FG, Horn K, Sekimitsu S, Koyama S, et al. Phenome- and genome-wide analyses of retinal optical coherence tomography images identify links between ocular and systemic health. Sci Transl Med. 2024;16(731):eadg4517. https://doi.org/10.1126/scitranslmed.adg4517 PMID: 38266105
12. Zhou Y, Chia MA, Wagner SK, Ayhan MS, Williamson DJ, Struyven RR, et al. A foundation model for generalizable disease detection from retinal images. Nature. 2023;622(7981):156–63. https://doi.org/10.1038/s41586-023-06555-x PMID: 37704728
13. De Clerck EEB, Schouten JSAG, Berendschot TTJM, Kessels AGH, Nuijts RMMA, Beckers HJM, et al. New ophthalmologic imaging techniques for detection and monitoring of neurodegenerative changes in diabetes: a systematic review. Lancet Diabetes Endocrinol. 2015;3(8):653–63. https://doi.org/10.1016/S2213-8587(15)00136-9 PMID: 26184671

14. Deng Y-T, You J, He Y, Zhang Y, Li H-Y, Wu X-R, et al. Atlas of the plasma proteome in health and disease in 53,026 adults. Cell. 2025;188(1):253-271.e7. https://doi.org/10.1016/j.cell.2024.10.045 PMID: 39579765

15. Topol EJ. The revolution in high-throughput proteomics and AI. Science. 2024;385(6716):eads5749. https://doi.org/10.1126/science.ads5749 PMID: 39325883

16. He F, Aebersold R, Baker MS, Bian X, Bo X, Chan DW, et al. π-HuB: the proteomic navigator of the human body. Nature. 2024;636(8042):322–31. https://doi.org/10.1038/s41586-024-08280-5 PMID: 39663494

17. Wolf J, Rasmussen DK, Sun YJ, Vu JT, Wang E, Espinosa C, et al. Liquid-biopsy proteomics combined with AI identifies cellular drivers of eye aging and disease in vivo. Cell. 2023;186(22):4868–84.e12. Epub 2023-10-26. https://doi.org/10.1016/j.cell.2023.09.012 PMID: 37863056

18. Guo T, Steen JA, Mann M. Mass-spectrometry-based proteomics: from single cells to clinical applications. Nature. 2025;638(8052):901–11. https://doi.org/10.1038/s41586-025-08584-0 PMID: 40011722

19. Malmström E, Malmström L, Hauri S, Mohanty T, Scott A, Karlsson C, et al. Human proteome distribution atlas for tissue-specific plasma proteome dynamics. Cell. 2025;188(10):2810-2822.e16. https://doi.org/10.1016/j.cell.2025.03.013 PMID: 40203824

20. Liu W-S, You J, Chen S-D, Zhang Y, Feng J-F, Xu Y-M, et al. Plasma proteomics identify biomarkers and undulating changes of brain aging. Nat Aging. 2025;5(1):99–112. https://doi.org/10.1038/s43587-024-00753-6 PMID: 39653801

21. Argentieri MA, Amin N, Nevado-Holgado AJ, Sproviero W, Collister JA, Keestra SM, et al. Integrating the environmental and genetic architectures of aging and mortality. Nat Med. 2025;31(3):1016–25. https://doi.org/10.1038/s41591-024-03483-9 PMID: 39972219

22. Heo G, Xu Y, Wang E, Ali M, Oh HS-H, Moran-Losada P, et al. Large-scale plasma proteomic profiling unveils diagnostic biomarkers and pathways for Alzheimer's disease. Nat Aging. 2025;5(6):1114–31. https://doi.org/10.1038/s43587-025-00872-8 PMID: 40394224

23. Oh HS-H, Le Guen Y, Rappoport N, Urey DY, Farinas A, Rutledge J, et al. Plasma proteomics links brain and immune system aging with healthspan and longevity. Nat Med. 2025;31(8):2703–11. https://doi.org/10.1038/s41591-025-03798-1 PMID: 40634782

24. Perry AS, Farber-Eger E, Gonzales T, Tanaka T, Robbins JM, Murthy VL, et al. Proteomic analysis of cardiorespiratory fitness for prediction of mortality and multisystem disease risks. Nat Med. 2024;30(6):1711–21. https://doi.org/10.1038/s41591-024-03039-x PMID: 38834850

25. Gadd DA, Hillary RF, Kuncheva Z, Mangelis T, Cheng Y, Dissanayake M, et al. Blood protein assessment of leading incident diseases and mortality in the UK Biobank. Nat Aging. 2024;4(7):939–48. https://doi.org/10.1038/s43587-024-00655-7 PMID: 38987645

26. Argentieri MA, Xiao S, Bennett D, Winchester L, Nevado-Holgado AJ, Ghose U, et al. Proteomic aging clock predicts mortality and risk of common age-related diseases in diverse populations. Nat Med. 2024;30(9):2450–60. https://doi.org/10.1038/s41591-024-03164-7 PMID: 39117878

27. Carrasco-Zanini J, Pietzner M, Davitte J, Surendran P, Croteau-Chonka DC, Robins C, et al. Proteomic signatures improve risk prediction for common and rare diseases. Nat Med. 2024;30(9):2489–98. https://doi.org/10.1038/s41591-024-03142-z PMID: 39039249

28. Oh HS-H, Rutledge J, Nachun D, Pálovics R, Abiose O, Moran-Losada P, et al. Organ aging signatures in the plasma proteome track health and disease. Nature. 2023;624(7990):164–72. https://doi.org/10.1038/s41586-023-06802-1 PMID: 38057571

29. Wang JJ, Park KS, Dhimal N, Shen S, Tang X, Qu J, et al. Proteomic analysis of retinal mitochondria-associated ER membranes identified novel proteins of retinal degeneration in long-term diabetes. Cells. 2022;11(18):2819. https://doi.org/10.3390/cells11182819 PMID: 36139394

30. Santos FM, Mesquita J, Castro-de-Sousa JP, Ciordia S, Paradela A, Tomaz CT. Vitreous humor proteome: targeting oxidative stress, inflammation, and neurodegeneration in vitreoretinal diseases. Antioxidants (Basel). 2022;11(3):505. https://doi.org/10.3390/antiox11030505 PMID: 35326156

31. Schori C, Trachsel C, Grossmann J, Zygoula I, Barthelmes D, Grimm C. The proteomic landscape in the vitreous of patients with age-related and diabetic retinal disease. Invest Ophthalmol Vis Sci. 2018;59(4):AMD31–40. https://doi.org/10.1167/iovs.18-24122 PMID: 30025106

32. Yang S, Xin Z, Cheng W, Zhong P, Liu R, Zhu Z, et al. Photoreceptor metabolic window unveils eye-body interactions. Nat Commun. 2025;16(1):697. https://doi.org/10.1038/s41467-024-55035-x PMID: 39814712

33. Yang S, Zhu Z, Chen S, Yuan Y, He M, Wang W. Metabolic fingerprinting on retinal pigment epithelium thickness for individualized risk stratification of type 2 diabetes mellitus. Nat Commun. 2023;14(1):6573. https://doi.org/10.1038/s41467-023-42404-1 PMID: 37852995

34. Eldjarn GH, Ferkingstad E, Lund SH, Helgason H, Magnusson OT, Gunnarsdottir K, et al. Large-scale plasma proteomics comparisons through genetics and disease associations. Nature. 2023;622(7982):348–58. https://doi.org/10.1038/s41586-023-06563-x PMID: 37794188

35. Sun BB, Chiou J, Traylor M, Benner C, Hsu Y-H, Richardson TG, et al. Plasma proteomic associations with genetics and health in the UK Biobank. Nature. 2023;622(7982):329–38. https://doi.org/10.1038/s41586-023-06592-6 PMID: 37794186

36. Lim HB, Shin YI, Lee MW, Park GS, Kim JY. Longitudinal changes in the peripapillary retinal nerve fiber layer thickness of patients with type 2 diabetes. JAMA Ophthalmol. 2019;137(10):1125–32. https://doi.org/10.1001/jamaophthalmol.2019.2537 PMID: 31343674

37. Zhong X, Li H, Tan S, Yang S, Zhu Z, Huang W, et al. Initial retinal nerve fiber layer loss and risk of diabetic retinopathy over a four-year period. Invest Ophthalmol Vis Sci. 2024;65(12):5. https://doi.org/10.1167/iovs.65.12.5 PMID: 39365262

38. Liu K, Li T, Zhong P, Zhu Z, Guo X, Liu R, et al. Retinal and choroidal phenotypes across novel subtypes of type 2 diabetes mellitus. Am J Ophthalmol. 2025;269:205–15. https://doi.org/10.1016/j.ajo.2024.08.039 PMID: 39237050

39. Zhang S, Zhu Z, Bulloch G, Guo X, Shang X, Chen Y, et al. Progressive peripapillary choroid thinning and retinal neurodegeneration in patients with diabetes: a 3-year cohort study. Retina. 2022;42(12):2401–10. https://doi.org/10.1097/IAE.0000000000003613 PMID: 36394894

40. Schiborn C, Schulze MB. Precision prognostics for the development of complications in diabetes. Diabetologia. 2022;65(11):1867–82. https://doi.org/10.1007/s00125-022-05731-4 PMID: 35727346

41. Aspelund T, Thornórisdóttir O, Olafsdottir E, Gudmundsdottir A, Einarsdóttir AB, Mehlsen J, et al. Individual risk assessment and information technology to optimise screening frequency for diabetic retinopathy. Diabetologia. 2011;54(10):2525–32. https://doi.org/10.1007/s00125-011-2257-7 PMID: 21792613

42. Hippisley-Cox J, Coupland C. Development and validation of risk prediction equations to estimate future risk of blindness and lower limb amputation in patients with diabetes: cohort study. BMJ. 2015;351:h5441. https://doi.org/10.1136/bmj.h5441 PMID: 26560308

43. Dagliati A, Marini S, Sacchi L, Cogni G, Teliti M, Tibollo V. Machine learning methods to predict diabetes complications. J Diabetes Sci Technol. 2018;12(2):295–302. https://doi.org/10.1177/1932296817706375 PMID: 28494618

44. Basu S, Sussman JB, Berkowitz SA, Hayward RA, Bertoni AG, Correa A, et al. Validation of risk equations for complications of type 2 diabetes (RECODe) using individual participant data from diverse longitudinal cohorts in the U.S. Diabetes Care. 2018;41(3):586–95. https://doi.org/10.2337/dc17-2002 PMID: 29269511

45. Broadbent DM, Wang A, Cheyne CP, James M, Lathe J, Stratton IM, et al. Safety and cost-effectiveness of individualised screening for diabetic retinopathy: the ISDR open-label, equivalence RCT. Diabetologia. 2021;64(1):56–69. https://doi.org/10.1007/s00125-020-05313-2 PMID: 33146763

46. Tanaka S, Tanaka S, Iimuro S, Yamashita H, Katayama S, Akanuma Y, et al. Predicting macro- and microvascular complications in type 2 diabetes: the Japan Diabetes Complications Study/the Japanese Elderly Diabetes Intervention Trial risk engine. Diabetes Care. 2013;36(5):1193–9. https://doi.org/10.2337/dc12-0958 PMID: 23404305

47. Tarasewicz D, Karter AJ, Pimentel N, Moffet HH, Thai KK, Schlessinger D, et al. Development and validation of a diabetic retinopathy risk stratification algorithm. Diabetes Care. 2023;46(5):1068–75. https://doi.org/10.2337/dc22-1168 PMID: 36930723

48. van der Heijden AA, Nijpels G, Badloe F, Lovejoy HL, Peelen LM, Feenstra TL, et al. Prediction models for development of retinopathy in people with type 2 diabetes: systematic review and external validation in a Dutch primary care setting. Diabetologia. 2020;63(6):1110–9. https://doi.org/10.1007/s00125-020-05134-3 PMID: 32246157

49. Wang H, Fu T, Du Y, Gao W, Huang K, Liu Z, et al. Scientific discovery in the age of artificial intelligence. Nature. 2023;620(7972):47–60. https://doi.org/10.1038/s41586-023-06221-2 PMID: 37532811

50. Sun JK, Aiello LP, Abràmoff MD, Antonetti DA, Dutta S, Pragnell M, et al. Updating the staging system for diabetic retinal disease. Ophthalmology. 2021;128(4):490–3. https://doi.org/10.1016/j.ophtha.2020.10.008 PMID: 33218709

51. Fickweiler W, Park H, Park K, Mitzner MG, Chokshi T, Boumenna T, et al. Elevated retinol binding protein 3 concentrations are associated with decreased vitreous inflammatory cytokines, VEGF, and progression of diabetic retinopathy. Diabetes Care. 2022;45(9):2159–62. https://doi.org/10.2337/dc22-0165 PMID: 35852358

52. Chokshi T, Fickweiler W, Jangolla S, Park K, Wu I-H, Shah H, et al. Reduced aqueous retinol-binding protein 3 concentration is associated with diabetic macular edema and progression of diabetic retinopathy. Diabetes Care. 2025;48(1):136–42. https://doi.org/10.2337/dc24-1260 PMID: 39566017

53. Yokomizo H, Maeda Y, Park K, Clermont AC, Hernandez SL, Fickweiler W, et al. Retinol binding protein 3 is increased in the retina of patients with diabetes resistant to diabetic retinopathy. Sci Transl Med. 2019;11(499):eaau6627. https://doi.org/10.1126/scitranslmed.aau6627 PMID: 31270273

54. Jamshidi A, Pelletier J-P, Martel-Pelletier J. Machine-learning-based patient-specific prediction models for knee osteoarthritis. Nat Rev Rheumatol. 2019;15(1):49–60. https://doi.org/10.1038/s41584-018-0130-5 PMID: 30523334

55. Al'Aref SJ, Maliakal G, Singh G, van Rosendael AR, Ma X, Xu Z, et al. Machine learning of clinical variables and coronary artery calcium scoring for the prediction of obstructive coronary artery disease on coronary computed tomography angiography: analysis from the CONFIRM registry. Eur Heart J. 2020;41(3):359–67. https://doi.org/10.1093/eurheartj/ehz565 PMID: 31513271

56. Ruan Y, Bellot A, Moysova Z, Tan GD, Lumb A, Davies J, et al. Predicting the risk of inpatient hypoglycemia with machine learning using electronic health records. Diabetes Care. 2020;43(7):1504–11. https://doi.org/10.2337/dc19-1743 PMID: 32350021

57. Khera R, Haimovich J, Hurley NC, McNamara R, Spertus JA, Desai N, et al. Use of machine learning models to predict death after acute myocardial infarction. JAMA Cardiol. 2021;6(6):633–41. https://doi.org/10.1001/jamacardio.2021.0122 PMID: 33688915

58. Denny JC, Collins FS. Precision medicine in 2030-seven ways to transform healthcare. Cell. 2021;184(6):1415–9. https://doi.org/10.1016/j.cell.2021.01.015 PMID: 33740447

59. Antonetti DA, Silva PS, Stitt AW. Current understanding of the molecular and cellular pathology of diabetic retinopathy. Nat Rev Endocrinol. 2021;17(4):195–206. https://doi.org/10.1038/s41574-020-00451-4 PMID: 33469209

60. Boynton GE, Stem MS, Kwark L, Jackson GR, Farsiu S, Gardner TW. Multimodal characterization of proliferative diabetic retinopathy reveals alterations in outer retinal function and structure. Ophthalmology. 2015;122(5):957–67. https://doi.org/10.1016/j.ophtha.2014.12.001 PMID: 25601533

61. Fernández-Ginés R, Encinar JA, Escoll M, Carnicero-Senabre D, Jiménez-Villegas J, García-Yagüe ÁJ, et al. Specific targeting of the NRF2/β-TrCP axis promotes beneficial effects in NASH. Redox Biol. 2024;69:103027. https://doi.org/10.1016/j.redox.2024.103027 PMID: 38184999

62. Yoshiji S, Lu T, Butler-Laporte G, Carrasco-Zanini-Sanchez J, Su C-Y, Chen Y, et al. Integrative proteogenomic analysis identifies COL6A3-derived endotrophin as a mediator of the effect of obesity on coronary artery disease. Nat Genet. 2025;57(2):345–57. https://doi.org/10.1038/s41588-024-02052-7 PMID: 39856218

63. Oh J, Park C, Kim S, Kim M, Kim C-S, Jo W, et al. High levels of intracellular endotrophin in adipocytes mediate COPII vesicle supplies to autophagosome to impair autophagic flux and contribute to systemic insulin resistance in obesity. Metabolism. 2023;145:155629. https://doi.org/10.1016/j.metabol.2023.155629 PMID: 37302692

64. Zhang S, Wu J, Wang L, Zhang C, Zhang Y, Feng Y. Exploring the hepatic-ophthalmic axis through immune modulation and cellular dynamics in diabetic retinopathy and non-alcoholic fatty liver disease. Hum Genomics. 2025;19(1):19. https://doi.org/10.1186/s40246-025-00730-z PMID: 40011971

65. Tamargo CL, Coca SG, Thiessen Philbrook H, Hu DG, Ix JH, Shlipak MG, et al. The distal nephron biomarkers associate with diabetic kidney disease progression. JCI Insight. 2025;10(12):e186836. https://doi.org/10.1172/jci.insight.186836 PMID: 40548378

66. Puthumana J, Thiessen-Philbrook H, Xu L, Coca SG, Garg AX, Himmelfarb J, et al. Biomarkers of inflammation and repair in kidney disease progression. J Clin Invest. 2021;131(3):e139927. https://doi.org/10.1172/JCI139927 PMID: 33290282

67. Yu H, Zhang J, Qian F, Yao P, Xu K, Wu P, et al. Large-scale plasma proteomics improves prediction of peripheral artery disease in individuals with type 2 diabetes: a prospective cohort study. Diabetes Care. 2025;48(3):381–9. https://doi.org/10.2337/dc24-1696 PMID: 39749935

68. Farrah TE, Pugh D, Chapman FA, Godden E, Balmforth C, Oniscu GC, et al. Choroidal and retinal thinning in chronic kidney disease independently associate with eGFR decline and are modifiable with treatment. Nat Commun. 2023;14(1):7720. https://doi.org/10.1038/s41467-023-43125-1 PMID: 38052813

69. Lovshin JA, Lytvyn Y, Lovblom LE, Katz A, Boulet G, Bjornstad P, et al. Retinopathy and RAAS activation: results from the Canadian Study of Longevity in Type 1 Diabetes. Diabetes Care. 2019;42(2):273–80. https://doi.org/10.2337/dc18-1809 PMID: 30523033

70. Choi JA, Ko S-H, Park YR, Jee D-H, Ko S-H, Park CK. Retinal nerve fiber layer loss is associated with urinary albumin excretion in patients with type 2 diabetes. Ophthalmology. 2015;122(5):976–81. https://doi.org/10.1016/j.ophtha.2015.01.001 PMID: 25666831

71. Rauscher FG, Wang M, Francke M, Wirkner K, Tönjes A, Engel C, et al. Renal function and lipid metabolism are major predictors of circumpapillary retinal nerve fiber layer thickness-the LIFE-Adult Study. BMC Med. 2021;19(1):202. https://doi.org/10.1186/s12916-021-02064-8 PMID: 34488766

72. Guo X, Zhu Z, Bulloch G, Huang W, Wang W. Impacts of Chronic kidney disease on retinal neurodegeneration: a cross-cohort analysis. Am J Ophthalmol. 2024;258:173–82. https://doi.org/10.1016/j.ajo.2023.10.005 PMID: 37820988

73. Ciudin A, Hernández C, Simó-Servat O, Simó R. The usefulness of the retina for identifying people with type 2 diabetes with prodromal stages of dementia. Neurosci Biobehav Rev. 2024;159:105592. https://doi.org/10.1016/j.neubiorev.2024.105592 PMID: 38365136

74. Pedersen FN, Stokholm L, Lois N, Yang D, Cheung CY, Biessels GJ, et al. Structural and metabolic retinal changes associated with mild cognitive impairment in type 2 diabetes. Diabetes. 2023;72(12):1853–63. https://doi.org/10.2337/db23-0025 PMID: 37725903

75. Little K, Llorián-Salvador M, Scullion S, Hernández C, Simó-Servat O, Del Marco A, et al. Common pathways in dementia and diabetic retinopathy: understanding the mechanisms of diabetes-related cognitive decline. Trends Endocrinol Metab. 2022;33(1):50–71. https://doi.org/10.1016/j.tem.2021.10.008 PMID: 34794851

76. Harrison IF, Whitaker R, Bertelli PM, O'Callaghan JM, Csincsik L, Bocchetta M, et al. Optic nerve thinning and neurosensory retinal degeneration in the rTg4510 mouse model of frontotemporal dementia. Acta Neuropathol Commun. 2019;7(1):4. https://doi.org/10.1186/s40478-018-0654-6 PMID: 30616676

77. Chiquita S, Campos EJ, Castelhano J, Ribeiro M, Sereno J, Moreira PI, et al. Retinal thinning of inner sub-layers is associated with cortical atrophy in a mouse model of Alzheimer's disease: a longitudinal multimodal in vivo study. Alzheimers Res Ther. 2019;11(1):90. https://doi.org/10.1186/s13195-019-0542-8 PMID: 31722748

78. Reis A, Mateus C, Melo P, Figueira J, Cunha-Vaz J, Castelo-Branco M. Neuroretinal dysfunction with intact blood-retinal barrier and absent vasculopathy in type 1 diabetes. Diabetes. 2014;63(11):3926–37. https://doi.org/10.2337/db13-1673 PMID: 24947354

79. Jin C-Y, Zheng R, Lin Z-H, Xue N-J, Chen Y, Gao T, et al. Study of the collagen type VI alpha 3 (COL6A3) gene in Parkinson's disease. BMC Neurol. 2021;21(1):187. https://doi.org/10.1186/s12883-021-02215-7 PMID: 33964895

80. Farinas A, Rutledge J, Bot VA, Western D, Ying K, Lawrence KA, et al. Disruption of the cerebrospinal fluid-plasma protein balance in cognitive impairment and aging. Nat Med. 2025;31(8):2578–89. https://doi.org/10.1038/s41591-025-03831-3 PMID: 40665050

81. Dai L, Sheng B, Chen T, Wu Q, Liu R, Cai C, et al. A deep learning system for predicting time to progression of diabetic retinopathy. Nat Med. 2024;30(2):584–94. https://doi.org/10.1038/s41591-023-02702-z PMID: 38177850

82. Li J, Guan Z, Wang J, Cheung CY, Zheng Y, Lim L-L, et al. Integrated image-based deep learning and language models for primary diabetes care. Nat Med. 2024;30(10):2886–96. https://doi.org/10.1038/s41591-024-03139-8 PMID: 39030266

83. Januszewski AS, Velayutham V, Benitez-Aguirre PZ, Craig ME, Cusumano J, Pryke A. Optimal frequency of retinopathy screening in adolescents with type 1 diabetes – Markov modeling approach based on 30 years of data. Diabetes Care. 2022. https://doi.org/10.2337/dc22-0071 PMID: 35975939

84. Simó R, Hernández C, Porta M, Bandello F, Grauslund J, Harding SP, et al. Effects of topically administered neuroprotective drugs in early stages of diabetic retinopathy: results of the EUROCONDOR clinical trial. Diabetes. 2019;68(2):457–63. https://doi.org/10.2337/db18-0682 PMID: 30389750

85. Tang H, Donahoo WT, DeKosky ST, Lee YA, Kotecha P, Svensson M, et al. GLP-1RA and SGLT2i medications for Type 2 diabetes and alzheimer disease and related dementias. JAMA Neurol. 2025;82(5):439–49. https://doi.org/10.1001/jamaneurol.2025.0353 PMID: 40193118
